# KH-type splicing regulatory protein is involved in esophageal squamous cell carcinoma progression

**DOI:** 10.18632/oncotarget.20926

**Published:** 2017-09-15

**Authors:** Yuji Fujita, Kiyoshi Masuda, Junichi Hamada, Katsutoshi Shoda, Takuya Naruto, Satoshi Hamada, Yuko Miyakami, Tomohiro Kohmoto, Miki Watanabe, Rizu Takahashi, Shoichiro Tange, Masako Saito, Yasusei Kudo, Hitoshi Fujiwara, Daisuke Ichikawa, Akira Tangoku, Eigo Otsuji, Issei Imoto

**Affiliations:** ^1^ Department of Human Genetics, Graduate School of Biomedical Sciences, Tokushima University, Tokushima, Japan; ^2^ Division of Digestive Surgery, Department of Surgery, Kyoto Prefectural University of Medicine, Kyoto, Japan; ^3^ Department of Oral Molecular Pathology, Graduate School of Biomedical Sciences, Tokushima University, Tokushima, Japan; ^4^ First Department of Surgery, Faculty of Medicine, University of Yamanashi, Yamanashi, Japan; ^5^ Department of Thoracic, Endocrine Surgery and Oncology, Institute of Biomedical Sciences, Tokushima University Graduate School, Tokushima, Japan

**Keywords:** KHSRP, oncogene, RNA-binding protein, microRNA, esophageal squamous cell carcinoma

## Abstract

KH-type splicing regulatory protein (KHSRP) is a multifunctional RNA-binding protein, which is involved in several post-transcriptional aspects of RNA metabolism, including microRNA (miRNA) biogenesis. It affects distinct cell functions in different tissues and can have an impact on various pathological conditions. In the present study, we investigated the oncogenic functions of KHSRP and their underlying mechanisms in the pathogenesis of esophageal squamous cell carcinoma (ESCC). KHSRP expression levels were elevated in ESCC tumors when compared with those in non-tumorous tissues by immunohistochemistry, and cytoplasmic KHSRP overexpression was found to be an independent prognosticator for worse overall survival in a cohort of 104 patients with ESCC. KHSRP knockdown inhibited growth, migration, and invasion of ESCC cells. KHSRP knockdown also inhibited the maturation of cancer-associated miRNAs, such as miR-21, miR-130b, and miR-301, and induced the expression of their target mRNAs, such as *BMP6, PDCD4,* and *TIMP3,* resulting in the inhibition of epithelial-to-mesenchymal transition. Our findings uncover a novel oncogenic function of KHSRP in esophageal tumorigenesis and implicate its use as a marker for prognostic evaluation and as a putative therapeutic target in ESCC.

## INTRODUCTION

Esophageal cancer (EC) occurs worldwide with a variable geographic distribution. In Asian countries such as Japan, esophageal squamous cell carcinoma (ESCC) is a major histological type of EC and is one of the most aggressive and lethal malignancies [1, 2]. Despite recent clinical advances in EC treatment, the overall patient prognosis remains poor. Therefore, there remains a great deal that needs to be defined, including effective screening, diagnosis, and management strategies to guide the individualized treatment of ESCC.

KH-type splicing regulatory protein (KHSRP) is a multifunctional single-stranded nucleic acid-binding protein located in both the nucleus and the cytoplasm. KHSRP exerts its numerous cellular functions through the modulation of RNA life and gene expression at various levels, such as microRNA (miRNA) maturation, alternative pre-mRNA splicing, and mRNA localization [3]. During miRNA maturation, KHSRP regulates the biogenesis of a subset of miRNAs as a component of both Drosha and Dicer complexes in the nuclear and cytoplasmic compartments, respectively [4, 5]. Various miRNAs have been reported to contribute to multiple tumorigenic processes, including cell proliferation, invasion, and metastasis, through changing the expression of oncogenes and tumor suppressor genes [6]. However, the function of KHSRP seems to vary in different cancers [7–11], and the roles and mechanisms of KHSRP in the tumorigenesis of ESCC remain completely unknown.

In this study, we report that KHSRP exerts oncogenic activity in ESCC cells, at least partly, by inducing the expression of a set of oncogenic miRNAs, including miR-21, miR-130b, and miR-301a, and by suppressing the downstream inhibitors of epithelial-to-mesenchymal transition (EMT), such as BMP6, PDCD4, and TIMP3, resulting in the malignant progression of this disease.

## RESULTS

### KHSRP expression and its association with clinicopathological characteristics of ESCC

**Figure 1:**
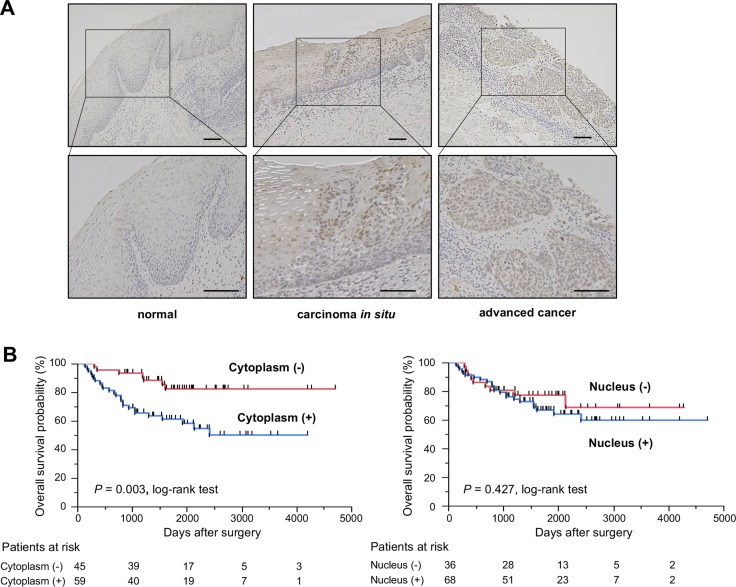
KHSRP protein expression/localization and its association with overall survival in primary ESCC tumors (**A**) Representative images of immunohistochemically detected KHSRP protein in normal mucosa, carcinoma *in situ,* and advanced squamous cell carcinoma of the esophagus. Scale bars, 100 μm. (**B**) Kaplan–Meier curves for the overall survival rates of 104 ESCC patients according to the cytoplasmic (left) and nuclear (right) immunoreactivities of KHSRP.

To determine whether KHSRP is involved in esophageal carcinogenesis, immunohistochemical (IHC) staining with an antibody that specifically recognizes KHSRP was performed in surgically resected esophageal tissues (Figure 1A). KHSRP immunoreactivity was not observed in the cytoplasm and the nucleus of non-tumorous epithelia. In carcinoma *in situ*, KHSRP staining was observed, predominantly in the nucleus, whereas it was observed in both the cytoplasm and the nucleus in advanced ESCC tumors. A similar pattern of KHSRP immunoreactivity was observed in squamous cell carcinomas of other tissues (Supplementary Figure 1A).

We then examined the clinicopathological significance of KHSRP expression in primary ESCC tumors based on the IHC staining patterns. Among the 104 ESCC cases without preoperative chemotherapy, positive cytoplasmic and nuclear KHSRP immunoreactivities were observed in 59 (56.7%) and 68 (65.4%) cases, respectively, based on their intensity scores (Table 1). No significant association was observed between any of the clinicopathological factors and cytoplasmic or nuclear KHSRP immunoreactivity, except for histological grading. However, venous invasion (v) and the depth of tumor invasion (pT) tended to be associated with cytoplasmic and nuclear KHSRP immunoreactivities. Notably, Kaplan–Meier survival estimates showed that positive cytoplasmic KHSRP immunoreactivity was significantly associated with worse overall survival (*P* = 0.003), whereas nuclear KHSRP immunoreactivity was not (Figure 1B). Similarly, positive cytoplasmic KHSRP immunoreactivity tended to be associated with worse recurrence-free survival probability (*P* = 0.053), whereas nuclear KHSRP immunoreactivity was not (Supplementary Figure 1B). In the Cox proportional hazards regression model, cytoplasmic KHSRP immunoreactivity and pT and N stage (pN) categories were statistically significant prognosticators for overall survival by univariate analyses (Table 2). Multivariate analyses showed that cytoplasmic KHSRP immunoreactivity and pT and pN categories were independent predictive factors regardless of the models used, suggesting that overexpressed KHSRP was involved in the development and/or progression of ESCC through cytoplasmic localization. Therefore, we examined the expression level and function of KHSRP in a panel of ESCC cell lines.

**Table 1:** Association between clinicopathological characteristics and KHSRP expression

Clinicopathological factors		*n*	KHSRP immunoreactivity (Cytoplasm)				*P* value^a^	KHSRP immunoreactivity (Nucleus)				*P* value^a^
			High (%)		Low (%)			High (%)		Low (%)		
Total		104	59	(56.7)	45	(43.3)		68	(65.4)	36	(34.6)	
Gender												
	Male	85	46	(54.1)	39	(45.9)	0.3116	54	(63.5)	31	(36.5)	0.5944
	Female	19	13	(68.4)	6	(31.6)		14	(73.7)	5	(26.3)	
Age												
	Mean ± SD (yr)	64.1 ± 8.4	63.2	± 7.6	65.3	± 9.3	0.2031	64.1	± 8.1	64.1	± 9.1	0.9758
Histopathological grading												
	Well and moderately differentiated	75	43	(57.3)	32	(42.7)	1.0000	44	(58.7)	31	(41.3)	**0.0227**
	Poorly differentiated	29	16	(55.2)	13	(44.8)		24	(82.8)	5	(17.2)	
Lymphatic invasion (ly)												
	Negative	45	23	(51.1)	22	(48.9)	0.3263	31	(68.9)	14	(31.1)	0.5397
	Positive	59	36	(61.0)	23	(39.0)		37	(62.7)	22	(37.3)	
Venous invasion (v)												
	Negative	73	37	(50.7)	36	(49.3)	0.0828	44	(60.3)	29	(39.7)	0.1166
	Positive	31	22	(71.0)	9	(29.0)		24	(77.4)	7	(22.6)	
Depth of tumor invasion (pT)												
	pT1	56	27	(48.2)	29	(51.8)	0.0747	32	(57.1)	24	(42.9)	0.0654
	pT2-4	48	32	(66.7)	16	(33.3)		36	(75.0)	12	(25.0)	
N stage (pN)												
	pN0	55	32	(58.2)	23	(41.8)	0.8435	35	(63.6)	20	(36.4)	0.8367
	pN1-3	49	27	(55.1)	22	(44.9)		33	(67.3)	16	(32.7)	
pStage												
	pI	43	23	(53.5)	20	(46.5)	0.6882	24	(55.8)	19	(44.2)	0.0976
	pII-VI	61	36	(59.0)	25	(41.0)		44	(72.1)	17	(27.9)	

Statistically significant values are in boldface type.

^a^*P* values are from χ^2^ or Fisher's exact test and were statistically significant at < 0.05.

**Table 2:** Cox proportional hazard regression analysis for overall survival

Factor	Univariate					Multivariate^a^	
						Model 1	Model 2
	Hazard ratio	95% confidence interval			*P* value	*P* value	*P* value
Gender							
Male versus Female	1.32	0.5552	-	3.9080	0.5518	0.5802	-
Age (years)							
≥ 65 versus < 65	1.06	0.5285	-	2.1734	0.8684	0.2886	-
Histopathological grading							
Poorly versus Well-moderately differentiated	1.27	0.5708	-	2.8320	0.5568	0.5712	-
Lymphatic invasion							
Positive versus Negative	2.03	0.9880	-	4.4942	0.0541	0.6143	-
Venous invasion							
Positive versus Negative	1.61	0.7646	-	3.2637	0.2018	0.8563	-
Depth of tumor invasion (pT)							
pT2-4 versus pT1	3.27	1.5888	-	7.2324	**0.0011**	**0.0332**	**0.0229**
N stage (pN)							
pN1-3 versus pN0	3.06	1.4887	-	6.7720	**0.0021**	**0.0069**	**0.0086**
KHSRP immunoreactivity in the cytoplasm							
Positive versus Negative	3.28	1.4964	-	8.2336	**0.0023**	**0.0045**	**0.0059**
KHSRP immunoreactivity in the nucleus							
Positive versus Negative	1.37	0.6547	-	3.1225	0.4153	-	-

Note: Statistically significant values are in boldface type.

^a^Model 1, all factors excluding KHSRP immunoreactivity in the nucleus model were included;

Model 2, a step-wise procedure was used.

### Involvement of KHSRP in ESCC cell function

*KHSRP* mRNA overexpression was detected in 27 out of the 45 ESCC cell lines when compared with normal esophagus (control) by quantitative real-time PCR (qPCR, Supplementary Figure 2A). In contrast, KHSRP protein overexpression was detected in most ESCC cell lines compared with normal esophageal mucosa, although the pattern of KHSRP protein expression levels was similar to that of *KHSRP* mRNA and discrepancies between mRNA and protein levels were observed in some cell lines to some extent (Supplementary Figure 2B).

**Figure 2:**
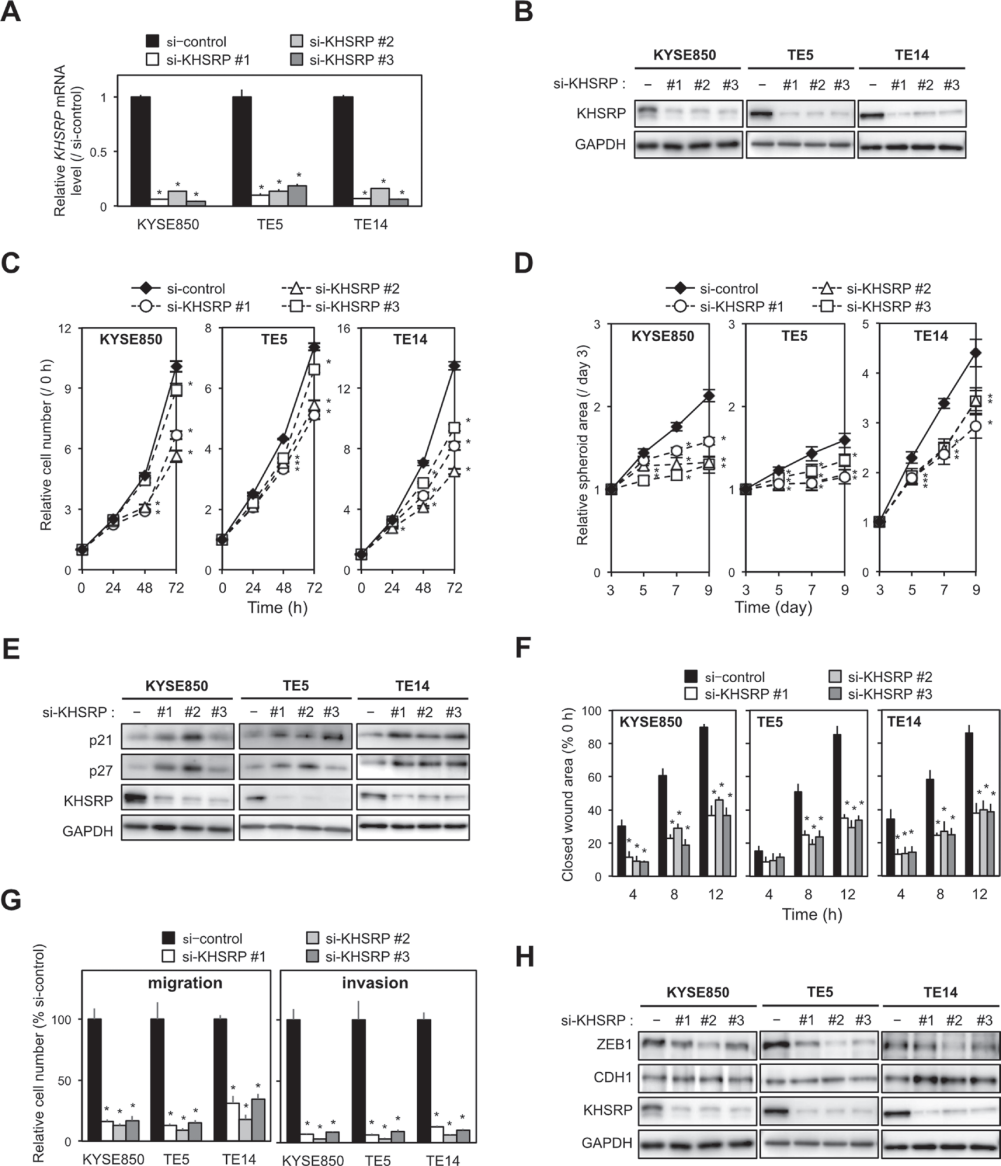
Effects of KHSRP knockdown on cellular function in ESCC cells (**A**) ESCC cells with relatively high expression of KHSRP (KYSE850, TE5, and TE14) were transfected with 10 nM of control or KHSRP-specific siRNAs for 48 h and *KHSRP* mRNA expression levels were evaluated by qPCR. The values are expressed as fold changes (mean ± SD, *n* = 6) when compared with the respective values in control siRNA-transfected cells. ^*^P < 0.05. (**B**) ESCC cells were treated as described in Figure 2A, and expression levels of KHSRP protein were evaluated by Western blot analysis. (**C**) ESCC cells were transfected with 10 nM of control or KHSRP-specific siRNAs for 24 h, and cellular proliferation was measured using a WST-8 assay at the indicated times. The values are expressed as fold changes (mean ± SD, *n* = 6) when compared with the respective values in control cells (0 h). ^*^P < 0.05. (**D**) For spheroid formation assay, ESCC cells treated as described in Figure 2C were seeded in ultra-low attachment 96-well round bottom plates and incubated for the indicated times (d, days). The areas of spheroids were determined as described in the Materials and Methods section (mean ± SD, *n* = 8). ^*^P < 0.05. (**E**) ESCC cells were treated as described in Figure 2A, and the levels of p21^WAF1/Cip1^, p27^Kip1^ and KHSRP proteins were determined by Western blot analysis. (**F**) ESCC cells treated as described in Figure 2C were dispensed into ibidi Culture-Inserts. After 24 h, the Culture-Inserts were removed, and the scratch-wound area was determined as described in the Materials and Methods section (mean ± SD, *n* = 6). The values are expressed as percentages (mean ± SD, *n* = 6) when compared with the respective values in control cells (0 h). ^*^P < 0.05. (**G**) ESCC cells treated as described in Figure 2C were added onto BD Falcon Cell Culture Inserts coated with (invasion assay) or without (migration assay) Matrigel. After incubation for 48 h, cells on the lower surface of filters were determined as described in the Materials and Methods section (mean ± SD, *n* = 6). ^*^P < 0.05. (**H**) ESCC cells were treated as described in Figure 2A, and the expression levels of ZEB1, CDH1, and KHSRP proteins were evaluated by Western blot analysis.

To gain insight into the potential functions of KHSRP, the overexpression of which could contribute to esophageal carcinogenesis, we first tested the effects of KHSRP-specific small interfering RNAs (siRNAs) on cell proliferation using cell lines with relatively high KHSRP expression. By silencing endogenous KHSRP using three different siRNAs (Figure 2A and 2B), cell proliferation was slightly, but significantly, suppressed in ESCC cells (Figure 2C). Knockdown of endogenous KHSRP also inhibited spheroid formation in anchorage-independent *in vitro* 3D cell culture (Figure 2D). Protein levels of cell cycle inhibitors (p21^WAF1/Cip1^ and p27^Kip1^) were increased by knocking down endogenous KHSRP (Figure 2E), although discrepancies between their mRNA and protein levels were observed (Supplementary Figure 3A).

We then assessed the effect of KHSRP knockdown on cell mobility and invasion using scratch-wound healing and Transwell assays. Scratch-wound healing assays revealed that wound closure occurred at a slower rate in KHSRP-knockdown ESCC cells when compared with control cells (Figure 2F and Supplementary Figure 3B). In Transwell assays, uncoated and Matrigel-coated membranes were used to examine cell migration and invasion, respectively. Transwell assays showed that the number of KHSRP siRNA-transfected cells that migrated into or invaded the lower chamber was significantly lower than that of control cells (Figure 2G, Supplementary Figure 3C and 3D). Elevated and reduced expression of the epithelial marker (CDH1) and EMT inducer (ZEB1) proteins, respectively, were observed in KHSRP-knockdown cells when compared with control cells by both Western blot analysis (Figure 2H) and fluorescent immunocytochemical staining (Supplementary Figure 3E).

**Figure 3:**
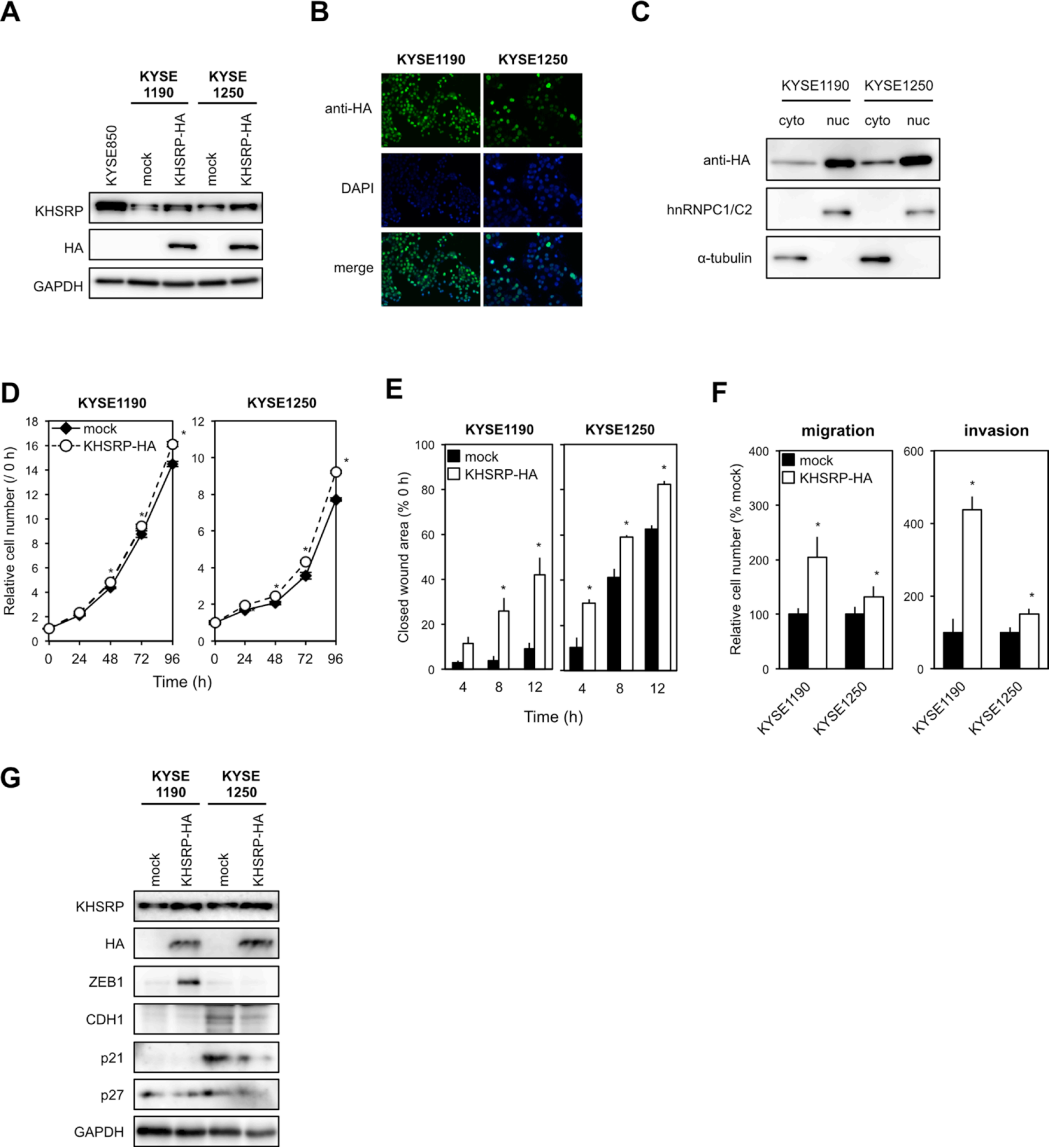
KHSRP overexpression promotes ESCC cell migration and invasion (**A**) Levels of endogenous KHSRP and exogenous HA-tagged KHSRP proteins in ESCC cells with relatively low expression of endogenous KHSRP (KYSE1190 and KYSE1250) that stably express HA-tagged exogenous KHSRP were analyzed by Western blot analysis. (**B**) Representative images of ESCC cells stably expressing HA-tagged exogenous KHSRP detected by FIC with an anti-HA antibody (green). Nuclei were counterstained with DAPI (blue). Scale bars, 20 μm. (**C**) Subcellular distribution of exogenously expressed KHSRP. Cytoplasmic and nuclear fractions were prepared from KYSE1190 and KYSE1250 cells stably expressing HA-tagged KHSRP. Exogenously expressed KHSRP, α-tubulin (cytoplasmic marker), and hnRNPC1/C2 (nuclear marker) were detected by Western blot analysis. (**D**) The number of viable cells of each stable transfectant was assessed using a WST assay at the indicated times. The values are expressed as fold changes (mean ± SD, *n* = 6) when compared with the respective control values (0 h). ^*^P < 0.05. (**E**) Each stable transfectant was dispensed into the ibidi Culture-Inserts. The area of the scratch-wound was determined as described in Figure 2F. The values are expressed as percentages (mean ± SD, *n* = 6) when compared with the respective values in control cells (0 h). ^*^P < 0.05. (**F**) Each stable transfectant was transferred into the upper chamber of BD Falcon Cell Culture Inserts coated with (invasion assay) or without (migration assay) Matrigel. After incubation for 48 h, cells on the lower surface of filters were determined as described in Figure 2G (mean ± SD, *n* = 3). ^*^P < 0.05. (**G**) Levels of endogenous KHSRP, exogenous HA-tagged KHSRP, ZEB1, CDH1, p21^WAF1/Cip1^, and p27^Kip1^ proteins were measured by Western blot analysis.

To determine the chronic effects of exogenously overexpressed KHSRP on ESCC cell function *in vitro*, we established stable transfectants that expressed HA-tagged KHSRP protein using KYSE1190 and KYSE1250 cells with relatively low expression of endogenous KHSRP (Figure 3A and 3B). Western blot analysis using subcellular components obtained by cell fractionation showed that exogenously expressed KHSRP was detected in both nuclear and cytoplasmic lysates, although most KHSRP was located in the nucleus (Figure 3C). Stable expression of KHSRP slightly, but significantly, increased cell proliferation compared with control cells (Figure 3D). Scratch-wound healing assays indicated an induction of faster wound closure in KHSRP-expressing cells compared with control cells (Figure 3E). Transwell assays showed that the number of cells that migrated into or invaded the lower chamber was significantly higher for KHSRP-expressing cells than for control cells under both conditions (Figure 3F). Higher ZEB1 expression and lower p21^WAF1/Cip1^, p27^Kip1^ and/or CDH1 expression were observed in cells exogenously expressing KHSRP, when compared with cells lacking exogenous KHSRP expression (Figure 3G).

### Identification of putative target miRNAs for KHSRP

**Figure 4:**
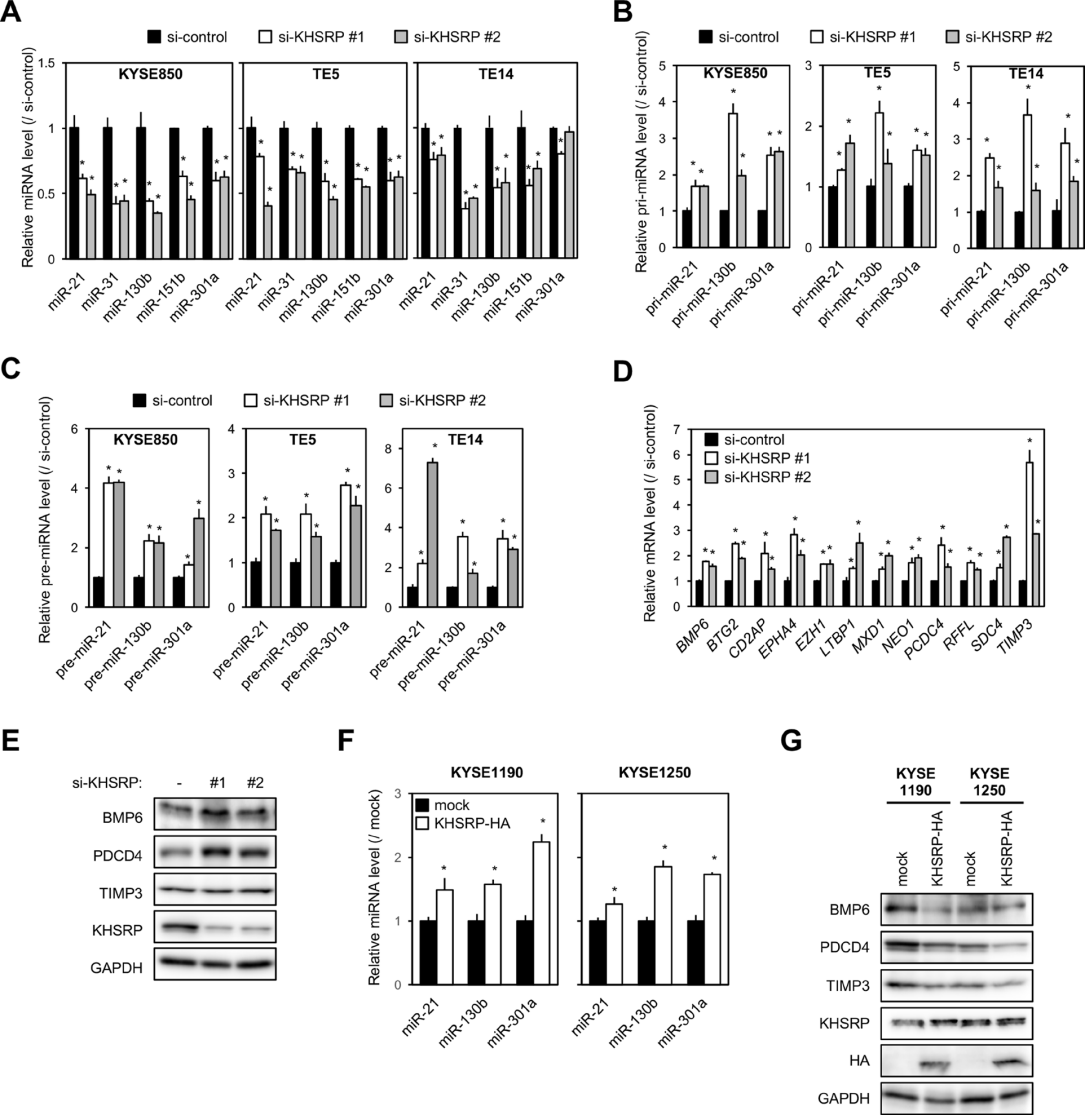
Effects of KHSRP knockdown or overexpression on the expression of putative target miRNAs and protein levels of their target genes in ESCC cells (**A**) ESCC cells were transfected with 10 nM of control or KHSRP-specific siRNAs for 48 h, and the amounts of miR-21, miR-31, miR-130b, miR-151b, and miR-301a were separately measured by qPCR. The values are expressed as fold changes (mean ± SD, *n* = 6) when compared with the respective values in control siRNA-transfected cells. ^*^P < 0.05. (**B**) ESCC cells were treated as described in Figure 4A, and the expression levels of pri-miR-21, pri-miR-130b, and pri-miR-301a were measured by qPCR of control or KHSRP-specific siRNAs. The values are expressed as fold changes (mean ± SD, *n* = 6) when compared with the respective values in control siRNA-transfected cells. ^*^P < 0.05. (**C**) ESCC cells were treated as described in Figure 4A, and expression levels of pre-miR-21, pre-miR-130b, and pre-miR-301a were measured by qPCR of control or KHSRP-specific siRNAs. The values are expressed as fold changes (mean ± SD, *n* = 3) when compared with the respective values in control siRNA-transfected cells. ^*^P < 0.05. (**D**) KYSE850 cells were treated as described in Figure 4A, and the expression levels of the 12 putative target mRNAs for miR-130b, miR-21, and miR-301a were measured by qPCR. The values are expressed as fold changes (mean ± SD, *n* = 6) when compared with the respective values in control siRNA-transfected cells. ^*^P < 0.05. (**E**) KYSE850 cells were treated as described in Figure 4A, and the levels of BMP6, PDCD4, TIMP3, and KHSRP proteins were measured by Western blot analysis. (**F**) The levels of miR-21, miR-130b, and miR-301a in total RNA prepared from ESCC cells overexpressing exogenous KHSRP were measured by qPCR. The values are expressed as fold changes (mean ± SD, *n* = 6) when compared with the respective values in control cells (mock). ^*^P < 0.05. (**G**) The levels of BMP6, PDCD4, TIMP3, endogenous KHSRP, and exogenous HA-tagged KHSRP proteins in lysates prepared from ESCC cells overexpressing exogenous KHSRP were measured by Western blot analysis.

Because KHSRP seemed to exert a greater effect on migration/invasion than on the proliferation of the ESCC cells, we further investigated the molecular mechanisms of KHSRP-promoted ESCC cell migration/invasion. KHSRP has been shown to promote maturation of miRNAs by binding their conserved sequences in the terminal loop [12] and then to alter TGFβ-induced EMT [10]. Therefore, we screened differentially expressed genes (mRNA) and miRNAs in KHSRP-knockdown KYSE850 cells using a microarray analysis. In these cells, 560 and 750 genes were up- and down-regulated, respectively, by > 1.5-fold compared with control cells (Supplementary Tables 1 and 2). KHSRP knockdown also lowered the expression of 26 miRNAs by > 0.5-fold compared with control cells (Table 3). Among these miRNAs, the KHSRP knockdown-induced down-regulation was successfully validated with five cancer-associated miRNAs in three ESCC cell lines (Figure 4A).

**Table 3:** List of the miRNA differentially expressed in KHSRP knockdown cells

miRNA name	Fold change
hsa-miR-130b-3p	0.015
hsa-miR-15a-5p	0.017
hsa-miR-10a-5p	0.019
hsa-miR-324-3p	0.021
hsa-miR-29c-3p	0.022
hsa-miR-26a-5p	0.031
hsa-miR-151b	0.038
hsa-miR-30a-5p	0.039
hsa-miR-185-5p	0.040
hsa-miR-30e-5p	0.044
hsa-miR-181a-5p	0.045
hsa-miR-301a-3p	0.045
hsa-miR-30b-5p	0.047
hsa-miR-18b-5p	0.073
hsa-miR-130a-3p	0.277
hsa-miR-27b-3p	0.319
hsa-miR-22-3p	0.327
hsa-miR-331-3p	0.329
hsa-miR-16-5p	0.340
hsa-miR-15b-5p	0.377
hsa-miR-21-5p	0.388
hsa-miR-31-5p	0.407
hsa-miR-29a-3p	0.410
hsa-miR-31-3p	0.433
hsa-let-7e-5p	0.449
hsa-let-7b-5p	0.466

Three (miR-21, miR-130b, and miR-301a) of these were chosen for further analysis according to their known functions in EMT [13–15]. In 22 KHSRP-overexpressing ESCC cell lines, higher expression levels of miR-21, miR-130b, and miR-301a were detected in 6, 11, and 22 lines, respectively, when compared with the normal esophagus (Supplementary Figure 4A–4C). In KHSRP-knockdown cells, on the other hand, the expression levels of their primary miRNA precursors (pri-miRNAs) and precursor miRNAs (pre-miRNAs) were significantly increased compared with control cells (Figure 4B and 4C). Ribonucleoprotein immunoprecipitation (RIP), which was conducted using nuclear or cytoplasmic lysates prepared from HEK293 cells transiently expressing Halo-tagged KHSRP, followed by qPCR demonstrated the bindings of nuclear and cytoplasmic KHSRP to these pri-miRNAs (Supplementary Figure 5A) and pre-miRNAs (Supplementary Figure 5B), respectively. Taken together, these results suggested that KHSRP is mainly involved in multiple maturation steps of these three miRNAs to increase their expression levels in ESCC.

Among 560 up-regulated genes in KHSRP-knockdown cells, a total of 77 mRNAs were predicted as target genes for at least one of these three miRNAs using DIANA-TarBase v7.0 database (http://diana.imis.athena-innovation.gr/DianaTools/index.php?r=tarbase/index): 40, 39 and 43 mRNAs for miR-21, miR-130b and miR-301a, respectively (Supplementary Table 3). Pathway analysis of these 77 mRNAs using the Kyoto Encyclopedia of Genes and Genomes (KEGG) database ranked “TGFβ signaling pathway” and “endocytosis” as functional pathways with nominal *P*-values < 0.05. However, only “TGFβ signaling pathway” was ranked as a functional pathway with adjusted *P*-value < 0.25 (Table 4). Of the transcripts annotated as “TGFβ signaling pathway” and reported as regulators of EMT, we focused on 12 genes (*BMP6*, *BTG2*, *CD2AP*, *EPHA4*, *EZH1*, *LTBP1*, *MXD1*, *NEO1*, *PDCD4*, *RFFL*, *SDC4* and *TIMP3*) for further analysis according to their known functions in cancers [13, 16–26]. Increased expression levels of these genes in KHSRP-knockdown cells were successfully validated by qPCR (Figure 4D and Supplementary Figure 5C). The effects of the KHSRP silencing on protein expression levels were assessed with *BMP6*, *PDCD4,* and *TIMP3* genes according to their known functions in EMT. Compared with control siRNA-treated cells, KHSRP siRNA-treated cells exhibited higher levels of BMP6, PDCD4, and TIMP3 proteins (Figure 4E and Supplementary Figure 5D). Conversely, cells that stably overexpressed HA-tagged KHSRP showed higher levels of miR-21, miR-130b, and miR-301a (Figure 4F) and lower levels of BMP6, PDCD4, and TIMP3 mRNAs and proteins compared with control cells (Figure 4G and Supplementary Figure 5E). We validated correlations among expression levels of KHSRP, these three miRNAs, and their target mRNAs in surgically resected ESCC tumors, whose high-quality RNA was available for qPCR. In two ESCC tumors with positive cytoplasmic KHSRP immunoreactivity, miR-21, miR-130b, and miR-301a expression levels in tumors were higher and *BMP6*, *PDCD4,* and *TIMP3* mRNA expression levels were lower compared with those in paired non-tumorous tissues, whereas these alterations were not consistently observed in two ESCC tumors with negative cytoplasmic KHSRP immunoreactivity (Supplementary Figure 6A, 6B).

**Table 4:** Functional pathways of 77 target mRNAs of miR-21, miR-130b and miR-301a

Term	*P* value	Adjusted *P* value (FDR)
hsa04350 TGF-beta signaling pathway	0.003	0.117
hsa04144 Endocytosis	0.025	0.388

### Functional association between KHSRP-targeted miRNAs and their putative target mRNAs

**Figure 5:**
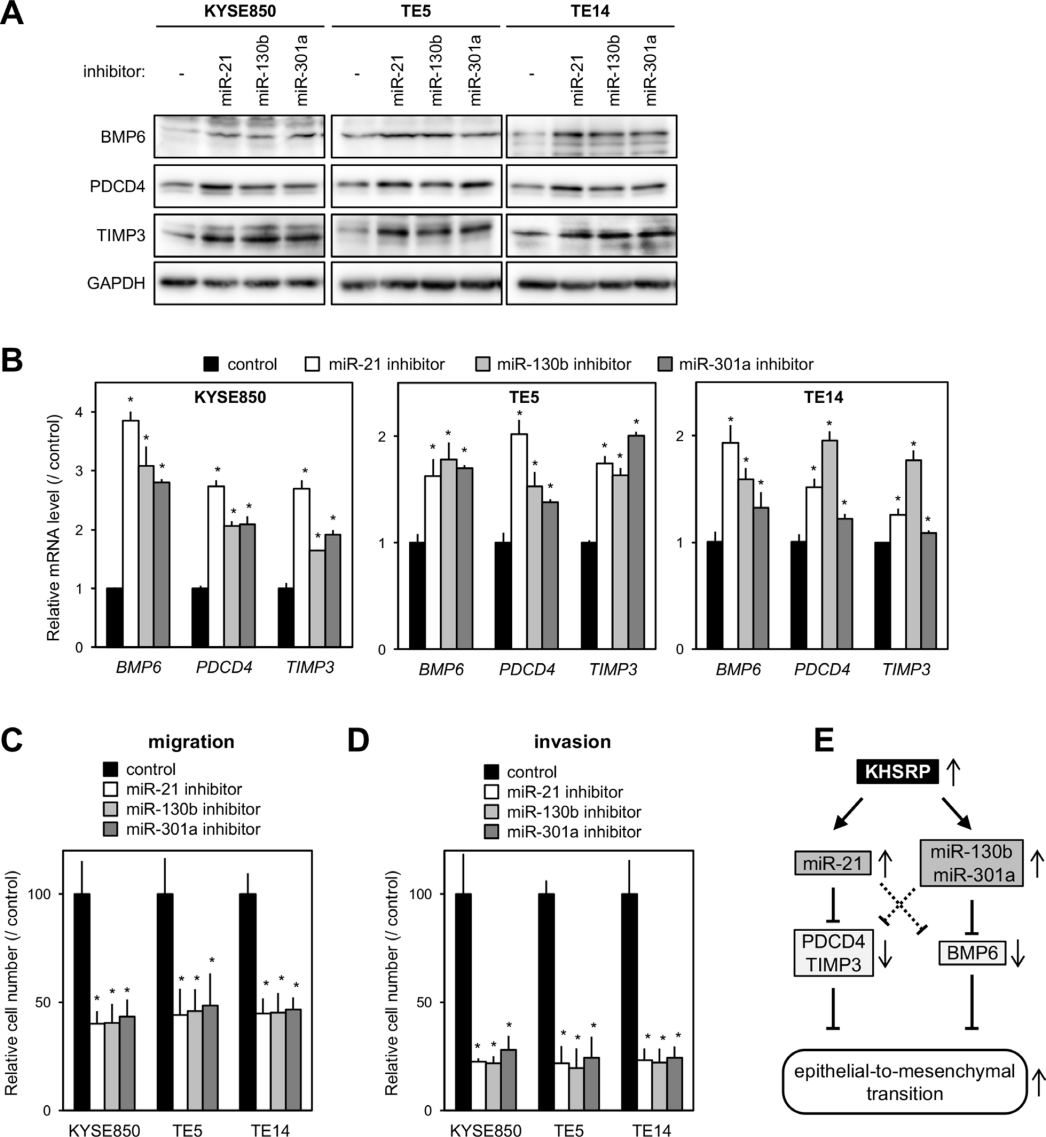
Effects of putative KHSRP target miRNAs on the expression levels of their target mRNAs and ESCC cellular function (**A**) ESCC cells were transfected with 30 nM of each of the miRNA-specific inhibitors or control for 48 h. The levels of BMP6, PDCD4, TIMP3, and KHSRP proteins were measured by Western blot analysis. (**B**) ESCC cells were treated as described in Figure 5A, and the levels of *BMP6*, *PDCD4,* and *TIMP3* mRNAs were measured by qPCR. The values are expressed as fold changes (mean ± SD, *n* = 6) when compared with the respective values in control inhibitor-transfected cells. ^*^P < 0.05. (**C**, **D**) ESCC cells were transfected with 30 nM of each of the miRNA-specific inhibitors or control for 24 h were added onto BD Falcon Cell Culture Inserts coated without (migration assay; C) or with (invasion assay; D) Matrigel. After incubation for 48 h, cells on the lower surface of filters were determined as described in Figure 2G (mean ± SD, *n* = 3). ^*^P < 0.05. (**E**) Model depicts the proposed mechanism whereby KHSRP promotes EMT.

The proteins encoded by *BMP6*, *PDCD4,* and *TIMP3* are known as inhibitors of EMT [13, 16, 26–29]. Therefore, we next tested whether increased KHSRP expression down-regulates their cognate protein levels in ESCC cells by up-regulating miR-21, miR-130b, and/or miR-301a. Exogenous introduction of each of the miRNA inhibitors into cells with relatively high KHSRP expression induced higher levels of BMP6, PDCD4 and/or TIMP3 proteins (Figure 5A) and mRNAs (Figure 5B) and inhibited cell migration (Figure 5C and Supplementary Figure 7A) and invasion (Figure 5D and Supplementary Figure 7B). In contrast, the exogenous introduction of each of the miRNA mimics into KHSRP-knockdown cells restored cell growth (Supplementary Figure 7C) and cell migration (Supplementary Figure 7D), and inhibited the expression levels of BMP6, PDCD4, and TIMP3 proteins (Supplementary Figure 7E), at least in part. These results suggested that overexpressed KHSRP promotes ESCC migration and invasion, at least partly, by the up-regulation of a set of cancer-associated miRNAs and by the suppression of their downstream EMT-inhibiting proteins.

## DISCUSSION

To our knowledge, this is the first report that demonstrates the clinical and functional significance of KHSRP in ESCC tumorigenesis. Overexpression of KHSRP in tumor cells, particularly in the cytoplasm, was an independent prognosticator for overall survival. KHSRP mainly promoted cell migration/invasion with minimal induction of ESCC cell proliferation. Many studies in the past 20 years have demonstrated that KHSRP-dependent regulation of RNA metabolism affects distinct cell functions in different tissues and can have an impact on pathological conditions. However, very few reports have described KHSRP function in the context of human carcinomas. The overall role of KHSRP in carcinogenesis has been controversial, because KHSRP exerts oncogenic or tumor suppressive functions through various mechanisms in different cancer types. It has been reported that KHSRP promotes cell motility in human liver cancer [7, 30] and osteosarcoma [31] cells, and promotes cell proliferation, but not migration, in small cell lung cancer cells [11]. In contrast, Yang *et al.* showed the suppressive effects of KHSRP on migration ability of glioblastoma cells [9]. In addition, two independent studies have demonstrated that high KHSRP expression levels were associated with longer overall survival in glioblastoma multiforme [9, 32]. Because some RNA-binding proteins can act as both positive and negative post-transcriptional regulators in cell type-specific manner [33–36], the finding that KHSRP could work as an oncogene in ESCC was not surprising. It is evident that the consequences of altered KHSRP levels are different in distinct tumors and may reflect cell-restricted functions of KHSRP that rely on the participation of the protein in distinct multiprotein complexes and on its binding to various targets in different cell lineages [3].

miRNAs, the maturation of which is enhanced by KHSRP, play important roles in controlling various cellular functions. Although numerous studies over the past decade have shown cell type-specific changes in miRNA expression profiles, knowledge of the mechanisms that underlie their deregulation in ESCC is still limited [6, 37]. We observed deregulated maturation of the potential target miRNAs with an increase in their precursors in KHSRP-knockdown cells. Some of these have been reported to exert cancer-associated functions [6, 37], indicating that deregulation of KHSRP-miRNA interplay may, at least in part, affect the ESCC-specific miRNA expression profile. It has been demonstrated that KHSRP binds to the conserved G-rich elements in the terminal loop of a cohort of miRNA precursors and promotes their maturation by facilitating their association with Drosha in the nucleus or with Dicer in the cytoplasm [12]. Indeed, the bindings of nuclear and cytoplasmic KHSRP to pri-miRNAs and pre-miRNAs of the three miRNAs, respectively, were detected by our RIP-qPCR experiments. However, none of the three miRNAs that we identified as KHSRP targets in ESCC has typical G-rich elements, such as GGG triplets, in the terminal loop of their precursors. Notably, it has been demonstrated that KHSRP is bound to the precursor of miR-21, and its knockdown reduced the expression levels of mature miR-21 in HeLa and NIH3T3 cells [12]. Therefore, it remains unclear whether KHSRP increases the expression levels of these three miRNAs through known mechanisms.

The comprehensive elucidation of molecules that execute the EMT program still remains an important question in the field of cancer research, because the identification of key molecules within this network is critical from a therapeutic perspective. We demonstrated that KHSRP induced EMT by the up-regulation of oncogenic miRNAs, such as miR-21, miR-130b, and miR-301a. Overexpression of miR-21 is known to induce EMT by targeting *PDCD4* [13, 27, 28] and *TIMP3* [26, 29] mRNAs in various cancer types, including ESCC. EMT-suppressor BMP6 is a known target of miR-130b and miR-301a [38]. Therefore, KHSRP coordinates the expression of a gene cluster that encodes proteins within EMT-related and/or TGFβ signaling pathways. This suggests that KHSRP could be a possible therapeutic target for simultaneous and efficient manipulation of various target genes/pathways contributing to the malignant progression of ESCC. However, in contrast to our findings, several studies have revealed suppressive effects of KHSRP on EMT in murine immortalized mammary epithelial cells [10, 39] and laryngeal squamous cell carcinoma cells [40].

In addition to miRNA maturation, KHSRP plays crucial roles in a variety of cellular processes, such as alternative pre-mRNA splicing and mRNA localization. Therefore, it is possible that oncogenic functions of overexpressed KHSRP in ESCC cells are also mediated through regulation of these processes. In KHSRP-overexpressing ESCC cells, inhibition of each of the endogenously expressed target miRNAs released the up-regulation of the corresponding targets at both mRNA and protein expression levels, and inhibited cell migration/invasion (Figures 5A–5D), whereas introduction of each of the miRNA mimics into KHSRP-knockdown cells inhibited expression levels of the corresponding target proteins and restored cell migration, at least partly. However, each of the three miRNAs decreased expression levels of tested genes other than their possible direct targets in ESCC cells (Figure 5A, Supplementary Figure 7E). These results suggested that the effects of overexpressed KHSRP on EMT in ESCC cells could be explained by direct and indirect miRNA-mediated pathways shown in Figure 5E. In addition, it is possible that KHSRP employs various strategies involving other than these three miRNAs and/or their targets to promote EMT of ESCC, because the effects of KHSRP on the expression of EMT-related molecules, including ZEB1, CDH1, BMP6, PDCD4, and TIMP3, were different among the selected ESCC cell models. KHSRP has been reported to predominantly induce cell proliferation in small cell lung cancer cells by promoting maturation of miR-26a and inhibiting the expression of its target, PTEN [11]. In our study, the effect of KHSRP on proliferation was minimal in ESCC cells, albeit significant. These results suggested that the miRNA maturation-mediated pathway may be important for oncogenic functions of KHSRP regardless of its effects on each malignant phenotype.

In conclusion, our study suggests that overexpression of KHSRP is involved in the pathogenesis of ESCC by inducing the aggressive phenotype of ESCC cells. KHSRP mainly enhances tumor cell migration and invasion by promoting the maturation of miR-130b, miR-21 and miR-301a and by indirectly inhibiting the expression of their targets, such as endogenous EMT inhibitors BMP6, PDCD4 and TIMP3. Our findings uncover a novel oncogenic function of KHSRP in esophageal tumorigenesis and implicate its use as a marker for prognostic evaluation and as a putative therapeutic target in ESCC. However, molecular mechanisms that underlie upstream and downstream pathways of KHSRP-mediated cellular functions in esophageal carcinogenesis remain unclear. In ESCC cell lines, increased expression of the KHSRP protein compared with normal esophagus was more prominently observed than that of the *KHSRP* mRNA, suggesting that undiscovered mechanisms causing hyperinduction of the KHSRP protein, such as enhanced translation and protein stabilization, contribute to overexpression and activation of oncogenic KHSRP in ESCC. Overexpressed KHSRP could bind to miRNA precursors in both the nucleus and the cytoplasm, but cytoplasmic localization of KHSRP seems to be associated with a more malignant phenotype of tumors in patients with ESCC, suggesting that yet undiscovered mechanisms of cytoplasmic KHSRP may also contribute to its tumorigenic functions. Further studies investigating those mechanisms are necessary, not only to develop interventional methods for targeting KHSRP, but also to explore more appropriate potential biomarkers and therapeutic targets for this disease.

## MATERIALS AND METHODS

### Cell lines and primary tissue samples

A total of 45 ESCC cell lines were used, of which 34 belonged to the KYSE cell line series that were established from surgically resected tumors [41] and obtained from Dr. Yutaka Shimada, or were provided by the Japanese Collection of Research Bioresources (JCRB, Ibaraki, Japan); 10 cell lines were from the TE series provided by the Cell Bank, RIKEN BioResource Center (Tsukuba, Japan), and T.T was provided by JCRB.

ESCC tumor samples were obtained from 104 patients with histologically proven primary ESCC, who underwent esophagectomy (potentially curative R0 resection) without neoadjuvant therapy at Kyoto Prefectural University of Medicine Hospital (Kyoto, Japan) between 1998 and 2011 and at Tokushima University Hospital (Tokushima, Japan) between 2005 and 2011. Samples were embedded in paraffin after 24 h of formalin fixation. No patients had synchronous or metachronous multiple cancers in other organs. Relevant clinical and survival data were available for all patients. In this series, all M_1_ tumors had distant lymph node metastases, but no organ metastasis. Disease stage was defined in accordance with the International Union against Cancer tumor–lymph node–metastases classification [42]. The median follow-up period for surviving patients was 40.5 months (ranging from 0.16 to 156.9 months). Formal written consent was obtained from all patients after approval for all aspects of these studies by the local ethics committee (Kyoto Prefectural University of Medicine and Tokushima University).

Total RNA was extracted from frozen tumor tissues and paired non-tumorous tissues using Allprep RNA/DNA mini kit (Qiagen, Hilden, Germany) according to the manufacturer's protocol.

### Antibodies

Antibodies used in this study are listed in Supplementary Table 4.

### Immunohistochemistry and scoring

Paraffin sections (4 μm thick) were subjected to IHC staining for each protein by using DAKO EnVision+ Kit/HRP (Agilent Technologies; Santa Clara, CA, USA) for color development with diaminobenzidine tetrahydrochloride as described in a previous report [43].

Tumor tissues were compared with non-tumorous tissues. The percentage of the total cell population expressing the target protein and the overall staining intensity in tumor cells were evaluated in each case at 200× magnification. The expression of a target protein was considered positive when over 10% of the tumor cells showed strong or diffuse staining. Nuclear KHSRP staining intensity was considered positive when the cells showed stronger staining compared with the non-tumorous esophageal cells in the parabasal layer, whereas the intensity of cytoplasmic KHSRP staining was considered positive when the cells exhibited some staining. All stained slides were evaluated blindly and independently by two investigators without any knowledge of the clinicopathological data, and any discordant results were resolved by using a conference microscope.

### Quantitative real-time PCR for mRNA, pri-miRNA, pre-miRNA, and miRNA

For quantification of mRNA and pri-miRNA levels, 500 ng of total RNA was reverse-transcribed using ReverTra Ace qPCR RT Master Mix with gDNA Remover kit (TOYOBO, Osaka, Japan). Transcript levels were quantified using specific primer sets (Supplementary Table 5) and SYBR Green Master Mix (Applied Biosystems, Waltham, MA, USA), as described in a previous report [43], or TaqMan kits (Supplementary Table 5) according to the manufacturer's instructions. *GAPDH* mRNA levels were used as an internal control for normalization.

For quantification of pre-miRNA levels, 500 ng of total RNA was used as a template to generate the first-strand cDNA for pre-miRNA using miScript II RT kit (Supplementary Table 5; Qiagen). Transcript levels were quantified using miScript Precursor Assay (Qiagen) and miScript SYBR Green PCR Kits (Qiagen) according to the manufacturer's instructions. RNU6 snoRNA levels were similarly measured and used as an internal control for normalization.

For quantification of miRNA levels, 10 ng of total RNA was used as a template to generate specific first-strand cDNA for miRNAs using a TaqMan-specific miRNA reverse transcription kit (Supplementary Table 5; Applied Biosystems). RNU44 snoRNA levels were similarly measured and used as an internal control for normalization.

### Western blot analysis

Whole-cell lysate preparation and Western blot analysis using GAPDH as a loading control were performed as previously described [43]. Images were obtained with a GE Amersham Imager 600 (GE Healthcare, Milwaukee, WI, USA), and band intensities were quantified using ImageQuant TL 8.1 software (GE Healthcare).

Subcellular components were isolated using a LysoPure Nuclear and Cytoplasmic Extractor kit (Wako Pure Chemical Industries, Osaka, Japan) according to the manufacturer's instructions.

### Fluorescent immunocytochemistry (FIC)

Cells were cultured on chamber slides, fixed in 4% paraformaldehyde for 10 min at room temperature and permeabilized with 0.1% Triton X-100 in phosphate-buffered saline for 10 min at room temperature. FIC was performed as described previously [43]. After mounting using ProLong Gold Antifade Reagent with 4′,6-diamidino-2-phenylindole (DAPI), cells were observed under a fluorescence microscope (LSM510; Carl Zeiss, Oberkochen, Germany).

### Plasmid construction

The N-terminal HA-tagged full coding sequence of human *KHSRP* (NM_022037) was amplified by PCR (Supplementary Table 5) using Halo-tagged KHSRP expression plasmid, pFN21AE2201, obtained from Kazusa DNA Research Institute (Chiba, Japan), and cloned into the retroviral vector pMXs-Neo (Cell Biolabs, San Diego, CA, USA).

### Transient transfection experiments

siRNAs targeting mRNA of KHSRP (#1–3), or control siRNAs (Supplementary Table 6) were transfected into cells at a final concentration of 10 nM. The inhibitor for each miRNA or a negative control (Supplementary Table 6) was transfected at a final concentration of 30 nM using Lipofectamine RNAiMax reagent (Invitrogen, Carlsbad, CA, USA) according to the manufacturer's instructions.

### Stable transfection experiments

To establish ESCC cell lines stably overexpressing KHSRP, cells were infected with HA-tagged KHSRP-expressing retroviruses and selected by treatment with 0.5 mg/mL of G418 for 4 weeks. Control cells were obtained using retroviruses obtained from the empty pMXs-Neo vector that was packaged in PLAT-A cells. Crude transfectants were subcultured and tested for exogenous KHSRP overexpression using an anti-HA antibody.

### Cell proliferation

Cell growth was assessed at the indicated times after seeding (2 × 10^4^ cells/24-well plate) using a water-soluble tetrazolium (WST) salt assay (Cell Counting Kit-8; Dojindo, Mashikimachi, Japan) according to the manufacturer's instructions. The results are expressed as the mean absolute absorbance at the indicated time divided by the mean absolute absorbance of each sample cultured for 24 h after seeding.

Spheroid formation was performed as described previously [44]. Briefly, 100 μL/well of cell suspensions (1000 cells/mL) were dispensed into PrimeSurface 96-well round-bottomed plates (Sumitomo Bakelite, Tokyo, Japan). Plates were centrifuged for 5 min at 200 × g and incubated at 37°C in an atmosphere of 5% CO_2_. The areas of formed spheroids were determined using ImageJ software (http://imagej.nih.gov/ij/).

### Scratch-wound healing assay

The transfectants were dispensed into the ibidi Culture-Inserts (ibidi GmbH, Munich, Germany). After 24 h, the Culture-Inserts were removed, and the area of the wound was observed under a microscope. The remaining scratch areas were analyzed with ImageJ software. The migration rate was determined as a percentage reduction of the initial scratch area.

### Transwell migration and invasion assays

Transwell migration and invasion assays were carried out in 24-well modified Boyden chambers (BD transduction, Franklin Lakes, NJ). The upper surface of the 6.4 mm diameter filters with 8 μm pores was pre-coated with (invasion assay) or without (migration assay) Matrigel (BD transduction). Transfectants (5 × 10^4^ cells/well) were transferred into the upper chamber. Following 48 h of incubation, migrated or invasive cells on the lower surface of filters were fixed and stained with Diff-Quik stain (Sysmex, Kobe, Japan). Stained cell nuclei were counted in triplicates. We assessed the migration and invasive potential of each transfectant by calculating the ratio of the percentages to their control counterparts.

### Microarray analysis

Total RNA for gene expression analysis was isolated using RNA-Iso Plus reagent (Takara, Shiga, Japan), whereas total RNA for miRNA expression analysis was isolated using NucleoSpin miRNA kit (Takara). Microarray data for mRNAs and miRNAs were obtained using a whole human genome microarray or a human miRNA microarray (8 × 60 k, Agilent Technologies), respectively, as described previously [43, 45], and analyzed using GeneSpring 13.0 software (Agilent Technologies). All microarray data are available at the Gene Expression Omnibus (GSE99422, GSE99423).

Predicted targets for these differentially expressed miRNAs were identified using TarBase v7.0 (http://www.microrna.gr/tarbase) to provide experimentally validated target genes for miRNAs [46].

Enrichment analyses of the set of the predicted target genes for differentially expressed miRNAs and functional pathways related to this gene set were performed using KEGG pathway analysis. Over-representation of specific KEGG pathways in a gene set was statistically analyzed by DAVID Bioinformatics Resources 6.7 software (http://david.abcc.ncifcrf.gov/home.jsp) [47, 48]. The nominal *P*-value and the Benjamini–Hochberg false discovery rate (adjusted *P*-value) were used to determine the significance of enrichment or over-representation of terms for each annotation.

### Ribonucleoprotein immunoprecipitation (RIP)

Immunoprecipitation of ribonucleoprotein complexes was performed using a RiboCluster Profiler RIP-Assay kit (Medical & Biological Laboratories, Nagoya, Japan) according to the manufacturer's protocol. Briefly, HEK293 cells transiently expressing Halo-tagged KHSRP (KHSRP-Halo) were crosslinked on ice by irradiation with UV light (365 nm) at 150 mJ/cm^2^. Cytoplasmic lysates (250 μg protein) or nuclear extracts (250 μg protein) were incubated for 3 h at 4°C with 50 μL of a 50% (v/v) suspension of HaloLink Resin (Promega, Madison, WI, USA). After the resin was treated with HaloTEV protease (Promega), the IP immunoprecipitated transcripts were quantified by qPCR.

### Statistical analysis

The clinicopathological variables pertaining to the corresponding patients were analyzed by χ^2^ or Fisher's exact test. For survival analysis, Kaplan–Meier survival curves were constructed for groups based on univariate predictors, and differences among the groups were tested with the log-rank test. Univariate and multivariate survival analyses were performed using the likelihood ratio test of the stratified Cox proportional hazards model. Differences among subgroups were tested with Student's *t*-test or with analysis of variance and Tukey's multiple comparison test. Differences were assessed with a two-sided test and considered significant at *P* < 0.05.

## SUPPLEMENTARY MATERIALS FIGURES AND TABLES




