# Supplementary material for: KH-type splicing regulatory protein is involved in esophageal squamous cell carcinoma progression
Source: Oncotarget. 2017 Sep 15;8(60):101130–45. doi: 10.18632/oncotarget.20926 (PMC5731861; doi:10.18632/oncotarget.20926)
Supplement: Supplementary file 1 [file oncotarget-08-101130-s001.pdf]

KH-type splicing regulatory protein is involved in esophageal squamous cell carcinoma progression

SUPPLEMENTARY MATERIALS

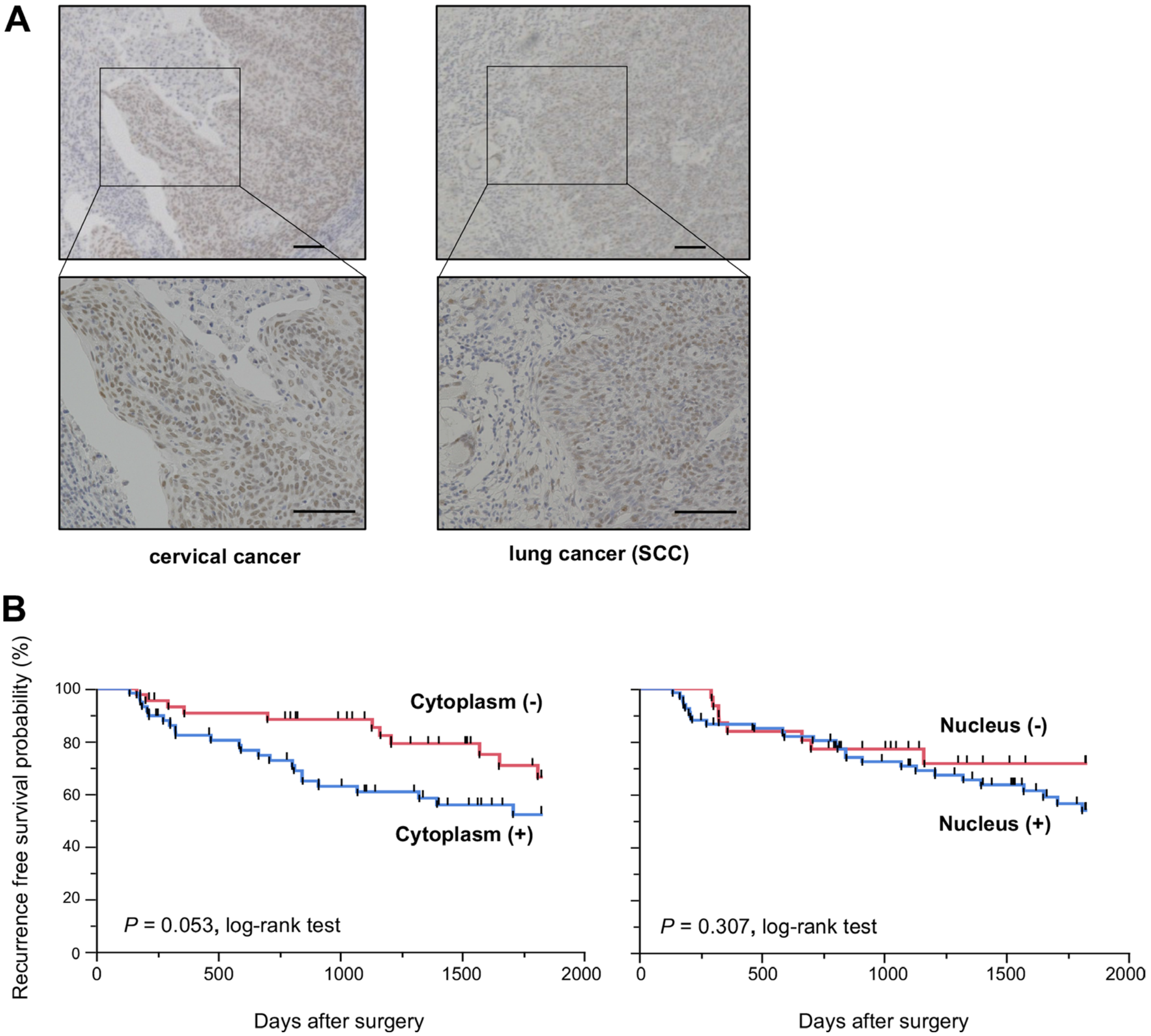

Supplementary Figure 1: Subcellular distribution of KHSRP in cancer tissues and its clinicopathological significance in ESCC cases. (**A**) Immunohistochemical detection of KHSRP in primary squamous cell carcinoma. Sections of cervical and lung squamous cell carcinomas were subjected to immunohistochemical analysis using an anti-KHSRP antibody. Similar to ESCC cells, KHSRP immunoreactivity was observed in both the cytoplasm and the nuclei of lung and cervical tumor cells. Scale bars, 40 μm. (**B**) Kaplan–Meier curves for recurrence-free survival rates of ESCC patients according to the cytoplasmic (left) and nuclear (right) expression levels of KHSRP protein.

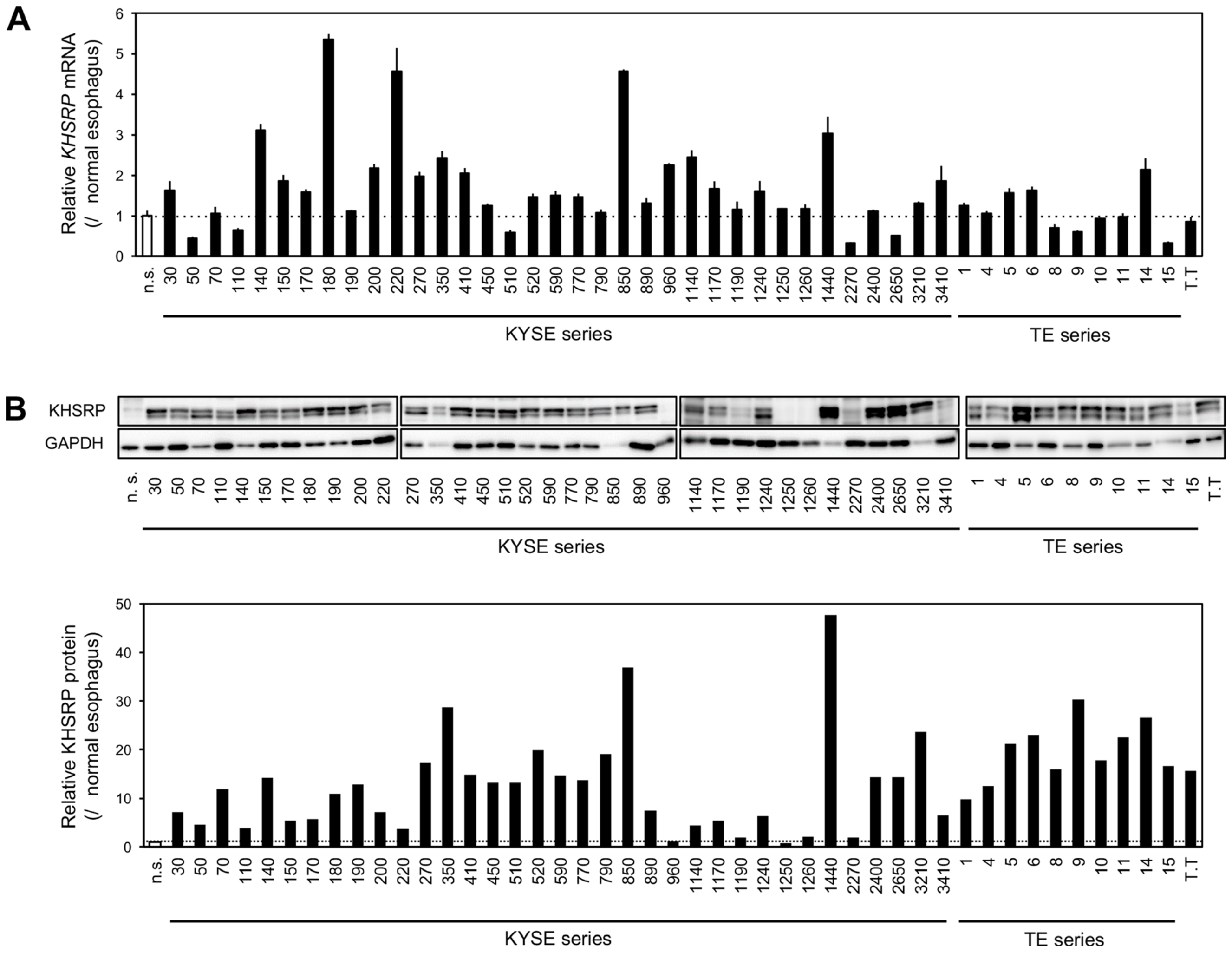

Supplementary Figure 2: KHSRP expression in ESCC cell lines. (**A**) Amounts of *KHSRP* mRNA in a panel of ESCC cell lines were measured by qPCR. The values are expressed as fold changes (mean ± SD, n = 3) when compared with the corresponding value from normal esophagus tissues (control, n.s.). (**B**) KHSRP protein levels in a set of ESCC cell lines were measured by Western blot analysis (upper). The intensities of KHSRP bands were measured with a densitometer using GAPDH protein as an endogenous control and are presented as fold changes when compared with the corresponding value from the normal esophageal tissue (control, n.s.).

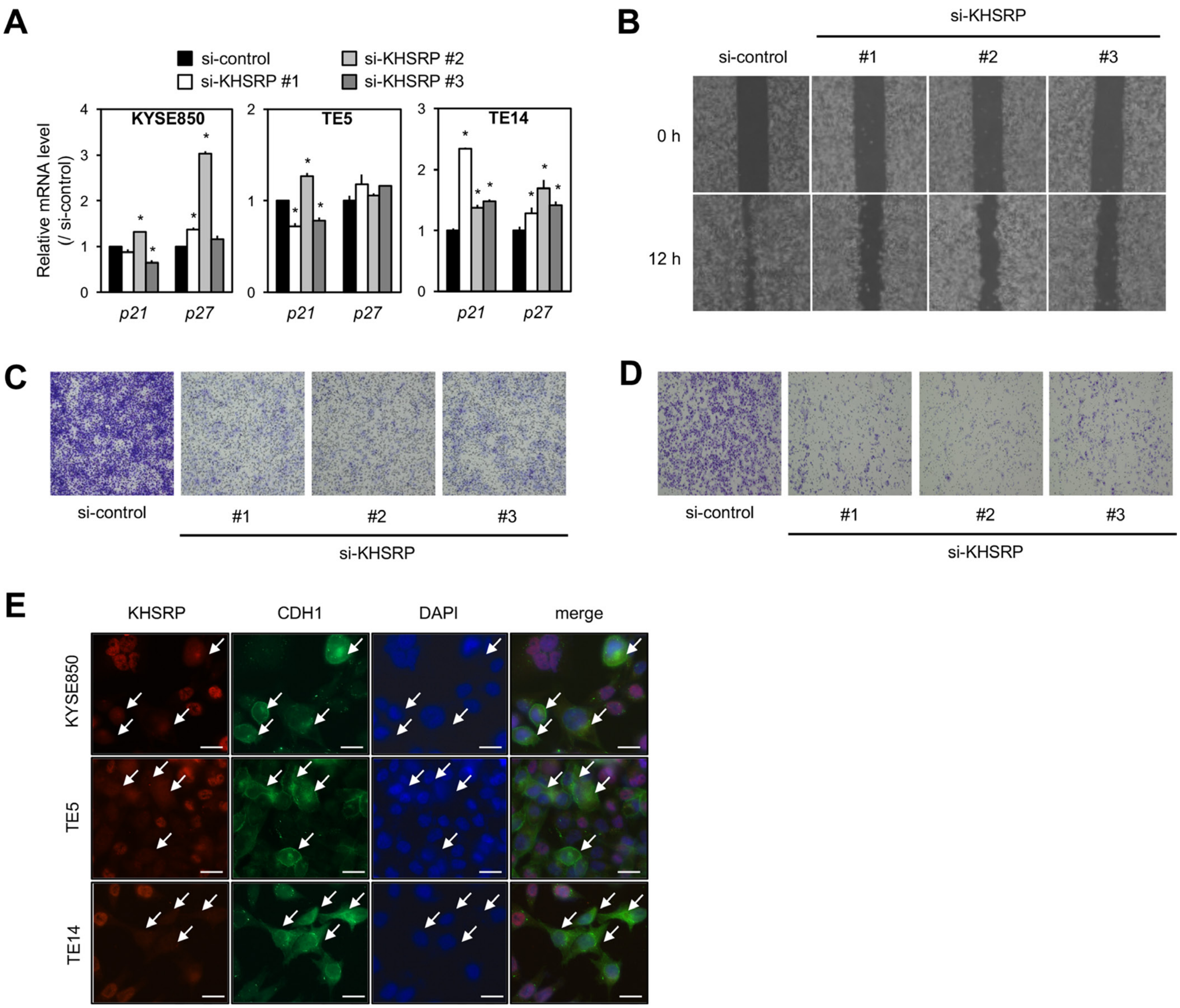

Supplementary Figure 3: Effects of KHSRP knockdown on ESCC cell proliferation and function. (**A**) ESCC cells were transfected with 10 nM of control or KHSRP-specific siRNAs for 48 h, and *p21*$^{\mathrm{WAF1/Cip1}}$ and *p27*$^{\mathrm{Kip1}}$ mRNA expression levels were evaluated by qPCR. The values are expressed as fold changes (mean ± SD, $n = 6$) when compared with the respective values in control siRNA-transfected cells. $^{*}P < 0.05$. (**B**) Representative images of wound healing assays examined in KYSE850 and shown in Figure 2F. Refer to the figure legend of Figure 2F for details. Original magnification 100×. (**C**, **D**) Representative images of migration (C) and invasion (D) assays for experiments shown in Figure 2G. Refer to the figure legend of Figure 2G for details. (**E**) ESCC cells were transfected with 10 nM KHSRP-specific (si-KHSRP #1) siRNA for 48 h and subjected to FIC with anti-CDH1 (green) and anti-KHSRP (red) antibodies. Nuclei were counterstained with DAPI (blue). Cells that effectively lost KHSRP protein expression after siRNA treatment showed higher CDH1 immunoreactivity (arrows), whereas those that retained the expression of endogenous KHSRP protein showed lower CDH1 immunoreactivity. Scale bars, 80 µm.

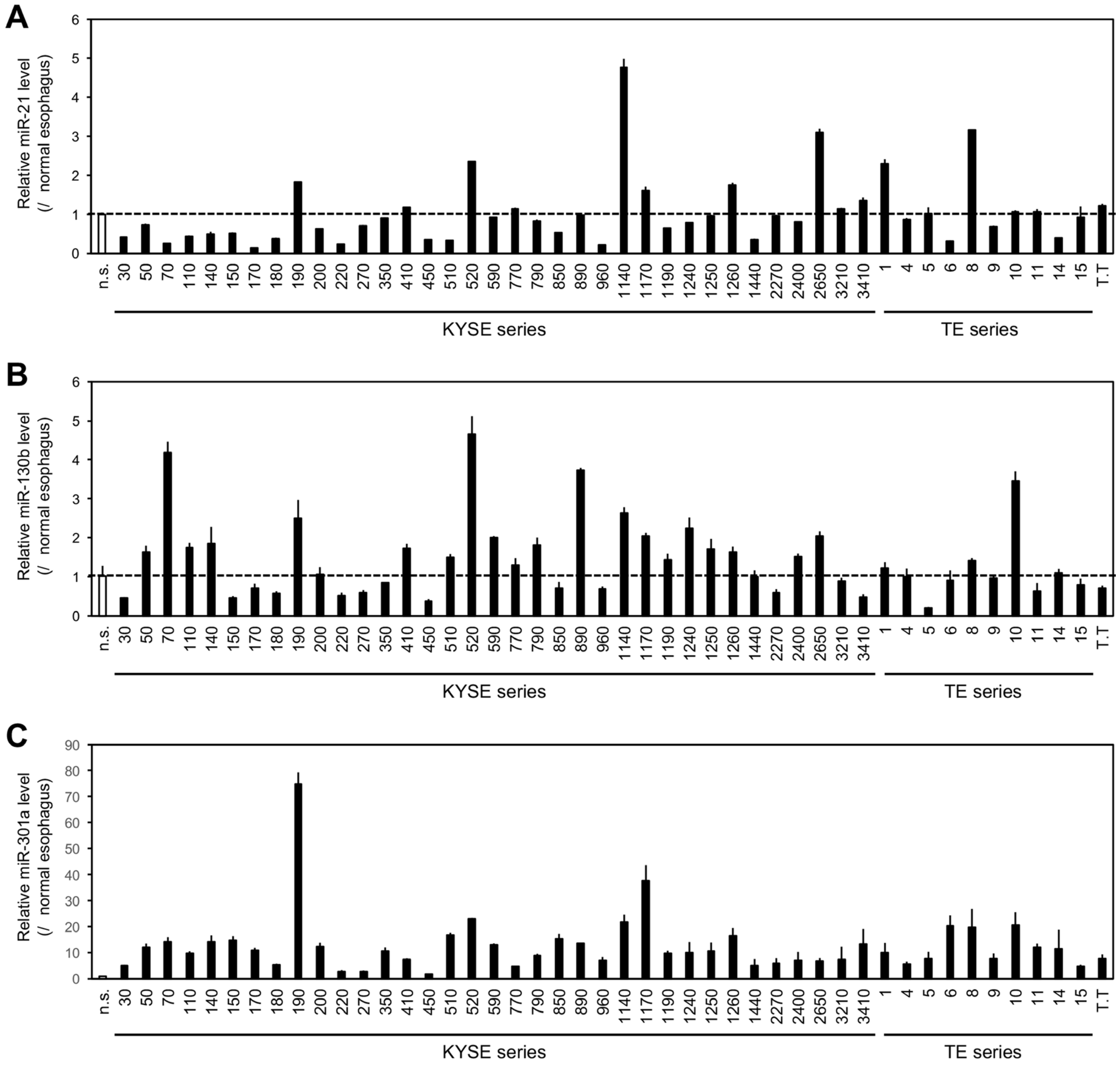

Supplementary Figure 4: miR-21, miR-130b, and miR-301a expression in ESCC cell lines. Expression levels of miR-21 (**A**), miR-130b (**B**), and miR-301a (**C**) in a panel of ESCC cell lines were measured by qPCR. The values are expressed as fold changes (mean ± SD, $n = 3$) when compared with the corresponding values from normal esophageal tissues (control, n.s.).

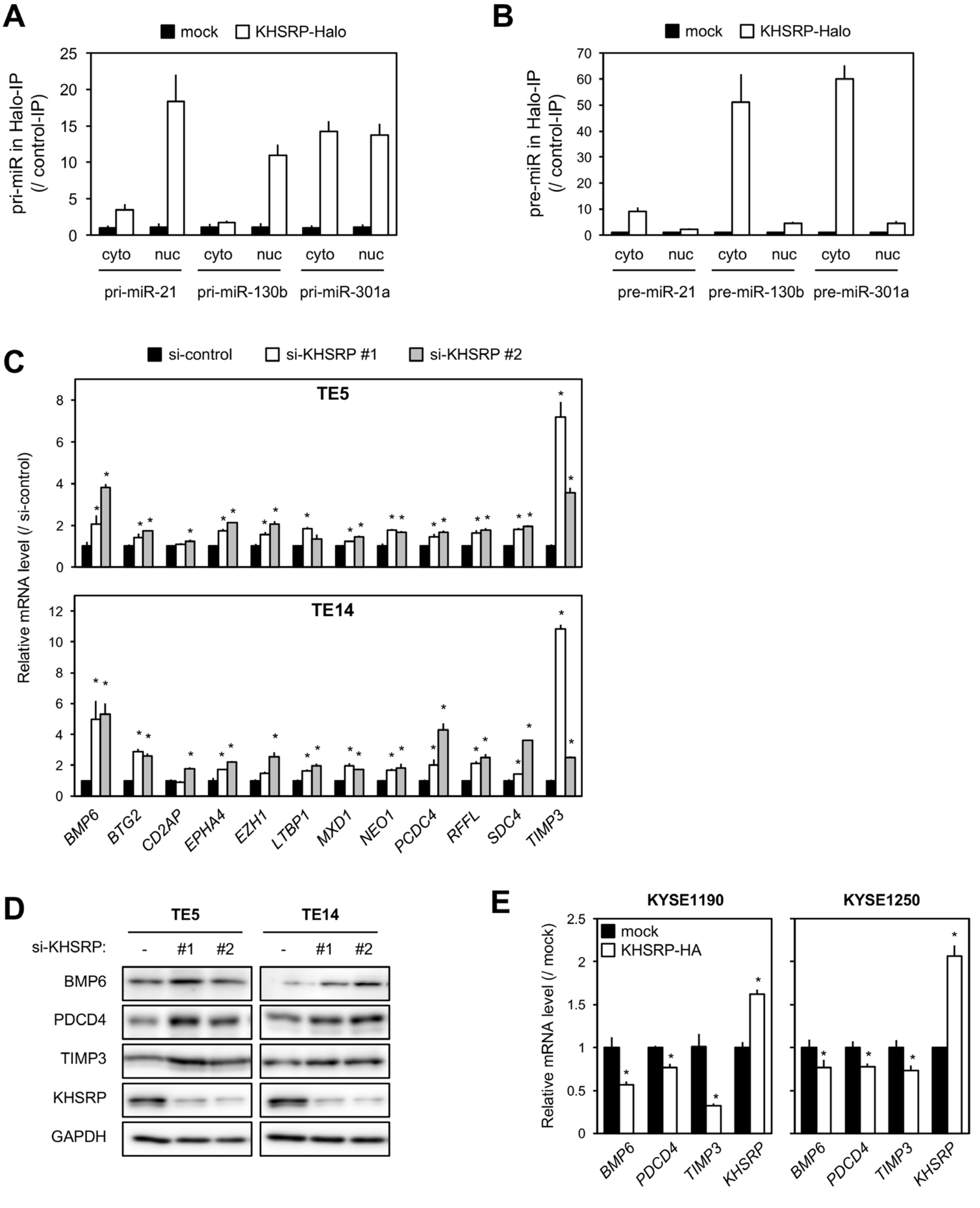

Supplementary Figure 5: Effects of KHSRP on ESCC cellular function. (**A**) HEK293 cells were transiently transfected with KHSRP-Halo or empty vector for 48 h. Binding between KHSRP and each pri-miRNA was measured by RIP using HaloLink Resin and lysates isolated from each subcellular component of HEK293 cells expressing KHSRP-Halo or empty vector, followed by qPCR. Binding is represented as relative enrichment (mean ± SD, n = 3) with respect to background binding (mock). The data were normalized to the levels of *GAPDH* mRNA, an abundant mRNA that is not a target of the KHSRP protein and presents as a low-level co-precipitated contaminant in all IP samples. (**B**) Binding between KHSRP and each pre-miRNA was measured by RIP as described in Supplementary Figure S5A. The data were normalized to the levels of RNU6 snoRNA. Binding is represented as relative enrichment (mean ± SD, n = 3) with respect to background binding (mock). (**C**) Effects of KHSRP silencing on the expression of putative target mRNAs of miR-130b, miR-21, and miR-301a were validated in TE5 (upper) and TE14 (lower) cells. Refer to the figure legend of Figure 4D for details. The values are expressed as fold changes (mean ± SD, n = 6) when compared with the respective values in control siRNA-transfected cells. $^{*}P < 0.05$. (**D**) Effects of KHSRP silencing on the levels of putative target gene expression were validated in TE5 and TE14 cells. Refer to the figure legend of Figure 4E for details. (**E**) Effects of exogenously overexpressed KHSRP on the expression of *BMP6*, *PDCD4,* and *TIMP3* mRNAs in ESCC cells. The values are expressed as fold changes (mean ± SD, n = 6) when compared with the respective values in control cells (mock). $^{*}P < 0.05$.

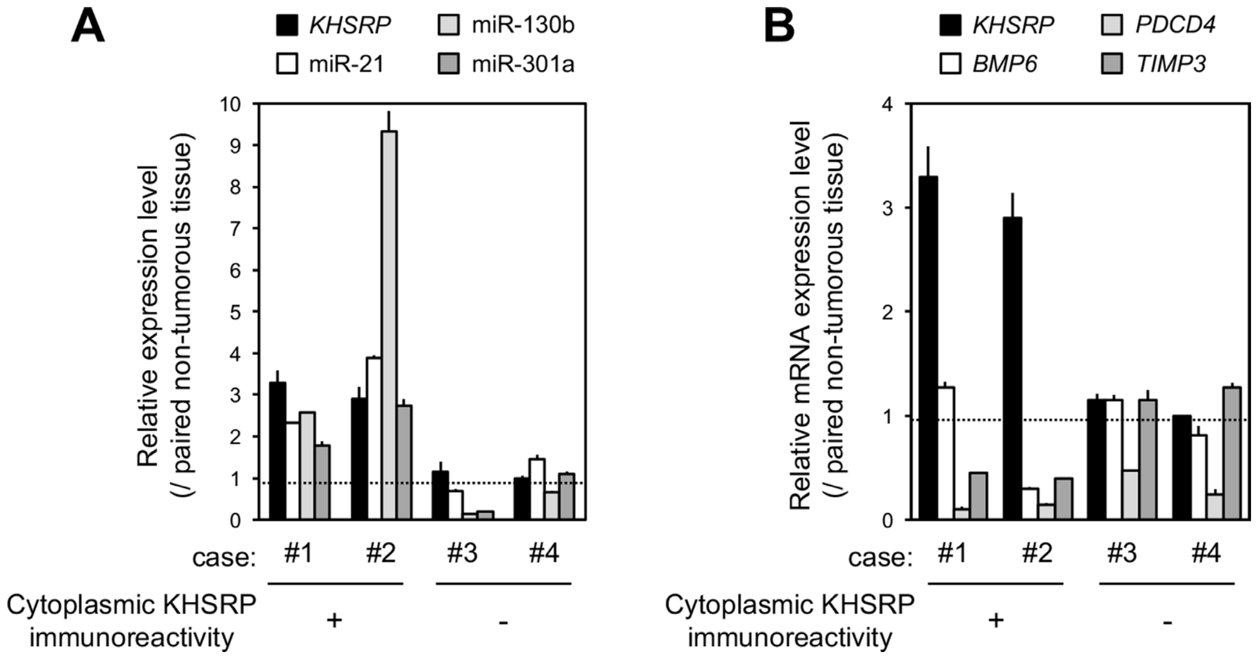

Supplementary Figure 6: Expression patterns of putative target miRNAs for KHSRP and their target genes in ESCC tumors. (**A**) Total RNA was prepared from four frozen ESCC tumor tissues and their paired non-tumorous tissues: two tumors showed positive cytoplasmic KHSRP immunoreactivity (+) and two showed negative cytoplasmic KHSRP immunoreactivity (−). The amounts of *KHSRP* mRNA*,* miR-21, miR-130b, and miR301a were measured by qPCR using *GAPDH* mRNA (for *KHSRP* mRNA) or RNU44 snoRNA (for miR-21, miR-130b, and miR301a) as an endogenous control. The values are expressed as fold changes (mean ± SD, n = 3) compared with the corresponding non-tumorous esophageal tissues. (**B**) Using the total RNAs prepared as described in Supplementary Figure S6A, the amounts of *KHSRP*, *BMP6*, *PDCD4,* and *TIMP3* mRNAs were measured by qPCR using *GAPDH* mRNA as an endogenous control. The values are expressed as fold changes compared with corresponding non-tumorous esophageal tissues.

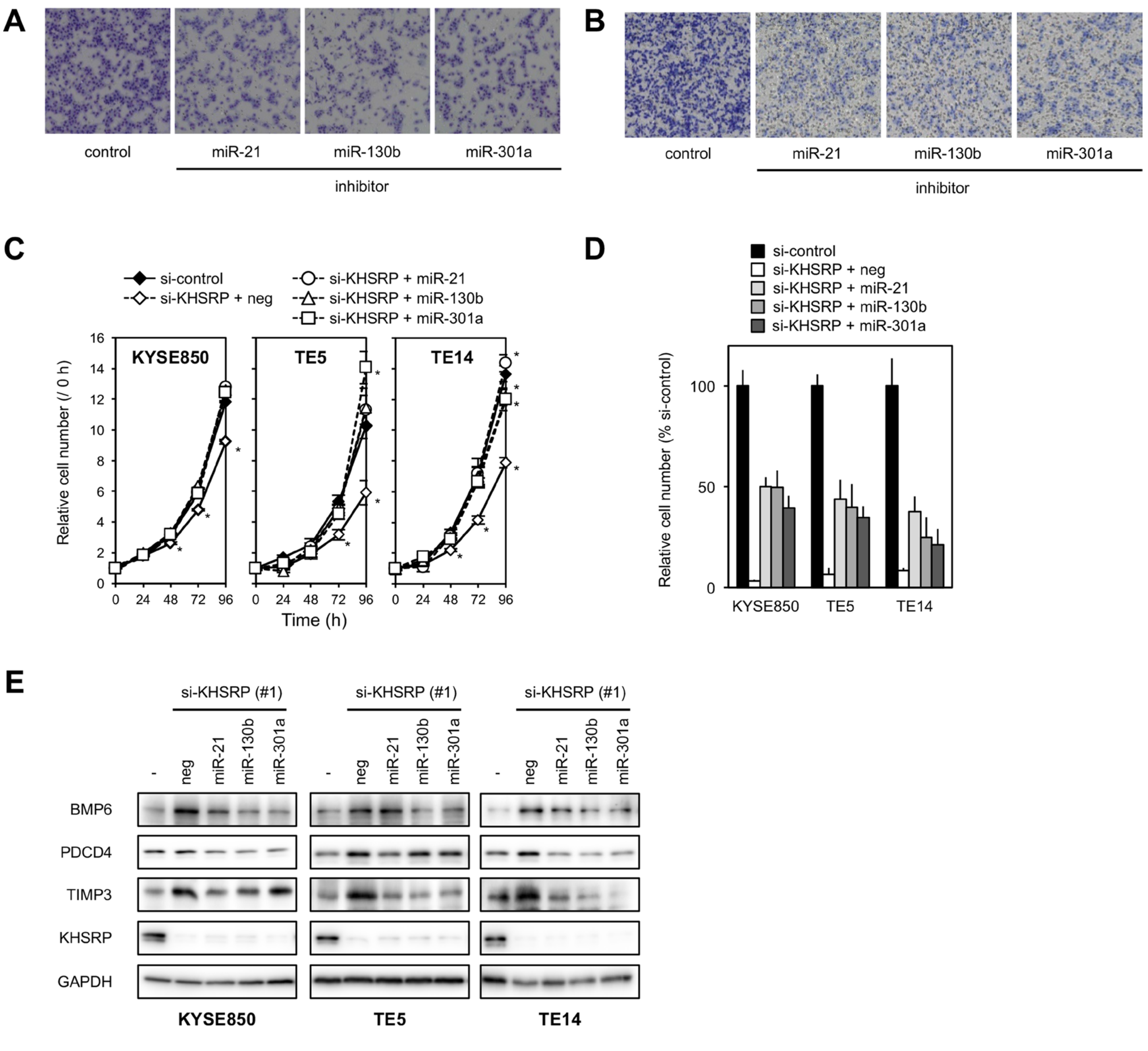

Supplementary Figure 7: Effects of inhibition or overexpression of putative target miRNAs on ESCC cellular function. (**A**, **B**) Representative images of migration (A) and invasion (B) assays for experiments shown in Figures 5C and 5D, respectively. Refer to the figure legends of Figure 5C and 5D for details. (**C**) ESCC cells were transfected with 20 nM control siRNA alone or co-transfected with 10 nM KHSRP-specific siRNA (si-KHSRP #1) and 10 nM each of miRNA mimic or control mimic (negative control, neg), and the number of viable cells of each stable transfectant was assessed using a WST assay at the indicated times. The values are expressed as fold changes (mean ± SD, n = 4) when compared with the respective control values (0 h). $^{*}P < 0.05$ vs. si-control transfected cells. (**D**) ESCC cells treated as described in Supplementary Figure S7C were added onto BD Falcon Cell Culture Inserts coated without Matrigel. After incubation for 48 h, cells on the lower surface of filters were determined as described in the Materials and Methods section (mean ± SD, n = 6). (**E**) ESCC cells treated as described in Supplementary Figure 7C. After incubation for 48 h, levels of BMP6, PDCD4, TIMP3, and KHSRP proteins were determined by Western blot analysis using GAPDH as a loading control.

Supplementary Table 1: List of the upregulated genes in KHSRP knockdown cells. See Supplementary_Table_1

Supplementary Table 2: List of the downregulated genes in KHSRP knockdown cells. See Supplementary_Table_2

Supplementary Table 3: List of the predicted mRNAs for each miRNA. See Supplementary_Table_3

Supplementary Table 4: List of antibodies used in this study

Antibody name	Vender[a]	ID	Purpose[b]
anti-KHSRP	ATLAS antibodies	HPA034739	IHC (1:500 dilution)/FIC (1:500 dilution)/ Western blotting
anti-CDH1	BD Transduction Laboratories	610181	FIC (1:100 dilution)/Western blotting
anti-ZEB1	Cell Signaling Technology	D80D3	Western blotting
anti-TIMP3	Cell Signaling Technology	D74B10	Western blotting
anti-PDCD4	Cell Signaling Technology	D29C6	Western blotting
anti-BMP6	R&D Systems	74219	Western blotting
anti-GAPDH	Santa Cruz Biotechnology	0411	Western blotting
anti-FLAG (DDDDK)	Medical & Biological Laboratories	FLA-1	FIC(1:20000 dilution)/ Western blotting
anti-p27	Medical & Biological Laboratories	DCS-72	Western blotting
anti-p21 antibody	Medical & Biological Laboratories	DCS-60	Western blotting
Alexa Fluor 488-labeled goat anti-mouse	Molecular Probes		FIC (1:500 dilution)
Alexa Fluor 594 goat anti-rabbit	Molecular Probes		FIC (1:500 dilution)
Alexa Fluor 594 donkey anti-goat	Molecular Probes		FIC (1:500 dilution)

[a]ATLAS antibodies, Stockholm, Sweden; BD Transduction Laboratories, Lexington, KY, USA; Cell Signaling Technology, Danvers, MA, USA; R&D Systems, Minneapolis, MN, USA; Santa Cruz Biotechnology, Santa Cruz, CA, USA; Medical & Biological Laboratories, Nagoya, Japan; Molecular Probes, Eugene, OR, USA.
[b]IHC, immunohistochemistry; FIC, fluorescence immunocytochemistry.

Supplementary Table 5: List of primer sets used in PCR and qPCR. See Supplementary_Table_5

Supplementary Table 6: List of siRNAs, miRNA inhibitor and miRNA mimic for silencing target genes and silencing/overexpressing miRNAs

siRNAs

Gene name	siRNA name	Vender	ID	Sequence
KHSRP	#1	Ambion	s16322	5′-GGAUUCAGGCUGCAAAGUATT-3′
	#2	Sigma-Aldrich		5′-GAUGAUGCUGGAUGACAUUTT-3′
	#3	SantaCruz	s14133	consist of pools of three to five target-specific siRNAs
control siRNA		Ambion	4390846	Silencer Select Negative Control #2
		Sigma-Aldrich	SIC-001	Universal Negative Control #1

miRNA inhibitors

miRNA name	Vender	ID	
has-miR130	Ambion	MH10777	
has-miR-21	Ambion	MH10206	
has-miR-301	Ambion	MH10978	
Negative control	Ambion	4464076	mirVana miRNA Inhibitor, Negative Control #1

miRNA mimics

miRNA name	Vender	ID	
has-miR130	Ambion	MC10777	
has-miR-21	Ambion	MC10206	
has-miR-301	Ambion	MC10978	
Negative control	Ambion	4464058	mirVana miRNA Mimic, Negative Control #1
