# Supplementary material for: KH-type splicing regulatory protein is involved in esophageal squamous cell carcinoma progression
Source: Oncotarget. 2017 Sep 15;8(60):101130–45. doi: 10.18632/oncotarget.20926 (PMC5731861; doi:10.18632/oncotarget.20926)
Supplement: Supplementary file 2 [file oncotarget-08-101130-s002.doc]

**Supplementary Table S1. List of the upregulated genes in KHSRP knockdown cells**

| **Gene Symbol** | **Gene Name** | **Probe ID** | **Fold change (/control siRNA)** |
| --- | --- | --- | --- |
| GLYCTK | glycerate kinase | A_33_P3285580 | 4.65 |
| NR2E3 | nuclear receptor subfamily 2, group E, member 3 | A_23_P205867 | 4.39 |
| KCNE5 | potassium channel, voltage gated subfamily E regulatory beta subunit 5 | A_23_P256641 | 4.15 |
| lnc-EYS-2 | lnc-EYS-2:1 | A_32_P120791 | 4.06 |
| RNF165 | ring finger protein 165 | A_33_P3319680 | 3.83 |
| LOC101927043 | uncharacterized LOC101927043 | A_19_P00317519 | 3.74 |
| SPRYD4 | SPRY domain containing 4 | A_23_P411612 | 3.63 |
| TMEM229B | transmembrane protein 229B | A_33_P3355014 | 3.31 |
| TLR5 | toll-like receptor 5 | A_23_P85903 | 2.90 |
| LOC283075 | uncharacterized LOC283075 | A_33_P3648597 | 2.89 |
| AQP7P1 | aquaporin 7 pseudogene 1 | A_33_P3245290 | 2.88 |
| MYO15B | myosin XVB pseudogene | A_24_P369898 | 2.86 |
| lnc-RNF208-1 | lnc-RNF208-1:1 | A_33_P3259938 | 2.84 |
| IL6R | interleukin 6 receptor | A_33_P3288844 | 2.76 |
| CHST1 | carbohydrate (keratan sulfate Gal-6) sulfotransferase 1 | A_23_P98205 | 2.71 |
| MPRIP | myosin phosphatase Rho interacting protein | A_23_P15348 | 2.71 |
| OAS2 | 2'-5'-oligoadenylate synthetase 2, 69/71kDa | A_33_P3225512 | 2.70 |
| RPS29 | ribosomal protein S29 | A_33_P3282840 | 2.69 |
| LOC100129534 | small nuclear ribonucleoprotein polypeptide N pseudogene | A_33_P3271395 | 2.69 |
| NRP1 | neuropilin 1 | A_24_P135322 | 2.68 |
| PLA2G12A | phospholipase A2, group XIIA | A_24_P706752 | 2.67 |
| CNN1 | calponin 1, basic, smooth muscle | A_23_P125233 | 2.66 |
| MMP11 | matrix metallopeptidase 11 (stromelysin 3) | A_23_P57417 | 2.64 |
| ANKHD1 | ankyrin repeat and KH domain containing 1 | A_23_P92642 | 2.63 |
| UAP1L1 | UDP-N-acetylglucosamine pyrophosphorylase 1 like 1 | A_23_P135164 | 2.63 |
| ADAMTS7P1 | ADAMTS7 pseudogene 1 | A_33_P3261828 | 2.60 |
| SLX1A | SLX1 structure-specific endonuclease subunit homolog A (S. cerevisiae) | A_23_P501451 | 2.59 |
| LAD1 | ladinin 1 | A_33_P3303810 | 2.59 |
| lnc-C9orf69-2 | lnc-C9orf69-2:3 | A_19_P00316344 | 2.58 |
| SYNPO | synaptopodin | A_33_P3397658 | 2.58 |
| G3BP1 | GTPase activating protein (SH3 domain) binding protein 1 | A_33_P3331237 | 2.57 |
| MAOB | monoamine oxidase B | A_23_P85015 | 2.57 |
| DHRS11 | dehydrogenase/reductase (SDR family) member 11 | A_23_P27005 | 2.55 |
| PAQR6 | progestin and adipoQ receptor family member VI | A_23_P97283 | 2.52 |
| PROC | protein C (inactivator of coagulation factors Va and VIIIa) | A_33_P3379922 | 2.52 |
| SQRDL | sulfide quinone reductase-like (yeast) | A_23_P3221 | 2.51 |
| KIF16B | kinesin family member 16B | A_33_P3233135 | 2.48 |
| TMEM106A | transmembrane protein 106A | A_32_P101860 | 2.46 |
| VWA1 | von Willebrand factor A domain containing 1 | A_24_P122746 | 2.42 |
| MPRIP | myosin phosphatase Rho interacting protein | A_33_P3230528 | 2.41 |
| GALNT3 | polypeptide N-acetylgalactosaminyltransferase 3 | A_24_P114249 | 2.40 |
| BLACAT1 | bladder cancer associated transcript 1 (non-protein coding) | A_19_P00319809 | 2.40 |
| BLACAT1 | bladder cancer associated transcript 1 (non-protein coding) | A_19_P00319808 | 2.39 |
| ALOX12 | arachidonate 12-lipoxygenase | A_23_P152906 | 2.38 |
| HIC2 | hypermethylated in cancer 2 | A_33_P3379017 | 2.37 |
| POLR1A | polymerase (RNA) I polypeptide A, 194kDa | A_33_P3320533 | 2.34 |
| DDTL | D-dopachrome tautomerase-like | A_33_P3340205 | 2.34 |
| IL15RA | interleukin 15 receptor, alpha | A_33_P3399267 | 2.34 |
| CDKN1C | cyclin-dependent kinase inhibitor 1C (p57, Kip2) | A_23_P428129 | 2.33 |
| C14orf132 | chromosome 14 open reading frame 132 | A_19_P00321588 | 2.33 |
| APLN | apelin | A_23_P113393 | 2.33 |
| CLDN15 | claudin 15 | A_23_P500886 | 2.32 |
| TIMP3 | TIMP metallopeptidase inhibitor 3 | A_23_P399078 | 2.32 |
| ZNF503-AS1 | ZNF503 antisense RNA 1 | A_33_P3569555 | 2.31 |
| POLR2J4 | polymerase (RNA) II (DNA directed) polypeptide J4, pseudogene | A_33_P3308167 | 2.31 |
| IL9R | interleukin 9 receptor | A_23_P34066 | 2.31 |
| LAMB2P1 | laminin, beta 2 pseudogene 1 | A_33_P3413815 | 2.27 |
| NECAB2 | N-terminal EF-hand calcium binding protein 2 | A_23_P66011 | 2.27 |
| PDCD4 | programmed cell death 4 (neoplastic transformation inhibitor) | A_33_P3212092 | 2.27 |
| LINC00982 | long intergenic non-protein coding RNA 982 | A_33_P3261463 | 2.26 |
| SFXN2 | sideroflexin 2 | A_24_P175612 | 2.26 |
| FAM178A | family with sequence similarity 178, member A | A_33_P3392245 | 2.24 |
| PLD1 | phospholipase D1, phosphatidylcholine-specific | A_33_P3320062 | 2.24 |
| PACS2 | phosphofurin acidic cluster sorting protein 2 | A_24_P393372 | 2.24 |
| POU3F4 | POU class 3 homeobox 4 | A_23_P114210 | 2.24 |
| LOC643441 | uncharacterized LOC643441 | A_33_P3401307 | 2.23 |
| PTGR2 | prostaglandin reductase 2 | A_23_P48713 | 2.22 |
| GJB2 | gap junction protein, beta 2, 26kDa | A_23_P204947 | 2.21 |
| VWA9 | von Willebrand factor A domain containing 9 | A_33_P3244921 | 2.21 |
| PRODH | proline dehydrogenase (oxidase) 1 | A_33_P3338341 | 2.21 |
| FBXL22 | F-box and leucine-rich repeat protein 22 | A_33_P3277674 | 2.20 |
| LHX1 | LIM homeobox 1 | A_33_P3409854 | 2.20 |
| FAM117B | family with sequence similarity 117, member B | A_32_P195401 | 2.19 |
| ORAI3 | ORAI calcium release-activated calcium modulator 3 | A_23_P106898 | 2.18 |
| ZBTB47 | zinc finger and BTB domain containing 47 | A_24_P71700 | 2.18 |
| JDP2 | Jun dimerization protein 2 | A_23_P117582 | 2.17 |
| RAB7A | RAB7A, member RAS oncogene family | A_33_P3361851 | 2.17 |
| SNAI3-AS1 | SNAI3 antisense RNA 1 | A_23_P406227 | 2.16 |
| MFSD7 | major facilitator superfamily domain containing 7 | A_23_P72157 | 2.16 |
| GGACT | gamma-glutamylamine cyclotransferase | A_19_P00809029 | 2.15 |
| XLOC_l2_008691 |  | A_19_P00319311 | 2.15 |
| HES2 | hes family bHLH transcription factor 2 | A_23_P304716 | 2.15 |
| SUSD4 | sushi domain containing 4 | A_23_P201066 | 2.15 |
| ZNF706 | zinc finger protein 706 | A_19_P00328670 | 2.15 |
| MRPL43 | mitochondrial ribosomal protein L43 | A_23_P346384 | 2.15 |
| C1RL-AS1 | C1RL antisense RNA 1 | A_33_P3365408 | 2.14 |
| SRPR | signal recognition particle receptor (docking protein) | A_23_P86943 | 2.13 |
| C2orf68 | chromosome 2 open reading frame 68 | A_33_P3404959 | 2.13 |
| PLCB2 | phospholipase C, beta 2 | A_33_P3260614 | 2.12 |
| ACSS1 | acyl-CoA synthetase short-chain family member 1 | A_23_P120594 | 2.12 |
| FLJ42627 | uncharacterized LOC645644 | A_33_P3354796 | 2.11 |
| SEC24B-AS1 | SEC24B antisense RNA 1 | A_19_P00332068 | 2.11 |
| ALDH3B1 | aldehyde dehydrogenase 3 family, member B1 | A_24_P218688 | 2.11 |
| ACOX2 | acyl-CoA oxidase 2, branched chain | A_23_P10182 | 2.11 |
| SLCO4A1 | solute carrier organic anion transporter family, member 4A1 | A_23_P5903 | 2.10 |
| lnc-SPATC1-1 | lnc-SPATC1-1:2 | A_33_P3325758 | 2.10 |
| C14orf132 | chromosome 14 open reading frame 132 | A_19_P00807437 | 2.09 |
| FAM49B | family with sequence similarity 49, member B | A_23_P43255 | 2.09 |
| CCNJ | cyclin J | A_23_P24176 | 2.09 |
| DPRXP4 | divergent-paired related homeobox pseudogene 4 | A_33_P3306048 | 2.08 |
| LINGO1 | leucine rich repeat and Ig domain containing 1 | A_24_P112941 | 2.08 |
| VKORC1L1 | vitamin K epoxide reductase complex, subunit 1-like 1 | A_23_P413815 | 2.08 |
| SLC16A9 | solute carrier family 16, member 9 | A_23_P115726 | 2.08 |
| SP9 | Sp9 transcription factor | A_33_P3383184 | 2.08 |
| C20orf195 | chromosome 20 open reading frame 195 | A_33_P3405459 | 2.07 |
| NUDT3 | nudix (nucleoside diphosphate linked moiety X)-type motif 3 | A_19_P00806911 | 2.07 |
| TMEM117 | transmembrane protein 117 | A_23_P2573 | 2.06 |
| HCAR3 | hydroxycarboxylic acid receptor 3 | A_23_P64721 | 2.06 |
| BACE2 | beta-site APP-cleaving enzyme 2 | A_23_P154875 | 2.06 |
| MST1R | macrophage stimulating 1 receptor (c-met-related tyrosine kinase) | A_23_P256312 | 2.06 |
| APOBEC3C | apolipoprotein B mRNA editing enzyme, catalytic polypeptide-like 3C | A_23_P120931 | 2.05 |
| C2orf68 | chromosome 2 open reading frame 68 | A_32_P36046 | 2.05 |
| INHBA | inhibin, beta A | A_23_P122924 | 2.04 |
| CALML6 | calmodulin-like 6 | A_23_P62588 | 2.04 |
| DUSP2 | dual specificity phosphatase 2 | A_24_P37409 | 2.04 |
| RNF125 | ring finger protein 125, E3 ubiquitin protein ligase | A_33_P3257993 | 2.03 |
| YBX3 | Y box binding protein 3 | A_23_P25224 | 2.03 |
| CRLF1 | cytokine receptor-like factor 1 | A_33_P3252286 | 2.03 |
| VSTM2A-OT1 | VSTM2A overlapping transcript 1 | A_33_P3564487 | 2.02 |
| lnc-BAI1-1 | lnc-BAI1-1:1 | A_33_P3463355 | 2.02 |
| ARVCF | armadillo repeat gene deleted in velocardiofacial syndrome | A_33_P3236993 | 2.01 |
| ZNF469 | zinc finger protein 469 | A_32_P116556 | 2.01 |
| UBE2E3 | ubiquitin-conjugating enzyme E2E 3 | A_19_P00323103 | 2.01 |
| LOC100996579 | uncharacterized LOC100996579 | A_19_P00320434 | 2.01 |
| VAMP4 | vesicle-associated membrane protein 4 | A_23_P61569 | 2.00 |
| PPL | periplakin | A_23_P106906 | 2.00 |
| HSPB1 | heat shock 27kDa protein 1 | A_33_P3353242 | 2.00 |
| RFFL | ring finger and FYVE-like domain containing E3 ubiquitin protein ligase | A_23_P164089 | 2.00 |
| ARID3A | AT rich interactive domain 3A (BRIGHT-like) | A_33_P3272921 | 1.99 |
| LOC100130899 | uncharacterized LOC100130899 | A_33_P3240747 | 1.98 |
| ZBTB7A | zinc finger and BTB domain containing 7A | A_33_P3283780 | 1.98 |
| BMPR1A | bone morphogenetic protein receptor, type IA | A_19_P00803411 | 1.98 |
| RUNX1 | runt-related transcription factor 1 | A_24_P34155 | 1.98 |
| REG1A | regenerating islet-derived 1 alpha | A_23_P90743 | 1.98 |
| C14orf132 | chromosome 14 open reading frame 132 | A_19_P00332220 | 1.98 |
| FUBP1 | far upstream element (FUSE) binding protein 1 | A_23_P45699 | 1.98 |
| FBXO33 | F-box protein 33 | A_24_P41021 | 1.98 |
| OBFC1 | oligonucleotide/oligosaccharide-binding fold containing 1 | A_24_P759674 | 1.97 |
| RHBDF2 | rhomboid 5 homolog 2 (Drosophila) | A_23_P329870 | 1.97 |
| MRM1 | mitochondrial rRNA methyltransferase 1 homolog (S. cerevisiae) | A_23_P15603 | 1.97 |
| FBRSL1 | fibrosin-like 1 | A_23_P368934 | 1.97 |
| ZNF676 | zinc finger protein 676 | A_24_P205036 | 1.97 |
| HAS3 | hyaluronan synthase 3 | A_23_P393034 | 1.97 |
| DQX1 | DEAQ box RNA-dependent ATPase 1 | A_23_P56659 | 1.96 |
| CPT1B | carnitine palmitoyltransferase 1B (muscle) | A_23_P218817 | 1.96 |
| FUBP1 | far upstream element (FUSE) binding protein 1 | A_33_P3281151 | 1.96 |
| OMA1 | OMA1 zinc metallopeptidase | A_19_P00803169 | 1.96 |
| MTURN | maturin, neural progenitor differentiation regulator homolog (Xenopus) | A_32_P100439 | 1.95 |
| ZNF470 | zinc finger protein 470 | A_33_P3338036 | 1.95 |
| TGFBR2 | transforming growth factor, beta receptor II (70/80kDa) | A_33_P3313825 | 1.95 |
| APOBEC3F | apolipoprotein B mRNA editing enzyme, catalytic polypeptide-like 3F | A_23_P357101 | 1.95 |
| CAMTA1 | calmodulin binding transcription activator 1 | A_32_P4985 | 1.95 |
| PTAFR | platelet-activating factor receptor | A_24_P102821 | 1.94 |
| PRRG1 | proline rich Gla (G-carboxyglutamic acid) 1 | A_33_P3328637 | 1.94 |
| lnc-SIK1-2 | lnc-SIK1-2:1 | A_19_P00808865 | 1.94 |
| PTPN18 | protein tyrosine phosphatase, non-receptor type 18 (brain-derived) | A_24_P80135 | 1.94 |
| SEMA4G | sema domain, immunoglobulin domain (Ig), transmembrane domain (TM) and short cytoplasmic domain, (semaphorin) 4G | A_23_P127068 | 1.94 |
| PM20D2 | peptidase M20 domain containing 2 | A_32_P86118 | 1.93 |
| LOC100129275 | uncharacterized LOC100129275 | A_33_P3423590 | 1.93 |
| DHRS9 | dehydrogenase/reductase (SDR family) member 9 | A_23_P56559 | 1.93 |
| LOXL4 | lysyl oxidase-like 4 | A_24_P406754 | 1.93 |
| B3GALT6 | UDP-Gal:betaGal beta 1,3-galactosyltransferase polypeptide 6 | A_33_P3407042 | 1.92 |
| MMAB | methylmalonic aciduria (cobalamin deficiency) cblB type | A_33_P3367201 | 1.91 |
| MOB3A | MOB kinase activator 3A | A_23_P130919 | 1.91 |
| PI3 | peptidase inhibitor 3, skin-derived | A_23_P210465 | 1.91 |
| HAR1B | highly accelerated region 1B (non-protein coding) | A_33_P3267330 | 1.90 |
| PCDHGB7 | protocadherin gamma subfamily B, 7 | A_33_P3410981 | 1.90 |
| BAIAP3 | BAI1-associated protein 3 | A_23_P163492 | 1.90 |
| RNF44 | ring finger protein 44 | A_23_P213592 | 1.90 |
| D2HGDH | D-2-hydroxyglutarate dehydrogenase | A_23_P376627 | 1.90 |
| NFIX | nuclear factor I/X (CCAAT-binding transcription factor) | A_33_P3884230 | 1.89 |
| CFAP61 | cilia and flagella associated protein 61 | A_33_P3395783 | 1.89 |
| CBLC | Cbl proto-oncogene C, E3 ubiquitin protein ligase | A_23_P397910 | 1.89 |
| LOC389834 | ankyrin repeat domain 57 pseudogene | A_33_P3245449 | 1.88 |
| GAA | glucosidase, alpha; acid | A_23_P153026 | 1.88 |
| FBN3 | fibrillin 3 | A_33_P3300430 | 1.88 |
| RUFY2 | RUN and FYVE domain containing 2 | A_33_P3346132 | 1.88 |
| MPZL2 | myelin protein zero-like 2 | A_23_P150379 | 1.88 |
| RAB14 | RAB14, member RAS oncogene family | A_24_P115511 | 1.88 |
| TBC1D8 | TBC1 domain family, member 8 (with GRAM domain) | A_33_P3309075 | 1.88 |
| TMEM8B | transmembrane protein 8B | A_24_P317827 | 1.88 |
| PTP4A3 | protein tyrosine phosphatase type IVA, member 3 | A_33_P3315906 | 1.88 |
| MFSD3 | major facilitator superfamily domain containing 3 | A_23_P406350 | 1.87 |
| MARC1 | mitochondrial amidoxime reducing component 1 | A_33_P3293362 | 1.87 |
| MTG1 | mitochondrial ribosome-associated GTPase 1 | A_33_P3380263 | 1.87 |
| GSTA4 | glutathione S-transferase alpha 4 | A_33_P3257903 | 1.87 |
| DVL3 | dishevelled segment polarity protein 3 | A_24_P291588 | 1.87 |
| BIK | BCL2-interacting killer (apoptosis-inducing) | A_23_P404667 | 1.87 |
| EPHA4 | EPH receptor A4 | A_23_P108501 | 1.87 |
| WBSCR27 | Williams Beuren syndrome chromosome region 27 | A_23_P381017 | 1.87 |
| LEF1 | lymphoid enhancer-binding factor 1 | A_24_P20630 | 1.86 |
| C3orf52 | chromosome 3 open reading frame 52 | A_23_P58009 | 1.86 |
| SESN2 | sestrin 2 | A_23_P35082 | 1.86 |
| ADAM11 | ADAM metallopeptidase domain 11 | A_23_P207345 | 1.86 |
| MAP3K14-AS1 | MAP3K14 antisense RNA 1 | A_33_P3231858 | 1.86 |
| KDM4B | lysine (K)-specific demethylase 4B | A_33_P3319920 | 1.86 |
| BCL2L11 | BCL2-like 11 (apoptosis facilitator) | A_24_P122921 | 1.85 |
| ZHX1-C8orf76 | ZHX1-C8orf76 readthrough | A_33_P3373214 | 1.85 |
| PRPF40B | PRP40 pre-mRNA processing factor 40 homolog B (S. cerevisiae) | A_23_P411922 | 1.85 |
| PROSER1 | proline and serine rich 1 | A_33_P3398533 | 1.84 |
| IFI44L | interferon-induced protein 44-like | A_23_P45871 | 1.84 |
| CTAGE5 | CTAGE family, member 5 | A_32_P116840 | 1.84 |
| G3BP1 | GTPase activating protein (SH3 domain) binding protein 1 | A_19_P00325917 | 1.84 |
| SLC20A2 | solute carrier family 20 (phosphate transporter), member 2 | A_23_P94921 | 1.84 |
| ZNF592 | zinc finger protein 592 | A_33_P3418726 | 1.84 |
| SAMD5 | sterile alpha motif domain containing 5 | A_24_P602871 | 1.84 |
| SYPL1 | synaptophysin-like 1 | A_23_P93881 | 1.84 |
| ACY1 | aminoacylase 1 | A_23_P57868 | 1.83 |
| FBXO27 | F-box protein 27 | A_24_P113264 | 1.83 |
| NRARP | NOTCH-regulated ankyrin repeat protein | A_33_P3337272 | 1.83 |
| SLC29A3 | solute carrier family 29 (equilibrative nucleoside transporter), member 3 | A_23_P46871 | 1.83 |
| EIF5A2 | eukaryotic translation initiation factor 5A2 | A_24_P380022 | 1.83 |
| COMTD1 | catechol-O-methyltransferase domain containing 1 | A_33_P3257222 | 1.83 |
| C1QTNF6 | C1q and tumor necrosis factor related protein 6 | A_24_P211565 | 1.83 |
| TMEM198B | transmembrane protein 198B, pseudogene | A_33_P3259557 | 1.83 |
| AACSP1 | acetoacetyl-CoA synthetase pseudogene 1 | A_33_P3302290 | 1.82 |
| GRM4 | glutamate receptor, metabotropic 4 | A_33_P3282390 | 1.82 |
| RHOBTB1 | Rho-related BTB domain containing 1 | A_33_P3348288 | 1.82 |
| GSTA4 | glutathione S-transferase alpha 4 | A_23_P110941 | 1.82 |
| IL17RD | interleukin 17 receptor D | A_32_P188860 | 1.82 |
| NLRP1 | NLR family, pyrin domain containing 1 | A_23_P89550 | 1.82 |
| ADA | adenosine deaminase | A_23_P210482 | 1.82 |
| SPATA13 | spermatogenesis associated 13 | A_33_P3298111 | 1.81 |
| SDC3 | syndecan 3 | A_23_P74887 | 1.81 |
| HSD17B8 | hydroxysteroid (17-beta) dehydrogenase 8 | A_23_P81973 | 1.81 |
| FJX1 | four jointed box 1 (Drosophila) | A_23_P150693 | 1.81 |
| PRKX | protein kinase, X-linked | A_23_P217339 | 1.81 |
| GXYLT1P3 | glucoside xylosyltransferase 1 pseudogene 3 | A_33_P3283848 | 1.81 |
| BCL2L11 | BCL2-like 11 (apoptosis facilitator) | A_33_P3398526 | 1.80 |
| DUSP5P1 | dual specificity phosphatase 5 pseudogene 1 | A_24_P367602 | 1.80 |
| EPHX2 | epoxide hydrolase 2, cytoplasmic | A_23_P8834 | 1.80 |
| MGAT4A | mannosyl (alpha-1,3-)-glycoprotein beta-1,4-N-acetylglucosaminyltransferase, isozyme A | A_23_P28507 | 1.80 |
| ST6GAL1 | ST6 beta-galactosamide alpha-2,6-sialyltranferase 1 | A_24_P388528 | 1.80 |
| SEPW1 | selenoprotein W, 1 | A_23_P27724 | 1.80 |
| ZC3H18-AS1 | ZC3H18 antisense RNA 1 (head to head) | A_33_P3398111 | 1.80 |
| PNPLA3 | patatin-like phospholipase domain containing 3 | A_23_P17914 | 1.80 |
| OR6C76 | olfactory receptor, family 6, subfamily C, member 76 | A_33_P3390327 | 1.80 |
| GRAMD4 | GRAM domain containing 4 | A_24_P23258 | 1.79 |
| RNF122 | ring finger protein 122 | A_23_P134744 | 1.79 |
| ARID3B | AT rich interactive domain 3B (BRIGHT-like) | A_23_P88580 | 1.79 |
| CIRBP-AS1 | CIRBP antisense RNA 1 | A_33_P3851788 | 1.79 |
| LOC728743 | zinc finger protein pseudogene | A_33_P3323188 | 1.79 |
| FCGBP | Fc fragment of IgG binding protein | A_23_P21495 | 1.79 |
| GABRD | gamma-aminobutyric acid (GABA) A receptor, delta | A_23_P309720 | 1.78 |
| ZDHHC8 | zinc finger, DHHC-type containing 8 | A_33_P3238171 | 1.78 |
| TRIM29 | tripartite motif containing 29 | A_23_P203267 | 1.78 |
| SPOPL | speckle-type POZ protein-like | A_24_P551028 | 1.78 |
| EMR2 | egf-like module containing, mucin-like, hormone receptor-like 2 | A_23_P502336 | 1.78 |
| OR10G8 | olfactory receptor, family 10, subfamily G, member 8 | A_23_P125204 | 1.78 |
| UBE2E3 | ubiquitin-conjugating enzyme E2E 3 | A_19_P00326822 | 1.78 |
| GYLTL1B | glycosyltransferase-like 1B | A_23_P104617 | 1.77 |
| C3orf17 | chromosome 3 open reading frame 17 | A_24_P921155 | 1.77 |
| MAPKAP1 | mitogen-activated protein kinase associated protein 1 | A_23_P216894 | 1.77 |
| BAIAP2 | BAI1-associated protein 2 | A_23_P315836 | 1.77 |
| SLPI | secretory leukocyte peptidase inhibitor | A_24_P190472 | 1.76 |
| CHMP3 | charged multivesicular body protein 3 | A_24_P945293 | 1.76 |
| PRKY | protein kinase, Y-linked, pseudogene | A_23_P137248 | 1.76 |
| RNF135 | ring finger protein 135 | A_23_P252283 | 1.76 |
| NBEAL2 | neurobeachin-like 2 | A_23_P409168 | 1.76 |
| GUSB | glucuronidase, beta | A_23_P334608 | 1.76 |
| STK3 | serine/threonine kinase 3 | A_33_P3260669 | 1.75 |
| PLAG1 | pleiomorphic adenoma gene 1 | A_23_P411723 | 1.75 |
| C15orf39 | chromosome 15 open reading frame 39 | A_23_P37514 | 1.75 |
| MOGAT2 | monoacylglycerol O-acyltransferase 2 | A_33_P3353866 | 1.75 |
| UCN | urocortin | A_33_P3353030 | 1.75 |
| CYP4Z1 | cytochrome P450, family 4, subfamily Z, polypeptide 1 | A_33_P3279880 | 1.75 |
| CCDC88C | coiled-coil domain containing 88C | A_23_P379945 | 1.75 |
| PEX6 | peroxisomal biogenesis factor 6 | A_33_P3265016 | 1.75 |
| KAZN | kazrin, periplakin interacting protein | A_24_P331830 | 1.74 |
| NUDT3 | nudix (nucleoside diphosphate linked moiety X)-type motif 3 | A_19_P00802759 | 1.74 |
| PRDM16 | PR domain containing 16 | A_32_P225816 | 1.74 |
| AP5B1 | adaptor-related protein complex 5, beta 1 subunit | A_24_P9883 | 1.74 |
| GRB10 | growth factor receptor-bound protein 10 | A_24_P235266 | 1.74 |
| SLC27A3 | solute carrier family 27 (fatty acid transporter), member 3 | A_24_P179816 | 1.74 |
| C22orf29 | chromosome 22 open reading frame 29 | A_33_P3297205 | 1.74 |
| ANO7 | anoctamin 7 | A_23_P335495 | 1.74 |
| SLC48A1 | solute carrier family 48 (heme transporter), member 1 | A_24_P309594 | 1.74 |
| LOC100131564 | uncharacterized LOC100131564 | A_24_P481375 | 1.74 |
| ALS2CL | ALS2 C-terminal like | A_32_P420009 | 1.73 |
| ZDHHC11 | zinc finger, DHHC-type containing 11 | A_33_P3344204 | 1.73 |
| CDHR3 | cadherin-related family member 3 | A_19_P00320588 | 1.73 |
| GYLTL1B | glycosyltransferase-like 1B | A_33_P3390539 | 1.73 |
| SNN | stannin | A_24_P30923 | 1.73 |
| PEX6 | peroxisomal biogenesis factor 6 | A_23_P42144 | 1.73 |
| XYLT2 | xylosyltransferase II | A_23_P15582 | 1.73 |
| DNMT3B | DNA (cytosine-5-)-methyltransferase 3 beta | A_23_P28953 | 1.73 |
| ESPN | espin | A_23_P12405 | 1.73 |
| IGF1R | insulin-like growth factor 1 receptor | A_23_P417282 | 1.73 |
| CEMIP | cell migration inducing protein, hyaluronan binding | A_23_P324754 | 1.72 |
| TTLL10-AS1 | TTLL10 antisense RNA 1 | A_33_P3352048 | 1.72 |
| PLEKHJ1 | pleckstrin homology domain containing, family J member 1 | A_24_P202717 | 1.72 |
| ALG13 | ALG13, UDP-N-acetylglucosaminyltransferase subunit | A_23_P11279 | 1.72 |
| LOC646626 | uncharacterized LOC646626 | A_32_P703 | 1.72 |
| TP53I11 | tumor protein p53 inducible protein 11 | A_23_P150281 | 1.72 |
| NAPRT | nicotinate phosphoribosyltransferase | A_23_P43238 | 1.72 |
| PRKXP1 | protein kinase, X-linked, pseudogene 1 | A_33_P3618429 | 1.72 |
| SLC9B2 | solute carrier family 9, subfamily B (NHA2, cation proton antiporter 2), member 2 | A_23_P341567 | 1.71 |
| WDR18 | WD repeat domain 18 | A_33_P3411925 | 1.71 |
| PLEKHA5 | pleckstrin homology domain containing, family A member 5 | A_33_P3328772 | 1.71 |
| POLR3D | polymerase (RNA) III (DNA directed) polypeptide D, 44kDa | A_23_P84565 | 1.70 |
| NADK | NAD kinase | A_23_P200056 | 1.70 |
| SH2D2A | SH2 domain containing 2A | A_23_P160618 | 1.70 |
| PVT1 | Pvt1 oncogene (non-protein coding) | A_19_P00317871 | 1.70 |
| ZNF814 | zinc finger protein 814 | A_33_P3228757 | 1.70 |
| TMEM198B | transmembrane protein 198B, pseudogene | A_24_P93703 | 1.70 |
| LOC101927910 | keratin-associated protein 5-5-like | A_23_P54692 | 1.70 |
| ZNF852 | zinc finger protein 852 | A_33_P3552465 | 1.70 |
| EXTL3 | exostosin-like glycosyltransferase 3 | A_33_P3291294 | 1.69 |
| HS2ST1 | heparan sulfate 2-O-sulfotransferase 1 | A_33_P3269069 | 1.69 |
| PTPRU | protein tyrosine phosphatase, receptor type, U | A_33_P3309491 | 1.69 |
| PRAMEF11 | PRAME family member 11 | A_33_P3303594 | 1.69 |
| PLIN5 | perilipin 5 | A_23_P39251 | 1.69 |
| NSG1 | neuron specific gene family member 1 | A_33_P3259135 | 1.69 |
| IL15RA | interleukin 15 receptor, alpha | A_23_P138680 | 1.69 |
| CDC42EP1 | CDC42 effector protein (Rho GTPase binding) 1 | A_33_P3407424 | 1.68 |
| ABCC2 | ATP-binding cassette, sub-family C (CFTR/MRP), member 2 | A_23_P44569 | 1.68 |
| NLK | nemo-like kinase | A_33_P3327108 | 1.68 |
| CFD | complement factor D (adipsin) | A_23_P119562 | 1.68 |
| lnc-PLEKHH2-1 | lnc-PLEKHH2-1:1 | A_33_P3421611 | 1.68 |
| SON | SON DNA binding protein | A_33_P3421748 | 1.68 |
| FERMT1 | fermitin family member 1 | A_23_P131935 | 1.67 |
| MAP4K4 | mitogen-activated protein kinase kinase kinase kinase 4 | A_23_P90804 | 1.67 |
| FABP6 | fatty acid binding protein 6, ileal | A_23_P43846 | 1.67 |
| SEPW1 | selenoprotein W, 1 | A_33_P3326432 | 1.67 |
| C5orf60 | chromosome 5 open reading frame 60 | A_33_P3283592 | 1.67 |
| BAI1 | brain-specific angiogenesis inhibitor 1 | A_33_P3285470 | 1.67 |
| ZNF497 | zinc finger protein 497 | A_33_P3323323 | 1.67 |
| ERVMER34-1 | endogenous retrovirus group MER34, member 1 | A_19_P00319407 | 1.67 |
| OSBPL5 | oxysterol binding protein-like 5 | A_23_P53081 | 1.66 |
| PRKAA1 | protein kinase, AMP-activated, alpha 1 catalytic subunit | A_33_P3257187 | 1.66 |
| TCEANC2 | transcription elongation factor A (SII) N-terminal and central domain containing 2 | A_23_P348281 | 1.66 |
| SLC27A1 | solute carrier family 27 (fatty acid transporter), member 1 | A_24_P382489 | 1.66 |
| LYRM9 | LYR motif containing 9 | A_23_P392126 | 1.66 |
| C22orf46 | chromosome 22 open reading frame 46 | A_23_P132341 | 1.66 |
| RETSAT | retinol saturase (all-trans-retinol 13,14-reductase) | A_23_P209944 | 1.66 |
| ECHDC3 | enoyl CoA hydratase domain containing 3 | A_24_P343621 | 1.66 |
| IDS | iduronate 2-sulfatase | A_23_P217475 | 1.65 |
| ICA1 | islet cell autoantigen 1, 69kDa | A_23_P215419 | 1.65 |
| FOXN3-AS1 | FOXN3 antisense RNA 1 | A_24_P306720 | 1.65 |
| USP53 | ubiquitin specific peptidase 53 | A_24_P137522 | 1.65 |
| HSPG2 | heparan sulfate proteoglycan 2 | A_33_P3321657 | 1.65 |
| EXD3 | exonuclease 3'-5' domain containing 3 | A_33_P3319261 | 1.65 |
| JAK3 | Janus kinase 3 | A_24_P59667 | 1.65 |
| CLEC16A | C-type lectin domain family 16, member A | A_33_P3422888 | 1.65 |
| PLEKHA2 | pleckstrin homology domain containing, family A (phosphoinositide binding specific) member 2 | A_33_P3339860 | 1.65 |
| ZMYM6NB | ZMYM6 neighbor | A_24_P370670 | 1.65 |
| MARC1 | mitochondrial amidoxime reducing component 1 | A_33_P3247205 | 1.65 |
| TMC4 | transmembrane channel-like 4 | A_23_P330461 | 1.65 |
| TRIM14 | tripartite motif containing 14 | A_23_P216655 | 1.65 |
| TRIB2 | tribbles pseudokinase 2 | A_24_P396753 | 1.65 |
| LOC100131514 | mucin-3A-like | A_33_P3371237 | 1.64 |
| SMIM20 | small integral membrane protein 20 | A_24_P341222 | 1.64 |
| BTG2 | BTG family, member 2 | A_23_P62901 | 1.64 |
| LRRC8A | leucine rich repeat containing 8 family, member A | A_23_P250274 | 1.64 |
| MXD1 | MAX dimerization protein 1 | A_24_P379750 | 1.64 |
| VPS26A | vacuolar protein sorting 26 homolog A (S. pombe) | A_23_P75255 | 1.64 |
| CREM | cAMP responsive element modulator | A_24_P360763 | 1.64 |
| AUTS2 | autism susceptibility candidate 2 | A_23_P122906 | 1.64 |
| DOCK6 | dedicator of cytokinesis 6 | A_23_P67299 | 1.63 |
| CDK16 | cyclin-dependent kinase 16 | A_33_P3341239 | 1.63 |
| PIGQ | phosphatidylinositol glycan anchor biosynthesis, class Q | A_23_P502609 | 1.63 |
| FLJ26086 | uncharacterized LOC440129 | A_33_P3555368 | 1.63 |
| PPP3CA | protein phosphatase 3, catalytic subunit, alpha isozyme | A_24_P414371 | 1.63 |
| SOAT2 | sterol O-acyltransferase 2 | A_23_P25475 | 1.63 |
| SEC14L2 | SEC14-like 2 (S. cerevisiae) | A_23_P17811 | 1.63 |
| SLC37A3 | solute carrier family 37, member 3 | A_23_P95130 | 1.63 |
| CEBPA | CCAAT/enhancer binding protein (C/EBP), alpha | A_24_P224727 | 1.63 |
| REG3A | regenerating islet-derived 3 alpha | A_23_P119936 | 1.63 |
| WDR81 | WD repeat domain 81 | A_23_P354208 | 1.62 |
| FOXL1 | forkhead box L1 | A_33_P3355503 | 1.62 |
| PRDM2 | PR domain containing 2, with ZNF domain | A_33_P3319331 | 1.62 |
| HERC6 | HECT and RLD domain containing E3 ubiquitin protein ligase family member 6 | A_33_P3315779 | 1.62 |
| ALKBH5 | AlkB family member 5, RNA demethylase | A_23_P153050 | 1.62 |
| LINC01144 | long intergenic non-protein coding RNA 1144 | A_33_P3239317 | 1.62 |
| EPX | eosinophil peroxidase | A_33_P3222253 | 1.62 |
| B3GNT3 | UDP-GlcNAc:betaGal beta-1,3-N-acetylglucosaminyltransferase 3 | A_23_P78980 | 1.62 |
| PLXND1 | plexin D1 | A_24_P376391 | 1.62 |
| ERVMER34-1 | endogenous retrovirus group MER34, member 1 | A_19_P00319409 | 1.62 |
| CECR2 | cat eye syndrome chromosome region, candidate 2 | A_23_P211326 | 1.62 |
| ANKMY1 | ankyrin repeat and MYND domain containing 1 | A_33_P3315554 | 1.62 |
| RAB22A | RAB22A, member RAS oncogene family | A_33_P3221788 | 1.62 |
| TRIM14 | tripartite motif containing 14 | A_24_P197964 | 1.62 |
| GALNT12 | polypeptide N-acetylgalactosaminyltransferase 12 | A_23_P415652 | 1.62 |
| CD1B | CD1b molecule | A_23_P351844 | 1.62 |
| HOXC4 | homeobox C4 | A_33_P3300975 | 1.62 |
| RTN4RL1 | reticulon 4 receptor-like 1 | A_23_P66481 | 1.62 |
| TTC25 | tetratricopeptide repeat domain 25 | A_23_P73150 | 1.62 |
| ZNF385A | zinc finger protein 385A | A_24_P403734 | 1.62 |
| ZNF485 | zinc finger protein 485 | A_23_P115861 | 1.61 |
| LMNTD2 | lamin tail domain containing 2 | A_33_P3377005 | 1.61 |
| ANTXR1 | anthrax toxin receptor 1 | A_23_P84576 | 1.61 |
| UBE2D1 | ubiquitin-conjugating enzyme E2D 1 | A_24_P13032 | 1.61 |
| METTL21B | methyltransferase like 21B | A_23_P320878 | 1.61 |
| BREA2 | breast cancer estrogen-induced apoptosis 2 | A_33_P3260500 | 1.61 |
| CTC1 | CTS telomere maintenance complex component 1 | A_33_P3312104 | 1.61 |
| WIPF3 | WAS/WASL interacting protein family, member 3 | A_32_P3914 | 1.61 |
| GUSBP11 | glucuronidase, beta pseudogene 11 | A_33_P3374559 | 1.61 |
| C1orf110 | chromosome 1 open reading frame 110 | A_24_P6370 | 1.61 |
| AK3 | adenylate kinase 3 | A_33_P3263061 | 1.61 |
| P3H4 | prolyl 3-hydroxylase family member 4 (non-enzymatic) | A_33_P3257030 | 1.61 |
| SUSD3 | sushi domain containing 3 | A_23_P401076 | 1.60 |
| ANKRD13B | ankyrin repeat domain 13B | A_23_P380881 | 1.60 |
| TMC6 | transmembrane channel-like 6 | A_23_P101013 | 1.60 |
| FAM188B | family with sequence similarity 188, member B | A_33_P3326713 | 1.60 |
| CROCC | ciliary rootlet coiled-coil, rootletin | A_33_P3663705 | 1.60 |
| TRIM14 | tripartite motif containing 14 | A_23_P425752 | 1.60 |
| NAGLU | N-acetylglucosaminidase, alpha | A_23_P26945 | 1.60 |
| TERT | telomerase reverse transcriptase | A_23_P110851 | 1.60 |
| CYB5D2 | cytochrome b5 domain containing 2 | A_24_P228026 | 1.60 |
| GPR114 | G protein-coupled receptor 114 | A_23_P206293 | 1.60 |
| FBXO17 | F-box protein 17 | A_23_P101871 | 1.60 |
| GJA1 | gap junction protein, alpha 1, 43kDa | A_24_P55295 | 1.60 |
| PARVB | parvin, beta | A_23_P40718 | 1.60 |
| DND1 | DND microRNA-mediated repression inhibitor 1 | A_33_P3224723 | 1.60 |
| GP1BB | glycoprotein Ib (platelet), beta polypeptide | A_23_P29124 | 1.60 |
| CEACAM3 | carcinoembryonic antigen-related cell adhesion molecule 3 | A_23_P130515 | 1.60 |
| ZSWIM4 | zinc finger, SWIM-type containing 4 | A_33_P3233150 | 1.60 |
| ASL | argininosuccinate lyase | A_23_P26223 | 1.60 |
| AMH | anti-Mullerian hormone | A_23_P78944 | 1.60 |
| ONECUT1 | one cut homeobox 1 | A_23_P340717 | 1.60 |
| ITPRIP | inositol 1,4,5-trisphosphate receptor interacting protein | A_23_P340333 | 1.60 |
| IDUA | iduronidase, alpha-L- | A_33_P3392087 | 1.60 |
| LRRC1 | leucine rich repeat containing 1 | A_23_P215024 | 1.59 |
| ZFP36L1 | ZFP36 ring finger protein-like 1 | A_23_P99540 | 1.59 |
| THEM6 | thioesterase superfamily member 6 | A_33_P3249748 | 1.59 |
| CHD6 | chromodomain helicase DNA binding protein 6 | A_23_P102607 | 1.59 |
| PTPN18 | protein tyrosine phosphatase, non-receptor type 18 (brain-derived) | A_23_P210015 | 1.59 |
| LY9 | lymphocyte antigen 9 | A_24_P324674 | 1.59 |
| ACTR1B | ARP1 actin-related protein 1 homolog B, centractin beta (yeast) | A_23_P28279 | 1.59 |
| CST3 | cystatin C | A_33_P3228271 | 1.59 |
| ZNF655 | zinc finger protein 655 | A_23_P215819 | 1.59 |
| RNASE3 | ribonuclease, RNase A family, 3 | A_23_P163025 | 1.59 |
| lnc-RPL7L1-1 | lnc-RPL7L1-1:1 | A_19_P00326821 | 1.59 |
| MAL2 | mal, T-cell differentiation protein 2 (gene/pseudogene) | A_23_P60130 | 1.59 |
| BMP6 | bone morphogenetic protein 6 | A_23_P19624 | 1.58 |
| RNF216P1 | ring finger protein 216 pseudogene 1 | A_24_P598406 | 1.58 |
| MYO1F | myosin IF | A_23_P142447 | 1.58 |
| SEMA3F | sema domain, immunoglobulin domain (Ig), short basic domain, secreted, (semaphorin) 3F | A_33_P3813128 | 1.58 |
| RREB1 | ras responsive element binding protein 1 | A_33_P3220728 | 1.58 |
| CD2AP | CD2-associated protein | A_32_P155811 | 1.58 |
| ZNF589 | zinc finger protein 589 | A_24_P247978 | 1.58 |
| lnc-TMED5-1 | lnc-TMED5-1:28 | A_33_P3422289 | 1.58 |
| KIAA0922 | KIAA0922 | A_33_P3220723 | 1.57 |
| RALGDS | ral guanine nucleotide dissociation stimulator | A_23_P135184 | 1.57 |
| CLMN | calmin (calponin-like, transmembrane) | A_32_P167471 | 1.57 |
| NUP54 | nucleoporin 54kDa | A_23_P92320 | 1.57 |
| FBXW4 | F-box and WD repeat domain containing 4 | A_23_P342825 | 1.57 |
| FAM198A | family with sequence similarity 198, member A | A_33_P3351316 | 1.57 |
| TMEM243 | transmembrane protein 243, mitochondrial | A_23_P157283 | 1.57 |
| KCNIP2-AS1 | KCNIP2 antisense RNA 1 | A_33_P3412658 | 1.57 |
| CELSR3 | cadherin, EGF LAG seven-pass G-type receptor 3 | A_23_P92093 | 1.57 |
| ZNF503 | zinc finger protein 503 | A_33_P3268695 | 1.57 |
| RNPEPL1 | arginyl aminopeptidase (aminopeptidase B)-like 1 | A_33_P3322654 | 1.57 |
| LOC642852 | uncharacterized LOC642852 | A_32_P122940 | 1.57 |
| LTBP1 | latent transforming growth factor beta binding protein 1 | A_23_P43810 | 1.57 |
| HSD11B2 | hydroxysteroid (11-beta) dehydrogenase 2 | A_23_P14986 | 1.57 |
| NAB1 | NGFI-A binding protein 1 (EGR1 binding protein 1) | A_23_P209805 | 1.57 |
| LINC-PINT | long intergenic non-protein coding RNA, p53 induced transcript | A_19_P00318715 | 1.57 |
| ARIH2 | ariadne RBR E3 ubiquitin protein ligase 2 | A_24_P94651 | 1.56 |
| ZNF551 | zinc finger protein 551 | A_24_P68019 | 1.56 |
| ZNRF1 | zinc and ring finger 1, E3 ubiquitin protein ligase | A_24_P16610 | 1.56 |
| GPR20 | G protein-coupled receptor 20 | A_23_P159237 | 1.56 |
| OR10P1 | olfactory receptor, family 10, subfamily P, member 1 | A_24_P167377 | 1.56 |
| UNKL | unkempt family zinc finger-like | A_23_P100141 | 1.56 |
| TLR3 | toll-like receptor 3 | A_23_P29922 | 1.56 |
| PTPN21 | protein tyrosine phosphatase, non-receptor type 21 | A_33_P3235990 | 1.56 |
| PLA2G12A | phospholipase A2, group XIIA | A_23_P30020 | 1.56 |
| ATP2A3 | ATPase, Ca++ transporting, ubiquitous | A_23_P207632 | 1.56 |
| ATG7 | autophagy related 7 | A_24_P944827 | 1.56 |
| EMID1 | EMI domain containing 1 | A_24_P14634 | 1.56 |
| CIAO1 | cytosolic iron-sulfur assembly component 1 | A_33_P3306397 | 1.56 |
| ONECUT2 | one cut homeobox 2 | A_32_P124708 | 1.56 |
| SYTL1 | synaptotagmin-like 1 | A_33_P3266744 | 1.56 |
| PACSIN3 | protein kinase C and casein kinase substrate in neurons 3 | A_24_P381136 | 1.56 |
| ACOXL | acyl-CoA oxidase-like | A_33_P3395008 | 1.56 |
| TBL1X | transducin (beta)-like 1X-linked | A_33_P3347161 | 1.56 |
| SLC46A1 | solute carrier family 46 (folate transporter), member 1 | A_33_P3235611 | 1.56 |
| lnc-ACOT12-2 | lnc-ACOT12-2:1 | A_33_P3300027 | 1.55 |
| WWP2 | WW domain containing E3 ubiquitin protein ligase 2 | A_24_P342086 | 1.55 |
| CTSC | cathepsin C | A_33_P3283480 | 1.55 |
| TNNC2 | troponin C type 2 (fast) | A_23_P131825 | 1.55 |
| MRPL19 | mitochondrial ribosomal protein L19 | A_33_P3223088 | 1.55 |
| BLZF1 | basic leucine zipper nuclear factor 1 | A_23_P23266 | 1.55 |
| KIAA1147 | KIAA1147 | A_33_P3421626 | 1.55 |
| GP1BB | glycoprotein Ib (platelet), beta polypeptide | A_33_P3265030 | 1.55 |
| MCF2 | MCF.2 cell line derived transforming sequence | A_23_P45536 | 1.55 |
| TMX4 | thioredoxin-related transmembrane protein 4 | A_24_P250535 | 1.55 |
| KIAA1033 | KIAA1033 | A_32_P153725 | 1.55 |
| NTNG1 | netrin G1 | A_24_P359671 | 1.55 |
| MARVELD1 | MARVEL domain containing 1 | A_23_P138725 | 1.54 |
| ICA1 | islet cell autoantigen 1, 69kDa | A_24_P372012 | 1.54 |
| TUB | tubby bipartite transcription factor | A_23_P500892 | 1.54 |
| SETD7 | SET domain containing (lysine methyltransferase) 7 | A_24_P251841 | 1.54 |
| NAAA | N-acylethanolamine acid amidase | A_23_P155666 | 1.54 |
| ZNF687 | zinc finger protein 687 | A_23_P331813 | 1.54 |
| KHNYN | KH and NYN domain containing | A_24_P170763 | 1.54 |
| YTHDF3 | YTH N(6)-methyladenosine RNA binding protein 3 | A_33_P3264419 | 1.54 |
| ATG9A | autophagy related 9A | A_33_P3270404 | 1.54 |
| EEF2K | eukaryotic elongation factor 2 kinase | A_33_P3342410 | 1.54 |
| TSPYL5 | TSPY-like 5 | A_23_P417951 | 1.54 |
| NCF1B | neutrophil cytosolic factor 1B pseudogene | A_33_P3224800 | 1.54 |
| ERC1 | ELKS/RAB6-interacting/CAST family member 1 | A_24_P162293 | 1.54 |
| CCDC64B | coiled-coil domain containing 64B | A_33_P3335590 | 1.53 |
| TOMM34 | translocase of outer mitochondrial membrane 34 | A_33_P3321711 | 1.53 |
| NLRX1 | NLR family member X1 | A_24_P244356 | 1.53 |
| EPPK1 | epiplakin 1 | A_24_P357169 | 1.53 |
| NBPF11 | neuroblastoma breakpoint family, member 11 | A_32_P149492 | 1.53 |
| RNF123 | ring finger protein 123 | A_23_P250054 | 1.53 |
| RNASET2 | ribonuclease T2 | A_33_P3254121 | 1.53 |
| ACP5 | acid phosphatase 5, tartrate resistant | A_23_P142075 | 1.53 |
| FDXR | ferredoxin reductase | A_23_P38154 | 1.53 |
| DICER1 | dicer 1, ribonuclease type III | A_33_P3212490 | 1.53 |
| EPHB6 | EPH receptor B6 | A_23_P145935 | 1.53 |
| GXYLT1 | glucoside xylosyltransferase 1 | A_23_P336796 | 1.53 |
| NDRG1 | N-myc downstream regulated 1 | A_23_P20494 | 1.53 |
| RPAP1 | RNA polymerase II associated protein 1 | A_23_P3453 | 1.53 |
| LOC100270804 | uncharacterized LOC100270804 | A_33_P3377239 | 1.53 |
| DDX6 | DEAD (Asp-Glu-Ala-Asp) box helicase 6 | A_24_P215407 | 1.53 |
| FUK | fucokinase | A_23_P429461 | 1.53 |
| SHISA5 | shisa family member 5 | A_24_P394246 | 1.53 |
| SPTLC2 | serine palmitoyltransferase, long chain base subunit 2 | A_24_P150486 | 1.53 |
| SBSN | suprabasin | A_33_P3269636 | 1.52 |
| MXI1 | MAX interactor 1, dimerization protein | A_23_P161399 | 1.52 |
| TANK | TRAF family member-associated NFKB activator | A_24_P257108 | 1.52 |
| SMIM1 | small integral membrane protein 1 (Vel blood group) | A_33_P3394868 | 1.52 |
| MREG | melanoregulin | A_23_P108948 | 1.52 |
| ATG9A | autophagy related 9A | A_33_P3226643 | 1.52 |
| DNAJB9 | DnaJ (Hsp40) homolog, subfamily B, member 9 | A_23_P258944 | 1.52 |
| XKR8 | XK, Kell blood group complex subunit-related family, member 8 | A_23_P149496 | 1.52 |
| IDH2 | isocitrate dehydrogenase 2 (NADP+), mitochondrial | A_23_P129209 | 1.52 |
| ZBED6CL | ZBED6 C-terminal like | A_23_P316460 | 1.52 |
| CYTH3 | cytohesin 3 | A_33_P3271051 | 1.52 |
| CHADL | chondroadherin-like | A_33_P3339531 | 1.52 |
| EZH1 | enhancer of zeste 1 polycomb repressive complex 2 subunit | A_23_P428219 | 1.52 |
| AIM2 | absent in melanoma 2 | A_32_P44394 | 1.51 |
| TRIM65 | tripartite motif containing 65 | A_33_P3331346 | 1.51 |
| HGH1 | HGH1 homolog (S. cerevisiae) | A_23_P61268 | 1.51 |
| HDAC1 | histone deacetylase 1 | A_23_P114656 | 1.51 |
| TRPM4 | transient receptor potential cation channel, subfamily M, member 4 | A_23_P153529 | 1.51 |
| KLHL7 | kelch-like family member 7 | A_23_P215517 | 1.51 |
| RNF216 | ring finger protein 216 | A_33_P3329878 | 1.51 |
| PRR5 | proline rich 5 (renal) | A_23_P80382 | 1.51 |
| GALNT11 | polypeptide N-acetylgalactosaminyltransferase 11 | A_23_P8416 | 1.51 |
| LOC102723908 | uncharacterized LOC102723908 | A_19_P00807678 | 1.51 |
| LOC102723908 | uncharacterized LOC102723908 | A_19_P00810604 | 1.51 |
| C16orf74 | chromosome 16 open reading frame 74 | A_24_P289178 | 1.51 |
| IKZF1 | IKAROS family zinc finger 1 (Ikaros) | A_33_P3842556 | 1.51 |
| NHSL1 | NHS-like 1 | A_23_P387184 | 1.51 |
| FBXL20 | F-box and leucine-rich repeat protein 20 | A_33_P3333587 | 1.51 |
| POLR3G | polymerase (RNA) III (DNA directed) polypeptide G (32kD) | A_33_P3396527 | 1.51 |
| STK10 | serine/threonine kinase 10 | A_33_P3389153 | 1.51 |
| GJB5 | gap junction protein, beta 5, 31.1kDa | A_23_P35293 | 1.50 |
| KIF26A | kinesin family member 26A | A_32_P27917 | 1.50 |
| SULT4A1 | sulfotransferase family 4A, member 1 | A_33_P3353345 | 1.50 |
| PIN4 | protein (peptidylprolyl cis/trans isomerase) NIMA-interacting, 4 (parvulin) | A_23_P315345 | 1.50 |
| CYP2R1 | cytochrome P450, family 2, subfamily R, polypeptide 1 | A_23_P202860 | 1.50 |
| GBA2 | glucosidase, beta (bile acid) 2 | A_24_P341187 | 1.50 |
| LOC389834 | ankyrin repeat domain 57 pseudogene | A_33_P3245454 | 1.50 |
| CREB3L4 | cAMP responsive element binding protein 3-like 4 | A_33_P3345708 | 1.50 |
| PQLC3 | PQ loop repeat containing 3 | A_23_P131375 | 1.50 |

**Supplementary Table S2. List of the downregulated genes in KHSRP knockdown cells**

| **Gene Symbol** | **Gene Name** | **Probe ID** | **Fold change (/ control siRNA)** |
| --- | --- | --- | --- |
| KHSRP | KH-type splicing regulatory protein | A_23_P16157 | -10.36 |
| PPP6C | protein phosphatase 6, catalytic subunit | A_24_P81298 | -7.88 |
| CTAG1A | cancer/testis antigen 1A | A_23_P148541 | -7.21 |
| ST7 | suppression of tumorigenicity 7 | A_23_P215735 | -6.52 |
| CXorf56 | chromosome X open reading frame 56 | A_23_P171223 | -6.15 |
| WDR78 | WD repeat domain 78 | A_23_P200670 | -6.06 |
| CXorf56 | chromosome X open reading frame 56 | A_24_P290373 | -6.01 |
| CFL2 | cofilin 2 (muscle) | A_24_P373152 | -5.78 |
| F2R | coagulation factor II (thrombin) receptor | A_23_P213562 | -5.66 |
| ALG2 | ALG2, alpha-1,3/1,6-mannosyltransferase | A_33_P3297050 | -5.40 |
| MTHFS | 5,10-methenyltetrahydrofolate synthetase (5-formyltetrahydrofolate cyclo-ligase) | A_23_P163380 | -5.24 |
| FGFR1OP2 | FGFR1 oncogene partner 2 | A_33_P3369262 | -4.80 |
| HECTD2 | HECT domain containing E3 ubiquitin protein ligase 2 | A_32_P201979 | -4.76 |
| TENM2 | teneurin transmembrane protein 2 | A_33_P3245218 | -4.73 |
| ITGB1 | integrin, beta 1 (fibronectin receptor, beta polypeptide, antigen CD29 includes MDF2, MSK12) | A_23_P104199 | -4.57 |
| BRMS1L | breast cancer metastasis-suppressor 1-like | A_24_P56484 | -4.49 |
| NUFIP1 | nuclear fragile X mental retardation protein interacting protein 1 | A_23_P36939 | -4.42 |
| SCARNA9 | small Cajal body-specific RNA 9 | A_33_P3290443 | -4.38 |
| PPP6C | protein phosphatase 6, catalytic subunit | A_23_P355385 | -4.28 |
| SRSF7 | serine/arginine-rich splicing factor 7 | A_24_P222911 | -4.23 |
| SHROOM2 | shroom family member 2 | A_23_P217755 | -4.19 |
| TIMP2 | TIMP metallopeptidase inhibitor 2 | A_33_P3382177 | -4.15 |
| G3BP2 | GTPase activating protein (SH3 domain) binding protein 2 | A_24_P380132 | -4.14 |
| ERCC4 | excision repair cross-complementation group 4 | A_32_P157481 | -4.07 |
| PPP3CB | protein phosphatase 3, catalytic subunit, beta isozyme | A_33_P3397399 | -4.06 |
| DPY19L1 | dpy-19-like 1 (C. elegans) | A_32_P96134 | -4.01 |
| KIAA1462 | KIAA1462 | A_33_P3293446 | -3.97 |
| C19orf12 | chromosome 19 open reading frame 12 | A_23_P209195 | -3.95 |
| TENM2 | teneurin transmembrane protein 2 | A_24_P299474 | -3.89 |
| STYX | serine/threonine/tyrosine interacting protein | A_32_P109794 | -3.86 |
| WBSCR22 | Williams Beuren syndrome chromosome region 22 | A_33_P3414487 | -3.86 |
| CCDC43 | coiled-coil domain containing 43 | A_24_P39724 | -3.84 |
| CCNA2 | cyclin A2 | A_23_P58321 | -3.84 |
| CRY1 | cryptochrome circadian clock 1 | A_24_P407235 | -3.83 |
| ACKR3 | atypical chemokine receptor 3 | A_23_P131676 | -3.72 |
| EIF4EBP2 | eukaryotic translation initiation factor 4E binding protein 2 | A_23_P115922 | -3.71 |
| AMFR | autocrine motility factor receptor, E3 ubiquitin protein ligase | A_23_P141005 | -3.71 |
| SLC1A3 | solute carrier family 1 (glial high affinity glutamate transporter), member 3 | A_24_P286114 | -3.69 |
| DPYSL5 | dihydropyrimidinase-like 5 | A_33_P3222183 | -3.68 |
| E2F8 | E2F transcription factor 8 | A_23_P35871 | -3.66 |
| MDFIC | MyoD family inhibitor domain containing | A_23_P327022 | -3.61 |
| PCMT1 | protein-L-isoaspartate (D-aspartate) O-methyltransferase | A_24_P140827 | -3.58 |
| CLEC18B | C-type lectin domain family 18, member B | A_23_P206501 | -3.57 |
| SCLT1 | sodium channel and clathrin linker 1 | A_33_P3340404 | -3.57 |
| SORT1 | sortilin 1 | A_24_P325520 | -3.55 |
| RNASE1 | ribonuclease, RNase A family, 1 (pancreatic) | A_23_P48596 | -3.52 |
| PTBP3 | polypyrimidine tract binding protein 3 | A_24_P916614 | -3.45 |
| MED24 | mediator complex subunit 24 | A_23_P124760 | -3.40 |
| EID2 | EP300 interacting inhibitor of differentiation 2 | A_23_P365844 | -3.39 |
| OAF | OAF homolog (Drosophila) | A_24_P391586 | -3.38 |
| C4orf3 | chromosome 4 open reading frame 3 | A_23_P388433 | -3.38 |
| PDE4DIP | phosphodiesterase 4D interacting protein | A_33_P3333982 | -3.37 |
| FBXL3 | F-box and leucine-rich repeat protein 3 | A_23_P140069 | -3.36 |
| ARL2BP | ADP-ribosylation factor-like 2 binding protein | A_23_P15182 | -3.34 |
| ECH1 | enoyl CoA hydratase 1, peroxisomal | A_23_P153853 | -3.33 |
| GDI1 | GDP dissociation inhibitor 1 | A_23_P45496 | -3.29 |
| ZNF251 | zinc finger protein 251 | A_24_P49517 | -3.29 |
| ID2 | inhibitor of DNA binding 2, dominant negative helix-loop-helix protein | A_32_P69368 | -3.25 |
| CERS6 | ceramide synthase 6 | A_33_P3399870 | -3.24 |
| C16orf95 | chromosome 16 open reading frame 95 | A_23_P89030 | -3.24 |
| ELOVL5 | ELOVL fatty acid elongase 5 | A_33_P3273719 | -3.22 |
| DCK | deoxycytidine kinase | A_24_P89080 | -3.22 |
| CPD | carboxypeptidase D | A_24_P53282 | -3.21 |
| GPR176 | G protein-coupled receptor 176 | A_23_P37676 | -3.20 |
| SGTB | small glutamine-rich tetratricopeptide repeat (TPR)-containing, beta | A_33_P3371089 | -3.16 |
| EID2 | EP300 interacting inhibitor of differentiation 2 | A_33_P3284662 | -3.13 |
| RAB28 | RAB28, member RAS oncogene family | A_33_P3353672 | -3.13 |
| SNORA62 | small nucleolar RNA, H/ACA box 62 | A_33_P3334448 | -3.12 |
| DPYSL5 | dihydropyrimidinase-like 5 | A_23_P210224 | -3.09 |
| RTN4 | reticulon 4 | A_33_P3233550 | -3.07 |
| FTL | ferritin, light polypeptide | A_23_P50504 | -3.07 |
| MYBL1 | v-myb avian myeloblastosis viral oncogene homolog-like 1 | A_23_P43157 | -3.06 |
| PCDH19 | protocadherin 19 | A_24_P419039 | -3.06 |
| WRNIP1 | Werner helicase interacting protein 1 | A_24_P192197 | -3.03 |
| CYP1B1 | cytochrome P450, family 1, subfamily B, polypeptide 1 | A_23_P209625 | -3.02 |
| YWHAZ | tyrosine 3-monooxygenase/tryptophan 5-monooxygenase activation protein, zeta | A_32_P198923 | -3.02 |
| MORN2 | MORN repeat containing 2 | A_32_P69465 | -2.98 |
| ID2 | inhibitor of DNA binding 2, dominant negative helix-loop-helix protein | A_23_P143143 | -2.98 |
| FTL | ferritin, light polypeptide | A_32_P155247 | -2.97 |
| SLCO2B1 | solute carrier organic anion transporter family, member 2B1 | A_23_P150768 | -2.97 |
| HEG1 | heart development protein with EGF-like domains 1 | A_32_P166693 | -2.96 |
| SLC25A30 | solute carrier family 25, member 30 | A_33_P3380417 | -2.96 |
| HCFC2 | host cell factor C2 | A_32_P85539 | -2.96 |
| TRIM6 | tripartite motif containing 6 | A_24_P381199 | -2.95 |
| ARHGAP26 | Rho GTPase activating protein 26 | A_33_P3228305 | -2.95 |
| MIR31HG | MIR31 host gene (non-protein coding) | A_33_P3608210 | -2.90 |
| NEIL3 | nei endonuclease VIII-like 3 (E. coli) | A_23_P155711 | -2.88 |
| CA2 | carbonic anhydrase II | A_23_P8913 | -2.88 |
| PSIP1 | PC4 and SFRS1 interacting protein 1 | A_33_P3226605 | -2.86 |
| FOXM1 | forkhead box M1 | A_23_P151150 | -2.86 |
| RABGAP1L | RAB GTPase activating protein 1-like | A_23_P200325 | -2.86 |
| TBCEL | tubulin folding cofactor E-like | A_23_P410017 | -2.85 |
| NPHP1 | nephronophthisis 1 (juvenile) | A_24_P88801 | -2.85 |
| OTUD6B-AS1 | OTUD6B antisense RNA 1 (head to head) | A_19_P00800264 | -2.83 |
| PITPNB | phosphatidylinositol transfer protein, beta | A_24_P305678 | -2.83 |
| ZNRF2 | zinc and ring finger 2, E3 ubiquitin protein ligase | A_33_P3312842 | -2.81 |
| LIN54 | lin-54 DREAM MuvB core complex component | A_33_P3851513 | -2.77 |
| TEF | thyrotrophic embryonic factor | A_24_P151582 | -2.76 |
| TAF13 | TAF13 RNA polymerase II, TATA box binding protein (TBP)-associated factor, 18kDa | A_23_P35148 | -2.76 |
| GAS2L3 | growth arrest-specific 2 like 3 | A_32_P189204 | -2.75 |
| FBXO28 | F-box protein 28 | A_24_P310756 | -2.74 |
| LEPROTL1 | leptin receptor overlapping transcript-like 1 | A_23_P215931 | -2.73 |
| IDI1 | isopentenyl-diphosphate delta isomerase 1 | A_24_P103886 | -2.73 |
| FN3KRP | fructosamine 3 kinase related protein | A_23_P77813 | -2.71 |
| ABCC9 | ATP-binding cassette, sub-family C (CFTR/MRP), member 9 | A_24_P178503 | -2.70 |
| OPA3 | optic atrophy 3 (autosomal recessive, with chorea and spastic paraplegia) | A_33_P3376636 | -2.70 |
| CPEB4 | cytoplasmic polyadenylation element binding protein 4 | A_23_P251937 | -2.69 |
| AGTPBP1 | ATP/GTP binding protein 1 | A_23_P169278 | -2.69 |
| ATG13 | autophagy related 13 | A_24_P295601 | -2.69 |
| ELMOD2 | ELMO/CED-12 domain containing 2 | A_23_P7282 | -2.68 |
| CCP110 | centriolar coiled coil protein 110kDa | A_24_P373286 | -2.66 |
| NEFM | neurofilament, medium polypeptide | A_33_P3249534 | -2.66 |
| YWHAZ | tyrosine 3-monooxygenase/tryptophan 5-monooxygenase activation protein, zeta | A_32_P226149 | -2.66 |
| SMC1A | structural maintenance of chromosomes 1A | A_33_P3290909 | -2.66 |
| CYP1B1 | cytochrome P450, family 1, subfamily B, polypeptide 1 | A_33_P3290343 | -2.66 |
| RPS6KB1 | ribosomal protein S6 kinase, 70kDa, polypeptide 1 | A_24_P497226 | -2.65 |
| RPS26 | ribosomal protein S26 | A_23_P139471 | -2.64 |
| PTPRD | protein tyrosine phosphatase, receptor type, D | A_33_P3309551 | -2.61 |
| MYO10 | myosin X | A_33_P3341424 | -2.61 |
| C6orf58 | chromosome 6 open reading frame 58 | A_23_P93602 | -2.60 |
| NEK4 | NIMA-related kinase 4 | A_24_P80338 | -2.60 |
| DCAF12 | DDB1 and CUL4 associated factor 12 | A_23_P255257 | -2.60 |
| ERC2 | ELKS/RAB6-interacting/CAST family member 2 | A_24_P205045 | -2.59 |
| NCAPG | non-SMC condensin I complex, subunit G | A_33_P3230254 | -2.58 |
| CYP27A1 | cytochrome P450, family 27, subfamily A, polypeptide 1 | A_33_P3361422 | -2.58 |
| KPNA4 | karyopherin alpha 4 (importin alpha 3) | A_19_P00802654 | -2.58 |
| NDUFC2 | NADH dehydrogenase (ubiquinone) 1, subcomplex unknown, 2, 14.5kDa | A_24_P364236 | -2.57 |
| KRT86 | keratin 86, type II | A_23_P363769 | -2.57 |
| PMF1-BGLAP | PMF1-BGLAP readthrough | A_33_P3323481 | -2.56 |
| GFPT1 | glutamine--fructose-6-phosphate transaminase 1 | A_23_P345887 | -2.53 |
| GLIPR2 | GLI pathogenesis-related 2 | A_23_P414913 | -2.51 |
| PABPC1 | poly(A) binding protein, cytoplasmic 1 | A_23_P82693 | -2.51 |
| ALG13 | ALG13, UDP-N-acetylglucosaminyltransferase subunit | A_23_P22672 | -2.50 |
| PLK4 | polo-like kinase 4 | A_23_P155969 | -2.48 |
| APOLD1 | apolipoprotein L domain containing 1 | A_23_P48217 | -2.48 |
| DDX21 | DEAD (Asp-Glu-Ala-Asp) box helicase 21 | A_33_P3287815 | -2.47 |
| CCT2 | chaperonin containing TCP1, subunit 2 (beta) | A_23_P105392 | -2.47 |
| PELI1 | pellino E3 ubiquitin protein ligase 1 | A_33_P3316928 | -2.46 |
| NEK2 | NIMA-related kinase 2 | A_24_P319613 | -2.45 |
| KDM4D | lysine (K)-specific demethylase 4D | A_23_P127406 | -2.44 |
| CHP1 | calcineurin-like EF-hand protein 1 | A_23_P112801 | -2.43 |
| EZH2 | enhancer of zeste 2 polycomb repressive complex 2 subunit | A_33_P3252196 | -2.43 |
| C1orf52 | chromosome 1 open reading frame 52 | A_24_P195037 | -2.42 |
| SLC5A3 | solute carrier family 5 (sodium/myo-inositol cotransporter), member 3 | A_33_P3277110 | -2.41 |
| ZNF678 | zinc finger protein 678 | A_19_P00803342 | -2.41 |
| RB1 | retinoblastoma 1 | A_23_P204850 | -2.41 |
| ZBTB1 | zinc finger and BTB domain containing 1 | A_23_P99693 | -2.41 |
| PALM3 | paralemmin 3 | A_33_P3258056 | -2.40 |
| CBX8 | chromobox homolog 8 | A_23_P55421 | -2.40 |
| RCBTB1 | regulator of chromosome condensation (RCC1) and BTB (POZ) domain containing protein 1 | A_23_P117163 | -2.39 |
| APOBEC3F | apolipoprotein B mRNA editing enzyme, catalytic polypeptide-like 3F | A_33_P3262181 | -2.39 |
| ADCY10P1 | adenylate cyclase 10 (soluble) pseudogene 1 | A_24_P303420 | -2.38 |
| LRRC3 | leucine rich repeat containing 3 | A_33_P3263232 | -2.38 |
| ACTG2 | actin, gamma 2, smooth muscle, enteric | A_23_P39955 | -2.38 |
| NOVA2 | neuro-oncological ventral antigen 2 | A_33_P3347707 | -2.38 |
| CCDC146 | coiled-coil domain containing 146 | A_23_P168771 | -2.38 |
| SRSF4 | serine/arginine-rich splicing factor 4 | A_23_P126197 | -2.37 |
| TMEM38B | transmembrane protein 38B | A_23_P60259 | -2.37 |
| SEC24A | SEC24 family member A | A_33_P3331085 | -2.37 |
| PARPBP | PARP1 binding protein | A_33_P3901921 | -2.36 |
| PPP3CB | protein phosphatase 3, catalytic subunit, beta isozyme | A_24_P98524 | -2.36 |
| SCARNA16 | small Cajal body-specific RNA 16 | A_19_P00315843 | -2.36 |
| MCMBP | minichromosome maintenance complex binding protein | A_23_P202594 | -2.36 |
| IREB2 | iron-responsive element binding protein 2 | A_33_P3221528 | -2.36 |
| GKAP1 | G kinase anchoring protein 1 | A_23_P94546 | -2.35 |
| ZNF678 | zinc finger protein 678 | A_19_P00317449 | -2.35 |
| MESDC2 | mesoderm development candidate 2 | A_33_P3244317 | -2.35 |
| SCAF11 | SR-related CTD-associated factor 11 | A_33_P3219469 | -2.33 |
| NEK2 | NIMA-related kinase 2 | A_23_P35219 | -2.33 |
| PLCB1 | phospholipase C, beta 1 (phosphoinositide-specific) | A_24_P941643 | -2.33 |
| LAPTM4A | lysosomal protein transmembrane 4 alpha | A_23_P90659 | -2.32 |
| ZAK | sterile alpha motif and leucine zipper containing kinase AZK | A_23_P318300 | -2.32 |
| TRAPPC1 | trafficking protein particle complex 1 | A_23_P125348 | -2.32 |
| GOLM1 | golgi membrane protein 1 | A_33_P3231653 | -2.32 |
| CMTM8 | CKLF-like MARVEL transmembrane domain containing 8 | A_23_P40880 | -2.31 |
| GNAI1 | guanine nucleotide binding protein (G protein), alpha inhibiting activity polypeptide 1 | A_23_P122976 | -2.31 |
| CMSS1 | cms1 ribosomal small subunit homolog (yeast) | A_23_P132874 | -2.31 |
| SCARNA16 | small Cajal body-specific RNA 16 | A_33_P3286621 | -2.31 |
| SENP5 | SUMO1/sentrin specific peptidase 5 | A_32_P32653 | -2.30 |
| CYP24A1 | cytochrome P450, family 24, subfamily A, polypeptide 1 | A_23_P28815 | -2.30 |
| ARHGAP44 | Rho GTPase activating protein 44 | A_23_P26854 | -2.29 |
| PAIP2 | poly(A) binding protein interacting protein 2 | A_23_P213754 | -2.29 |
| SNRPA1 | small nuclear ribonucleoprotein polypeptide A' | A_32_P28685 | -2.29 |
| CCNE1 | cyclin E1 | A_23_P209200 | -2.29 |
| MSL3P1 | male-specific lethal 3 homolog (Drosophila) pseudogene 1 | A_32_P235159 | -2.28 |
| SCARNA17 | small Cajal body-specific RNA 17 | A_32_P37592 | -2.28 |
| ZNF678 | zinc finger protein 678 | A_33_P3243667 | -2.27 |
| PI4K2A | phosphatidylinositol 4-kinase type 2 alpha | A_24_P244162 | -2.27 |
| IP6K2 | inositol hexakisphosphate kinase 2 | A_23_P301138 | -2.26 |
| TMEM98 | transmembrane protein 98 | A_23_P27035 | -2.26 |
| PHF19 | PHD finger protein 19 | A_23_P401904 | -2.24 |
| LRRC58 | leucine rich repeat containing 58 | A_32_P190049 | -2.23 |
| IFT88 | intraflagellar transport 88 | A_23_P48339 | -2.23 |
| NEK4 | NIMA-related kinase 4 | A_23_P121276 | -2.23 |
| SRRM3 | serine/arginine repetitive matrix 3 | A_33_P3257451 | -2.23 |
| KPNA4 | karyopherin alpha 4 (importin alpha 3) | A_19_P00813347 | -2.23 |
| KRTAP2-2 | keratin associated protein 2-2 | A_33_P3286196 | -2.22 |
| FAM136A | family with sequence similarity 136, member A | A_23_P108641 | -2.22 |
| NEDD4 | neural precursor cell expressed, developmentally down-regulated 4, E3 ubiquitin protein ligase | A_32_P160883 | -2.21 |
| ZNF678 | zinc finger protein 678 | A_19_P00808213 | -2.21 |
| BUB1 | BUB1 mitotic checkpoint serine/threonine kinase | A_23_P124417 | -2.21 |
| AURKA | aurora kinase A | A_23_P131866 | -2.21 |
| DACT3 | dishevelled-binding antagonist of beta-catenin 3 | A_23_P360964 | -2.21 |
| CEP162 | centrosomal protein 162kDa | A_23_P145424 | -2.20 |
| CDC25C | cell division cycle 25C | A_23_P70249 | -2.19 |
| FGFR1OP2 | FGFR1 oncogene partner 2 | A_23_P390734 | -2.19 |
| CENPA | centromere protein A | A_24_P413884 | -2.19 |
| CASK | calcium/calmodulin-dependent serine protein kinase (MAGUK family) | A_32_P168464 | -2.19 |
| SCAF11 | SR-related CTD-associated factor 11 | A_33_P3283196 | -2.19 |
| PTBP3 | polypyrimidine tract binding protein 3 | A_33_P3315929 | -2.18 |
| HELB | helicase (DNA) B | A_23_P2294 | -2.18 |
| C11orf58 | chromosome 11 open reading frame 58 | A_24_P675386 | -2.18 |
| CCDC18 | coiled-coil domain containing 18 | A_33_P3327165 | -2.17 |
| MCAM | melanoma cell adhesion molecule | A_23_P162171 | -2.17 |
| AGTPBP1 | ATP/GTP binding protein 1 | A_33_P3334185 | -2.17 |
| FAM206A | family with sequence similarity 206, member A | A_23_P216568 | -2.17 |
| GPR110 | G protein-coupled receptor 110 | A_23_P214267 | -2.16 |
| HIST1H2AI | histone cluster 1, H2ai | A_33_P3393135 | -2.16 |
| NEK1 | NIMA-related kinase 1 | A_32_P68050 | -2.15 |
| ALDH4A1 | aldehyde dehydrogenase 4 family, member A1 | A_32_P192970 | -2.15 |
| GPR162 | G protein-coupled receptor 162 | A_33_P3243439 | -2.15 |
| CDKN3 | cyclin-dependent kinase inhibitor 3 | A_33_P3307903 | -2.15 |
| ZKSCAN2 | zinc finger with KRAB and SCAN domains 2 | A_32_P64096 | -2.14 |
| IL37 | interleukin 37 | A_23_P5654 | -2.14 |
| NR2C2AP | nuclear receptor 2C2-associated protein | A_24_P126181 | -2.14 |
| PREPL | prolyl endopeptidase-like | A_24_P943193 | -2.13 |
| FAM167A | family with sequence similarity 167, member A | A_23_P334955 | -2.13 |
| RAD51AP1 | RAD51 associated protein 1 | A_23_P99292 | -2.12 |
| MTCH2 | mitochondrial carrier 2 | A_24_P934563 | -2.11 |
| SPRR2C | small proline-rich protein 2C (pseudogene) | A_23_P126089 | -2.11 |
| PARPBP | PARP1 binding protein | A_23_P87769 | -2.11 |
| KHDRBS1 | KH domain containing, RNA binding, signal transduction associated 1 | A_23_P200386 | -2.11 |
| TMBIM1 | transmembrane BAX inhibitor motif containing 1 | A_23_P79331 | -2.11 |
| ARL6IP1 | ADP-ribosylation factor-like 6 interacting protein 1 | A_23_P118150 | -2.11 |
| ABAT | 4-aminobutyrate aminotransferase | A_33_P3268487 | -2.10 |
| CMTM1 | CKLF-like MARVEL transmembrane domain containing 1 | A_23_P106661 | -2.10 |
| NAA30 | N(alpha)-acetyltransferase 30, NatC catalytic subunit | A_23_P25868 | -2.10 |
| GLIS3 | GLIS family zinc finger 3 | A_23_P384023 | -2.10 |
| NAT14 | N-acetyltransferase 14 (GCN5-related, putative) | A_23_P50389 | -2.09 |
| CCNB1 | cyclin B1 | A_33_P3401621 | -2.08 |
| CRK | v-crk avian sarcoma virus CT10 oncogene homolog | A_24_P270814 | -2.08 |
| ARHGAP19 | Rho GTPase activating protein 19 | A_23_P1387 | -2.08 |
| XLOC_l2_015464 | | A_33_P3354296 | -2.08 |
| RACGAP1 | Rac GTPase activating protein 1 | A_32_P186474 | -2.07 |
| EVI5 | ecotropic viral integration site 5 | A_24_P96593 | -2.07 |
| ENPP1 | ectonucleotide pyrophosphatase/phosphodiesterase 1 | A_32_P192376 | -2.07 |
| CDCA3 | cell division cycle associated 3 | A_24_P218979 | -2.07 |
| ALG9 | ALG9, alpha-1,2-mannosyltransferase | A_24_P795594 | -2.07 |
| AHCTF1 | AT hook containing transcription factor 1 | A_24_P28657 | -2.07 |
| SNX16 | sorting nexin 16 | A_24_P320880 | -2.06 |
| MTL5 | metallothionein-like 5, testis-specific (tesmin) | A_23_P161507 | -2.06 |
| RABEPK | Rab9 effector protein with kelch motifs | A_24_P386323 | -2.06 |
| MLXIP | MLX interacting protein | A_24_P415624 | -2.05 |
| GOLM1 | golgi membrane protein 1 | A_23_P146512 | -2.05 |
| TMX3 | thioredoxin-related transmembrane protein 3 | A_33_P3252141 | -2.05 |
| FNTA | farnesyltransferase, CAAX box, alpha | A_23_P24926 | -2.05 |
| FAM72A | family with sequence similarity 72, member A | A_33_P3242952 | -2.05 |
| CCNB1 | cyclin B1 | A_23_P122197 | -2.04 |
| CALML4 | calmodulin-like 4 | A_23_P129188 | -2.03 |
| TIMM23B | translocase of inner mitochondrial membrane 23 homolog B (yeast) | A_32_P840463 | -2.03 |
| FAM64A | family with sequence similarity 64, member A | A_33_P3276918 | -2.03 |
| LETM2 | leucine zipper-EF-hand containing transmembrane protein 2 | A_23_P348264 | -2.03 |
| GABRG2 | gamma-aminobutyric acid (GABA) A receptor, gamma 2 | A_32_P25514 | -2.03 |
| HIST1H2BF | histone cluster 1, H2bf | A_33_P3229122 | -2.02 |
| HIST1H2AC | histone cluster 1, H2ac | A_23_P167983 | -2.02 |
| CRYGS | crystallin, gamma S | A_23_P132738 | -2.02 |
| NCEH1 | neutral cholesterol ester hydrolase 1 | A_23_P132644 | -2.02 |
| KIF20A | kinesin family member 20A | A_23_P256956 | -2.01 |
| HOXB6 | homeobox B6 | A_23_P66682 | -2.01 |
| RABGGTB | Rab geranylgeranyltransferase, beta subunit | A_33_P3311618 | -2.01 |
| SEC24D | SEC24 family member D | A_23_P159382 | -2.01 |
| CBLB | Cbl proto-oncogene B, E3 ubiquitin protein ligase | A_23_P212715 | -2.01 |
| RRP15 | ribosomal RNA processing 15 homolog (S. cerevisiae) | A_32_P221822 | -2.00 |
| CERS6 | ceramide synthase 6 | A_32_P5480 | -2.00 |
| ACTR10 | actin-related protein 10 homolog (S. cerevisiae) | A_33_P3416946 | -2.00 |
| OBSCN | obscurin, cytoskeletal calmodulin and titin-interacting RhoGEF | A_24_P273157 | -2.00 |
| HAUS1 | HAUS augmin-like complex, subunit 1 | A_23_P413796 | -2.00 |
| METTL10 | methyltransferase like 10 | A_24_P86868 | -2.00 |
| CCDC85C | coiled-coil domain containing 85C | A_23_P37391 | -1.99 |
| RHOQ | ras homolog family member Q | A_33_P3327921 | -1.99 |
| ZNF706 | zinc finger protein 706 | A_24_P235305 | -1.99 |
| RSBN1 | round spermatid basic protein 1 | A_33_P3888365 | -1.98 |
| GALM | galactose mutarotase (aldose 1-epimerase) | A_23_P67971 | -1.98 |
| AMIGO2 | adhesion molecule with Ig-like domain 2 | A_23_P14083 | -1.98 |
| CLDN3 | claudin 3 | A_33_P3285565 | -1.98 |
| SRRM3 | serine/arginine repetitive matrix 3 | A_23_P331700 | -1.98 |
| MKRN2 | makorin ring finger protein 2 | A_23_P57807 | -1.98 |
| ADAMTS1 | ADAM metallopeptidase with thrombospondin type 1 motif, 1 | A_23_P211039 | -1.98 |
| CCDC186 | coiled-coil domain containing 186 | A_24_P942694 | -1.98 |
| HIST1H2AC | histone cluster 1, H2ac | A_33_P3344127 | -1.98 |
| LIMD1 | LIM domains containing 1 | A_33_P3234015 | -1.97 |
| PURA | purine-rich element binding protein A | A_33_P3411296 | -1.97 |
| LIPE | lipase, hormone-sensitive | A_33_P3294986 | -1.97 |
| GCH1 | GTP cyclohydrolase 1 | A_24_P167642 | -1.97 |
| SLC35E3 | solute carrier family 35, member E3 | A_23_P159110 | -1.97 |
| PACSIN1 | protein kinase C and casein kinase substrate in neurons 1 | A_23_P258088 | -1.97 |
| CHSY1 | chondroitin sulfate synthase 1 | A_23_P37484 | -1.97 |
| NDC80 | NDC80 kinetochore complex component | A_23_P50108 | -1.97 |
| GAS2L2 | growth arrest-specific 2 like 2 | A_33_P3297244 | -1.96 |
| TMPO | thymopoietin | A_23_P325040 | -1.96 |
| RBM17 | RNA binding motif protein 17 | A_23_P35645 | -1.96 |
| RBSN | rabenosyn, RAB effector | A_24_P85181 | -1.96 |
| ING3 | inhibitor of growth family, member 3 | A_33_P3356711 | -1.96 |
| UTRN | utrophin | A_32_P524014 | -1.96 |
| KBTBD6 | kelch repeat and BTB (POZ) domain containing 6 | A_33_P3268174 | -1.95 |
| LINC01119 | long intergenic non-protein coding RNA 1119 | A_33_P3361152 | -1.95 |
| RPS6KA2 | ribosomal protein S6 kinase, 90kDa, polypeptide 2 | A_33_P3347452 | -1.95 |
| DSTYK | dual serine/threonine and tyrosine protein kinase | A_32_P71943 | -1.95 |
| HMMR | hyaluronan-mediated motility receptor (RHAMM) | A_23_P70007 | -1.94 |
| CDKN3 | cyclin-dependent kinase inhibitor 3 | A_23_P48669 | -1.94 |
| STARD13 | StAR-related lipid transfer (START) domain containing 13 | A_23_P342727 | -1.94 |
| FANCA | Fanconi anemia, complementation group A | A_33_P3286422 | -1.94 |
| RACGAP1P | Rac GTPase activating protein 1 pseudogene | A_23_P65041 | -1.94 |
| SBDS | Shwachman-Bodian-Diamond syndrome | A_33_P3238866 | -1.93 |
| RAB27A | RAB27A, member RAS oncogene family | A_24_P373174 | -1.93 |
| KLF13 | Kruppel-like factor 13 | A_32_P197489 | -1.93 |
| LOC100506860 | uncharacterized LOC100506860 | A_19_P00316341 | -1.93 |
| KCNMB4 | potassium channel subfamily M regulatory beta subunit 4 | A_23_P64792 | -1.92 |
| ABCD3 | ATP-binding cassette, sub-family D (ALD), member 3 | A_33_P3307957 | -1.92 |
| NAA30 | N(alpha)-acetyltransferase 30, NatC catalytic subunit | A_24_P66679 | -1.92 |
| SERPINC1 | serpin peptidase inhibitor, clade C (antithrombin), member 1 | A_23_P114626 | -1.92 |
| GAR1 | GAR1 ribonucleoprotein | A_23_P58280 | -1.91 |
| HIST1H2AM | histone cluster 1, H2am | A_32_P221799 | -1.91 |
| CDCA8 | cell division cycle associated 8 | A_23_P375 | -1.91 |
| SPTA1 | spectrin, alpha, erythrocytic 1 | A_23_P63158 | -1.91 |
| RPL27A | ribosomal protein L27a | A_23_P416305 | -1.91 |
| CDC20 | cell division cycle 20 | A_23_P149200 | -1.91 |
| TRNP1 | TMF1-regulated nuclear protein 1 | A_24_P734953 | -1.91 |
| CKAP2 | cytoskeleton associated protein 2 | A_23_P151405 | -1.91 |
| DLGAP5 | discs, large (Drosophila) homolog-associated protein 5 | A_23_P88331 | -1.91 |
| LPGAT1 | lysophosphatidylglycerol acyltransferase 1 | A_23_P46606 | -1.91 |
| CDCA5 | cell division cycle associated 5 | A_23_P104651 | -1.90 |
| GNB1L | guanine nucleotide binding protein (G protein), beta polypeptide 1-like | A_23_P218751 | -1.90 |
| ADAT3 | adenosine deaminase, tRNA-specific 3 | A_33_P3268793 | -1.90 |
| LRRC42 | leucine rich repeat containing 42 | A_24_P276791 | -1.90 |
| C12orf66 | chromosome 12 open reading frame 66 | A_23_P370569 | -1.90 |
| SEC61A2 | Sec61 alpha 2 subunit (S. cerevisiae) | A_24_P415280 | -1.90 |
| TERC | telomerase RNA component | A_33_P3245248 | -1.90 |
| TEAD1 | TEA domain family member 1 (SV40 transcriptional enhancer factor) | A_23_P362893 | -1.90 |
| PSMD5 | proteasome (prosome, macropain) 26S subunit, non-ATPase, 5 | A_24_P363802 | -1.89 |
| PNN | pinin, desmosome associated protein | A_33_P3218694 | -1.89 |
| PLAC8L1 | PLAC8-like 1 | A_33_P3385436 | -1.89 |
| GNPAT | glyceronephosphate O-acyltransferase | A_23_P85777 | -1.89 |
| PRKAA2 | protein kinase, AMP-activated, alpha 2 catalytic subunit | A_32_P94160 | -1.89 |
| CST6 | cystatin E/M | A_23_P146946 | -1.89 |
| MXRA7 | matrix-remodelling associated 7 | A_24_P925062 | -1.88 |
| VAPA | VAMP (vesicle-associated membrane protein)-associated protein A, 33kDa | A_33_P3419460 | -1.88 |
| FANCC | Fanconi anemia, complementation group C | A_23_P32021 | -1.88 |
| DDIAS | DNA damage-induced apoptosis suppressor | A_23_P429491 | -1.88 |
| DEF8 | differentially expressed in FDCP 8 homolog (mouse) | A_24_P193582 | -1.88 |
| ANAPC13 | anaphase promoting complex subunit 13 | A_32_P16854 | -1.87 |
| CLCN5 | chloride channel, voltage-sensitive 5 | A_33_P3415092 | -1.87 |
| HIST1H2AD | histone cluster 1, H2ad | A_23_P428184 | -1.87 |
| LINC00476 | long intergenic non-protein coding RNA 476 | A_33_P3219245 | -1.87 |
| NR2F1-AS1 | NR2F1 antisense RNA 1 | A_19_P00320141 | -1.87 |
| CCT6A | chaperonin containing TCP1, subunit 6A (zeta 1) | A_32_P54544 | -1.87 |
| PSRC1 | proline/serine-rich coiled-coil 1 | A_23_P46539 | -1.87 |
| RFX5 | regulatory factor X, 5 (influences HLA class II expression) | A_33_P3382162 | -1.87 |
| VASH2 | vasohibin 2 | A_23_P115492 | -1.87 |
| AMMECR1 | Alport syndrome, mental retardation, midface hypoplasia and elliptocytosis chromosomal region gene 1 | A_32_P201773 | -1.87 |
| HIST1H2AB | histone cluster 1, H2ab | A_24_P223384 | -1.87 |
| HIST3H2BB | histone cluster 3, H2bb | A_33_P3229335 | -1.86 |
| PIGK | phosphatidylinositol glycan anchor biosynthesis, class K | A_23_P34307 | -1.86 |
| ASRGL1 | asparaginase like 1 | A_23_P203391 | -1.86 |
| PLK1 | polo-like kinase 1 | A_23_P118174 | -1.86 |
| CKAP2L | cytoskeleton associated protein 2-like | A_23_P388812 | -1.86 |
| PDLIM3 | PDZ and LIM domain 3 | A_23_P110403 | -1.86 |
| EIF4E | eukaryotic translation initiation factor 4E | A_32_P203300 | -1.86 |
| SNAPIN | SNAP-associated protein | A_23_P161022 | -1.85 |
| TRIM37 | tripartite motif containing 37 | A_24_P153853 | -1.85 |
| FBXO28 | F-box protein 28 | A_23_P137578 | -1.85 |
| INF2 | inverted formin, FH2 and WH2 domain containing | A_23_P218131 | -1.85 |
| HIST2H2AA4 | histone cluster 2, H2aa4 | A_23_P309381 | -1.85 |
| ICK | intestinal cell (MAK-like) kinase | A_23_P344988 | -1.84 |
| BMPR2 | bone morphogenetic protein receptor, type II (serine/threonine kinase) | A_24_P753161 | -1.84 |
| DEF8 | differentially expressed in FDCP 8 homolog (mouse) | A_23_P88893 | -1.84 |
| RASA4B | RAS p21 protein activator 4B | A_33_P3342443 | -1.84 |
| METTL10 | methyltransferase like 10 | A_33_P3332066 | -1.84 |
| RANBP2 | RAN binding protein 2 | A_33_P3272390 | -1.84 |
| ABCC3 | ATP-binding cassette, sub-family C (CFTR/MRP), member 3 | A_33_P3298024 | -1.83 |
| EIF4EBP2 | eukaryotic translation initiation factor 4E binding protein 2 | A_24_P115621 | -1.83 |
| XIAP | X-linked inhibitor of apoptosis, E3 ubiquitin protein ligase | A_33_P3246990 | -1.83 |
| DNAJC9 | DnaJ (Hsp40) homolog, subfamily C, member 9 | A_23_P104372 | -1.83 |
| FAM200B | family with sequence similarity 200, member B | A_19_P00809624 | -1.83 |
| SKA2 | spindle and kinetochore associated complex subunit 2 | A_24_P945000 | -1.83 |
| CHPF2 | chondroitin polymerizing factor 2 | A_23_P397055 | -1.83 |
| TMPO | thymopoietin | A_33_P3412613 | -1.82 |
| SPIDR | scaffolding protein involved in DNA repair | A_24_P143686 | -1.82 |
| SHC4 | SHC (Src homology 2 domain containing) family, member 4 | A_32_P234145 | -1.82 |
| SRPRB | signal recognition particle receptor, B subunit | A_23_P80773 | -1.82 |
| DSCR3 | Down syndrome critical region 3 | A_24_P254346 | -1.82 |
| LINC01098 | long intergenic non-protein coding RNA 1098 | A_33_P3310572 | -1.82 |
| MKI67 | marker of proliferation Ki-67 | A_33_P3374205 | -1.82 |
| COMMD2 | COMM domain containing 2 | A_23_P40782 | -1.82 |
| CDH11 | cadherin 11, type 2, OB-cadherin (osteoblast) | A_23_P152305 | -1.82 |
| PNMA2 | paraneoplastic Ma antigen 2 | A_24_P389415 | -1.82 |
| BGLAP | bone gamma-carboxyglutamate (gla) protein | A_24_P336551 | -1.81 |
| WDR4 | WD repeat domain 4 | A_23_P143535 | -1.81 |
| ZNF532 | zinc finger protein 532 | A_33_P3418766 | -1.81 |
| C5orf30 | chromosome 5 open reading frame 30 | A_23_P122007 | -1.81 |
| ZSCAN31 | zinc finger and SCAN domain containing 31 | A_23_P214533 | -1.81 |
| C8orf37 | chromosome 8 open reading frame 37 | A_33_P3293207 | -1.81 |
| PER1 | period circadian clock 1 | A_23_P89589 | -1.80 |
| PIGK | phosphatidylinositol glycan anchor biosynthesis, class K | A_24_P379512 | -1.80 |
| TMEM237 | transmembrane protein 237 | A_23_P370097 | -1.80 |
| ADRB2 | adrenoceptor beta 2, surface | A_23_P145024 | -1.80 |
| TROAP | trophinin associated protein | A_23_P150935 | -1.80 |
| COPG2IT1 | COPG2 imprinted transcript 1 (non-protein coding) | A_33_P3294459 | -1.80 |
| CDCA7 | cell division cycle associated 7 | A_23_P251421 | -1.80 |
| CASC8 | cancer susceptibility candidate 8 (non-protein coding) | A_19_P00322375 | -1.79 |
| RAD21 | RAD21 homolog (S. pombe) | A_23_P20463 | -1.79 |
| NGLY1 | N-glycanase 1 | A_23_P218841 | -1.79 |
| HSD17B6 | hydroxysteroid (17-beta) dehydrogenase 6 | A_23_P25030 | -1.79 |
| PBK | PDZ binding kinase | A_32_P62997 | -1.79 |
| LRRC61 | leucine rich repeat containing 61 | A_33_P3233273 | -1.79 |
| PARD6G | par-6 family cell polarity regulator gamma | A_24_P74070 | -1.79 |
| RAB26 | RAB26, member RAS oncogene family | A_33_P3209229 | -1.79 |
| TUBD1 | tubulin, delta 1 | A_23_P26895 | -1.79 |
| KRTAP2-3 | keratin associated protein 2-3 | A_32_P24376 | -1.79 |
| GRPEL1 | GrpE-like 1, mitochondrial (E. coli) | A_33_P3522525 | -1.79 |
| ATXN1L | ataxin 1-like | A_33_P3369969 | -1.79 |
| CASC8 | cancer susceptibility candidate 8 (non-protein coding) | A_19_P00316198 | -1.79 |
| WASF1 | WAS protein family, member 1 | A_33_P3243812 | -1.78 |
| HIST1H3J | histone cluster 1, H3j | A_23_P93282 | -1.78 |
| PTTG2 | pituitary tumor-transforming 2 | A_23_P18579 | -1.78 |
| ZNF165 | zinc finger protein 165 | A_23_P93269 | -1.78 |
| IL1A | interleukin 1, alpha | A_23_P72096 | -1.78 |
| TCP10L | t-complex 10-like | A_23_P350555 | -1.78 |
| BCL11B | B-cell CLL/lymphoma 11B (zinc finger protein) | A_23_P205738 | -1.78 |
| OPTN | optineurin | A_33_P3333317 | -1.78 |
| TMEM64 | transmembrane protein 64 | A_23_P378690 | -1.77 |
| ORMDL3 | ORMDL sphingolipid biosynthesis regulator 3 | A_23_P129829 | -1.77 |
| KCNJ2 | potassium channel, inwardly rectifying subfamily J, member 2 | A_23_P329261 | -1.77 |
| PRKACB | protein kinase, cAMP-dependent, catalytic, beta | A_33_P3772996 | -1.77 |
| ABCD3 | ATP-binding cassette, sub-family D (ALD), member 3 | A_33_P3307955 | -1.77 |
| NMU | neuromedin U | A_23_P69537 | -1.77 |
| APBB3 | amyloid beta (A4) precursor protein-binding, family B, member 3 | A_33_P3356926 | -1.77 |
| HJURP | Holliday junction recognition protein | A_33_P3807062 | -1.77 |
| KNSTRN | kinetochore-localized astrin/SPAG5 binding protein | A_33_P3224105 | -1.77 |
| ATF7IP2 | activating transcription factor 7 interacting protein 2 | A_19_P00805833 | -1.76 |
| IQCE | IQ motif containing E | A_24_P266285 | -1.76 |
| MEF2A | myocyte enhancer factor 2A | A_33_P3341676 | -1.76 |
| DUS1L | dihydrouridine synthase 1-like (S. cerevisiae) | A_23_P255569 | -1.76 |
| ARHGAP19 | Rho GTPase activating protein 19 | A_23_P334845 | -1.76 |
| PPFIA1 | protein tyrosine phosphatase, receptor type, f polypeptide (PTPRF), interacting protein (liprin), alpha 1 | A_23_P75516 | -1.76 |
| LIMA1 | LIM domain and actin binding 1 | A_23_P151267 | -1.76 |
| CLEC11A | C-type lectin domain family 11, member A | A_33_P3229032 | -1.76 |
| KCTD9 | potassium channel tetramerization domain containing 9 | A_33_P3297399 | -1.75 |
| PTTG1 | pituitary tumor-transforming 1 | A_23_P7636 | -1.75 |
| ZBTB6 | zinc finger and BTB domain containing 6 | A_23_P83234 | -1.75 |
| TPGS2 | tubulin polyglutamylase complex subunit 2 | A_23_P141715 | -1.75 |
| TJP1 | tight junction protein 1 | A_24_P193435 | -1.75 |
| LYG1 | lysozyme G-like 1 | A_23_P165707 | -1.75 |
| ARAF | A-Raf proto-oncogene, serine/threonine kinase | A_33_P3362353 | -1.75 |
| USP13 | ubiquitin specific peptidase 13 (isopeptidase T-3) | A_23_P40989 | -1.75 |
| ATF7IP2 | activating transcription factor 7 interacting protein 2 | A_19_P00809417 | -1.75 |
| HIST1H2BJ | histone cluster 1, H2bj | A_24_P55148 | -1.75 |
| RPS6KB1 | ribosomal protein S6 kinase, 70kDa, polypeptide 1 | A_33_P3362562 | -1.75 |
| CEP72 | centrosomal protein 72kDa | A_23_P302654 | -1.74 |
| TTC9B | tetratricopeptide repeat domain 9B | A_33_P3293593 | -1.74 |
| TCEA1 | transcription elongation factor A (SII), 1 | A_23_P132444 | -1.74 |
| KPNA6 | karyopherin alpha 6 (importin alpha 7) | A_24_P128563 | -1.74 |
| PRKCE | protein kinase C, epsilon | A_23_P250564 | -1.74 |
| DNM3 | dynamin 3 | A_23_P371266 | -1.74 |
| CUL5 | cullin 5 | A_33_P3301286 | -1.74 |
| ORMDL3 | ORMDL sphingolipid biosynthesis regulator 3 | A_23_P38190 | -1.73 |
| TM9SF3 | transmembrane 9 superfamily member 3 | A_24_P309360 | -1.73 |
| TCEA1 | transcription elongation factor A (SII), 1 | A_24_P126651 | -1.73 |
| MOK | MOK protein kinase | A_23_P76731 | -1.73 |
| HIST1H2AM | histone cluster 1, H2am | A_24_P86389 | -1.73 |
| CCNB2 | cyclin B2 | A_23_P65757 | -1.73 |
| KRT32 | keratin 32, type I | A_23_P89601 | -1.73 |
| DUSP26 | dual specificity phosphatase 26 (putative) | A_23_P146134 | -1.73 |
| KRT80 | keratin 80, type II | A_24_P331704 | -1.73 |
| BTBD10 | BTB (POZ) domain containing 10 | A_23_P13438 | -1.73 |
| FANCF | Fanconi anemia, complementation group F | A_23_P12896 | -1.73 |
| PDXK | pyridoxal (pyridoxine, vitamin B6) kinase | A_24_P318967 | -1.73 |
| YPEL5 | yippee-like 5 (Drosophila) | A_33_P3281408 | -1.72 |
| TIA1 | TIA1 cytotoxic granule-associated RNA binding protein | A_23_P90845 | -1.72 |
| PALM3 | paralemmin 3 | A_33_P3258061 | -1.72 |
| IMPAD1 | inositol monophosphatase domain containing 1 | A_24_P166789 | -1.72 |
| SNX6 | sorting nexin 6 | A_23_P54000 | -1.72 |
| FAM83D | family with sequence similarity 83, member D | A_23_P323751 | -1.72 |
| KIAA1671 | KIAA1671 | A_33_P3415062 | -1.72 |
| FMR1 | fragile X mental retardation 1 | A_24_P93967 | -1.72 |
| ANLN | anillin, actin binding protein | A_23_P356684 | -1.72 |
| HDAC8 | histone deacetylase 8 | A_24_P254965 | -1.72 |
| SNX6 | sorting nexin 6 | A_33_P3292679 | -1.71 |
| KCTD9 | potassium channel tetramerization domain containing 9 | A_24_P135406 | -1.71 |
| FAM46B | family with sequence similarity 46, member B | A_23_P12199 | -1.71 |
| KIF5C | kinesin family member 5C | A_32_P154473 | -1.71 |
| UBR7 | ubiquitin protein ligase E3 component n-recognin 7 (putative) | A_24_P159094 | -1.71 |
| RAD1 | RAD1 checkpoint DNA exonuclease | A_23_P144697 | -1.71 |
| LOC100128361 | uncharacterized LOC100128361 | A_32_P93996 | -1.71 |
| DBP | D site of albumin promoter (albumin D-box) binding protein | A_23_P130753 | -1.71 |
| GTSE1 | G-2 and S-phase expressed 1 | A_23_P57588 | -1.71 |
| PARN | poly(A)-specific ribonuclease | A_32_P133244 | -1.71 |
| CXorf57 | chromosome X open reading frame 57 | A_23_P96369 | -1.71 |
| ASPM | asp (abnormal spindle) homolog, microcephaly associated (Drosophila) | A_23_P52017 | -1.70 |
| C8orf37 | chromosome 8 open reading frame 37 | A_33_P3293202 | -1.70 |
| NCAPD2 | non-SMC condensin I complex, subunit D2 | A_33_P3303385 | -1.70 |
| HIST1H2AJ | histone cluster 1, H2aj | A_33_P3344086 | -1.70 |
| HNRNPA3 | heterogeneous nuclear ribonucleoprotein A3 | A_33_P3422170 | -1.70 |
| PRC1 | protein regulator of cytokinesis 1 | A_23_P206059 | -1.70 |
| JKAMP | JNK1/MAPK8-associated membrane protein | A_23_P205584 | -1.70 |
| CMTR2 | cap methyltransferase 2 | A_24_P185036 | -1.69 |
| SYNCRIP | synaptotagmin binding, cytoplasmic RNA interacting protein | A_23_P214798 | -1.69 |
| TRIM59 | tripartite motif containing 59 | A_32_P72341 | -1.69 |
| CENPE | centromere protein E, 312kDa | A_23_P253524 | -1.69 |
| NLRP4 | NLR family, pyrin domain containing 4 | A_33_P3406047 | -1.69 |
| SMPD3 | sphingomyelin phosphodiesterase 3, neutral membrane (neutral sphingomyelinase II) | A_23_P163567 | -1.69 |
| IMPAD1 | inositol monophosphatase domain containing 1 | A_23_P136232 | -1.69 |
| OSBPL6 | oxysterol binding protein-like 6 | A_23_P108823 | -1.69 |
| MANF | mesencephalic astrocyte-derived neurotrophic factor | A_23_P132793 | -1.69 |
| SPTBN1 | spectrin, beta, non-erythrocytic 1 | A_33_P3258467 | -1.69 |
| MAPK14 | mitogen-activated protein kinase 14 | A_24_P283288 | -1.68 |
| RRM1 | ribonucleotide reductase M1 | A_23_P87351 | -1.68 |
| HSPA5 | heat shock 70kDa protein 5 (glucose-regulated protein, 78kDa) | A_24_P98411 | -1.68 |
| PIF1 | PIF1 5'-to-3' DNA helicase | A_23_P416468 | -1.68 |
| SGOL2 | shugoshin-like 2 (S. pombe) | A_23_P411335 | -1.68 |
| ITM2C | integral membrane protein 2C | A_24_P402690 | -1.68 |
| C7orf73 | chromosome 7 open reading frame 73 | A_33_P3422812 | -1.68 |
| DCP1A | decapping mRNA 1A | A_24_P576174 | -1.68 |
| CREG1 | cellular repressor of E1A-stimulated genes 1 | A_33_P3231297 | -1.68 |
| CDCA7 | cell division cycle associated 7 | A_33_P3296169 | -1.68 |
| HSP90AA1 | heat shock protein 90kDa alpha (cytosolic), class A member 1 | A_23_P162874 | -1.68 |
| SCARA3 | scavenger receptor class A, member 3 | A_23_P215900 | -1.67 |
| WASF1 | WAS protein family, member 1 | A_23_P168306 | -1.67 |
| NGRN | neugrin, neurite outgrowth associated | A_23_P140602 | -1.67 |
| AASDHPPT | aminoadipate-semialdehyde dehydrogenase-phosphopantetheinyl transferase | A_23_P202988 | -1.67 |
| HIST4H4 | histone cluster 4, H4 | A_23_P388871 | -1.67 |
| CYP17A1-AS1 | CYP17A1 antisense RNA 1 | A_33_P3236986 | -1.67 |
| RRM2 | ribonucleotide reductase M2 | A_24_P225616 | -1.67 |
| AFF4 | AF4/FMR2 family, member 4 | A_24_P286079 | -1.67 |
| GRPEL2 | GrpE-like 2, mitochondrial (E. coli) | A_23_P404091 | -1.67 |
| MBNL2 | muscleblind-like splicing regulator 2 | A_24_P56317 | -1.67 |
| NUMA1 | nuclear mitotic apparatus protein 1 | A_23_P162120 | -1.67 |
| HIST1H3B | histone cluster 1, H3b | A_23_P93258 | -1.67 |
| STBD1 | starch binding domain 1 | A_23_P254079 | -1.67 |
| LRRC17 | leucine rich repeat containing 17 | A_23_P253958 | -1.67 |
| CENPP | centromere protein P | A_33_P3245321 | -1.67 |
| DSG3 | desmoglein 3 | A_33_P3348747 | -1.67 |
| ASPH | aspartate beta-hydroxylase | A_23_P216094 | -1.66 |
| KHDC1 | KH homology domain containing 1 | A_24_P280762 | -1.66 |
| HEATR5A | HEAT repeat containing 5A | A_33_P3251332 | -1.66 |
| ZNF24 | zinc finger protein 24 | A_24_P920188 | -1.66 |
| SYNJ1 | synaptojanin 1 | A_23_P324718 | -1.66 |
| SMC2 | structural maintenance of chromosomes 2 | A_33_P3357322 | -1.66 |
| CDCA2 | cell division cycle associated 2 | A_23_P385861 | -1.66 |
| SLC11A2 | solute carrier family 11 (proton-coupled divalent metal ion transporter), member 2 | A_24_P381494 | -1.66 |
| SLC25A20 | solute carrier family 25 (carnitine/acylcarnitine translocase), member 20 | A_23_P72025 | -1.66 |
| SCRN3 | secernin 3 | A_23_P17021 | -1.66 |
| BORA | bora, aurora kinase A activator | A_23_P25626 | -1.66 |
| MKI67 | marker of proliferation Ki-67 | A_33_P3374210 | -1.66 |
| LMCD1 | LIM and cysteine-rich domains 1 | A_23_P6771 | -1.66 |
| YPEL5 | yippee-like 5 (Drosophila) | A_23_P108835 | -1.66 |
| RAP1GDS1 | RAP1, GTP-GDP dissociation stimulator 1 | A_33_P3215123 | -1.66 |
| ULK4 | unc-51 like kinase 4 | A_33_P3392892 | -1.65 |
| MIER1 | mesoderm induction early response 1, transcriptional regulator | A_23_P305723 | -1.65 |
| MSL1 | male-specific lethal 1 homolog (Drosophila) | A_33_P3266873 | -1.65 |
| BBS9 | Bardet-Biedl syndrome 9 | A_23_P82351 | -1.65 |
| AAK1 | AP2 associated kinase 1 | A_33_P3210585 | -1.65 |
| SMNDC1 | survival motor neuron domain containing 1 | A_23_P127233 | -1.65 |
| RAD54L | RAD54-like (S. cerevisiae) | A_23_P74115 | -1.65 |
| RAB40B | RAB40B, member RAS oncogene family | A_23_P129801 | -1.65 |
| RNF14 | ring finger protein 14 | A_24_P132039 | -1.65 |
| KCTD17 | potassium channel tetramerization domain containing 17 | A_24_P108779 | -1.64 |
| C2CD3 | C2 calcium-dependent domain containing 3 | A_23_P150741 | -1.64 |
| PAPD4 | PAP associated domain containing 4 | A_23_P393425 | -1.64 |
| CTSA | cathepsin A | A_24_P74371 | -1.64 |
| WRB | tryptophan rich basic protein | A_23_P80122 | -1.64 |
| PSMD5 | proteasome (prosome, macropain) 26S subunit, non-ATPase, 5 | A_33_P3305254 | -1.64 |
| TMEM237 | transmembrane protein 237 | A_24_P941831 | -1.64 |
| TTC38 | tetratricopeptide repeat domain 38 | A_24_P156388 | -1.64 |
| RGPD5 | RANBP2-like and GRIP domain containing 5 | A_23_P218637 | -1.64 |
| RPS23 | ribosomal protein S23 | A_33_P3257714 | -1.64 |
| USP48 | ubiquitin specific peptidase 48 | A_33_P3612740 | -1.63 |
| KIF21A | kinesin family member 21A | A_23_P113462 | -1.63 |
| PHF20 | PHD finger protein 20 | A_24_P297098 | -1.63 |
| EXOSC2 | exosome component 2 | A_23_P216396 | -1.63 |
| TBL2 | transducin (beta)-like 2 | A_23_P134395 | -1.63 |
| TMEM104 | transmembrane protein 104 | A_33_P3282566 | -1.63 |
| TOP2A | topoisomerase (DNA) II alpha 170kDa | A_23_P118834 | -1.63 |
| CHMP3 | charged multivesicular body protein 3 | A_24_P240065 | -1.63 |
| LOC100507165 | uncharacterized LOC100507165 | A_19_P00315649 | -1.63 |
| FAM92A1 | family with sequence similarity 92, member A1 | A_32_P103695 | -1.63 |
| ITM2C | integral membrane protein 2C | A_24_P379820 | -1.63 |
| KIFC1 | kinesin family member C1 | A_23_P133956 | -1.63 |
| HIST1H4D | histone cluster 1, H4d | A_23_P395374 | -1.62 |
| EIF4G2 | eukaryotic translation initiation factor 4 gamma, 2 | A_33_P3306545 | -1.62 |
| TMEM99 | transmembrane protein 99 | A_33_P3407985 | -1.62 |
| HIST1H3D | histone cluster 1, H3d | A_24_P217834 | -1.62 |
| APBB3 | amyloid beta (A4) precursor protein-binding, family B, member 3 | A_23_P110445 | -1.62 |
| IQCB1 | IQ motif containing B1 | A_23_P84140 | -1.62 |
| BIRC5 | baculoviral IAP repeat containing 5 | A_23_P118815 | -1.62 |
| KIF2C | kinesin family member 2C | A_23_P34788 | -1.62 |
| DUS1L | dihydrouridine synthase 1-like (S. cerevisiae) | A_33_P3266530 | -1.62 |
| PCNP | PEST proteolytic signal containing nuclear protein | A_23_P155332 | -1.62 |
| CFAP43 | cilia and flagella associated protein 43 | A_23_P350678 | -1.62 |
| DYNLL2 | dynein, light chain, LC8-type 2 | A_23_P54991 | -1.62 |
| HIST1H3H | histone cluster 1, H3h | A_33_P3287879 | -1.62 |
| SPRR2A | small proline-rich protein 2A | A_33_P3260426 | -1.62 |
| CLSPN | claspin | A_23_P126212 | -1.61 |
| AAMDC | adipogenesis associated, Mth938 domain containing | A_24_P49383 | -1.61 |
| CDC27 | cell division cycle 27 | A_33_P3360718 | -1.61 |
| PANK3 | pantothenate kinase 3 | A_24_P380330 | -1.61 |
| CCAR2 | cell cycle and apoptosis regulator 2 | A_23_P31686 | -1.61 |
| HSP90B1 | heat shock protein 90kDa beta (Grp94), member 1 | A_33_P3408212 | -1.61 |
| KIF22 | kinesin family member 22 | A_33_P3350638 | -1.61 |
| NCAPD2 | non-SMC condensin I complex, subunit D2 | A_23_P25293 | -1.61 |
| TLDC1 | TBC/LysM-associated domain containing 1 | A_24_P213924 | -1.61 |
| COBL | cordon-bleu WH2 repeat protein | A_33_P3725227 | -1.61 |
| TMEM194A | transmembrane protein 194A | A_24_P126628 | -1.61 |
| ZKSCAN5 | zinc finger with KRAB and SCAN domains 5 | A_23_P259663 | -1.61 |
| NDUFV3 | NADH dehydrogenase (ubiquinone) flavoprotein 3, 10kDa | A_23_P211285 | -1.60 |
| MEF2BNB | MEF2B neighbor | A_24_P393470 | -1.60 |
| WRAP73 | WD repeat containing, antisense to TP73 | A_23_P137361 | -1.60 |
| SCARNA9L | small Cajal body-specific RNA 9-like | A_33_P3417459 | -1.60 |
| TMTC1 | transmembrane and tetratricopeptide repeat containing 1 | A_32_P2452 | -1.60 |
| CALM2 | calmodulin 2 (phosphorylase kinase, delta) | A_23_P326170 | -1.60 |
| ZNF516 | zinc finger protein 516 | A_33_P3225690 | -1.60 |
| DDX31 | DEAD (Asp-Glu-Ala-Asp) box polypeptide 31 | A_33_P3305368 | -1.60 |
| PTP4A2 | protein tyrosine phosphatase type IVA, member 2 | A_23_P23114 | -1.60 |
| POMGNT1 | protein O-linked mannose N-acetylglucosaminyltransferase 1 (beta 1,2-) | A_33_P3384543 | -1.60 |
| TMEM59L | transmembrane protein 59-like | A_33_P3302245 | -1.60 |
| FAM204A | family with sequence similarity 204, member A | A_23_P115743 | -1.60 |
| NEURL1B | neuralized E3 ubiquitin protein ligase 1B | A_32_P198731 | -1.60 |
| RIPK4 | receptor-interacting serine-threonine kinase 4 | A_24_P125871 | -1.60 |
| DCP1A | decapping mRNA 1A | A_23_P166826 | -1.60 |
| TNIP3 | TNFAIP3 interacting protein 3 | A_23_P386478 | -1.59 |
| LAMA2 | laminin, alpha 2 | A_23_P70719 | -1.59 |
| TAF1B | TATA box binding protein (TBP)-associated factor, RNA polymerase I, B, 63kDa | A_23_P165891 | -1.59 |
| DCAF6 | DDB1 and CUL4 associated factor 6 | A_23_P160481 | -1.59 |
| HIST1H4L | histone cluster 1, H4l | A_33_P3351851 | -1.59 |
| PGRMC1 | progesterone receptor membrane component 1 | A_33_P3332130 | -1.59 |
| RRAD | Ras-related associated with diabetes | A_24_P262127 | -1.59 |
| SPAG5 | sperm associated antigen 5 | A_23_P89509 | -1.59 |
| EWSR1 | EWS RNA-binding protein 1 | A_23_P91657 | -1.59 |
| SUDS3 | suppressor of defective silencing 3 homolog (S. cerevisiae) | A_23_P334990 | -1.59 |
| HNRNPA3 | heterogeneous nuclear ribonucleoprotein A3 | A_23_P86660 | -1.59 |
| SNHG9 | small nucleolar RNA host gene 9 (non-protein coding) | A_33_P3280965 | -1.59 |
| CALM2 | calmodulin 2 (phosphorylase kinase, delta) | A_33_P3212782 | -1.59 |
| KIF22 | kinesin family member 22 | A_23_P54622 | -1.59 |
| LRRC6 | leucine rich repeat containing 6 | A_23_P112004 | -1.59 |
| HIST1H4E | histone cluster 1, H4e | A_23_P415411 | -1.58 |
| BTBD1 | BTB (POZ) domain containing 1 | A_23_P205830 | -1.58 |
| ZCCHC24 | zinc finger, CCHC domain containing 24 | A_23_P335452 | -1.58 |
| KCTD9 | potassium channel tetramerization domain containing 9 | A_23_P43226 | -1.58 |
| SPEF1 | sperm flagellar 1 | A_33_P3314643 | -1.58 |
| KIAA0232 | KIAA0232 | A_23_P327069 | -1.58 |
| GTF2E1 | general transcription factor IIE, polypeptide 1, alpha 56kDa | A_23_P211748 | -1.58 |
| MYLK2 | myosin light chain kinase 2 | A_23_P80008 | -1.58 |
| ENDOD1 | endonuclease domain containing 1 | A_24_P189533 | -1.58 |
| OXA1L | oxidase (cytochrome c) assembly 1-like | A_23_P2998 | -1.57 |
| MDM2 | MDM2 proto-oncogene, E3 ubiquitin protein ligase | A_19_P00810040 | -1.57 |
| EIF4G2 | eukaryotic translation initiation factor 4 gamma, 2 | A_23_P104892 | -1.57 |
| HN1L | hematological and neurological expressed 1-like | A_23_P434900 | -1.57 |
| CEP55 | centrosomal protein 55kDa | A_23_P115872 | -1.57 |
| DEDD | death effector domain containing | A_23_P74716 | -1.57 |
| SLC35E3 | solute carrier family 35, member E3 | A_23_P147729 | -1.57 |
| TPM2 | tropomyosin 2 (beta) | A_23_P216501 | -1.57 |
| ATP9B | ATPase, class II, type 9B | A_23_P10497 | -1.57 |
| TYMS | thymidylate synthetase | A_23_P50096 | -1.57 |
| DHX40 | DEAH (Asp-Glu-Ala-His) box polypeptide 40 | A_33_P3424112 | -1.57 |
| HIST1H4J | histone cluster 1, H4j | A_23_P30805 | -1.57 |
| SP9 | Sp9 transcription factor | A_33_P3383189 | -1.57 |
| ERBB3 | erb-b2 receptor tyrosine kinase 3 | A_33_P3211569 | -1.57 |
| VAMP7 | vesicle-associated membrane protein 7 | A_23_P22671 | -1.57 |
| ENOPH1 | enolase-phosphatase 1 | A_23_P121806 | -1.57 |
| DLD | dihydrolipoamide dehydrogenase | A_33_P3344579 | -1.56 |
| TACC3 | transforming, acidic coiled-coil containing protein 3 | A_23_P212844 | -1.56 |
| PIP4K2A | phosphatidylinositol-5-phosphate 4-kinase, type II, alpha | A_24_P673786 | -1.56 |
| HIST1H4C | histone cluster 1, H4c | A_33_P3378880 | -1.56 |
| FAM228B | family with sequence similarity 228, member B | A_33_P3362900 | -1.56 |
| ARMC9 | armadillo repeat containing 9 | A_23_P209735 | -1.56 |
| CEP55 | centrosomal protein 55kDa | A_33_P3291831 | -1.56 |
| INSIG2 | insulin induced gene 2 | A_24_P944458 | -1.56 |
| PAFAH1B2 | platelet-activating factor acetylhydrolase 1b, catalytic subunit 2 (30kDa) | A_23_P370142 | -1.56 |
| COPS3 | COP9 signalosome subunit 3 | A_23_P89199 | -1.56 |
| HIST1H4K | histone cluster 1, H4k | A_23_P30813 | -1.56 |
| FAM185A | family with sequence similarity 185, member A | A_23_P336565 | -1.56 |
| CASC8 | cancer susceptibility candidate 8 (non-protein coding) | A_19_P00322691 | -1.56 |
| TRPC1 | transient receptor potential cation channel, subfamily C, member 1 | A_24_P28977 | -1.56 |
| SRSF11 | serine/arginine-rich splicing factor 11 | A_33_P3293114 | -1.56 |
| HIST1H4D | histone cluster 1, H4d | A_33_P3410836 | -1.55 |
| SYNDIG1 | synapse differentiation inducing 1 | A_23_P251043 | -1.55 |
| TMEM55B | transmembrane protein 55B | A_23_P48610 | -1.55 |
| CCDC157 | coiled-coil domain containing 157 | A_33_P3213767 | -1.55 |
| PHF12 | PHD finger protein 12 | A_33_P3252884 | -1.55 |
| MRVI1 | murine retrovirus integration site 1 homolog | A_33_P3311403 | -1.55 |
| KIF14 | kinesin family member 14 | A_33_P3230548 | -1.55 |
| HERPUD1 | homocysteine-inducible, endoplasmic reticulum stress-inducible, ubiquitin-like domain member 1 | A_23_P54846 | -1.55 |
| ALPK1 | alpha-kinase 1 | A_23_P133133 | -1.55 |
| UBE2C | ubiquitin-conjugating enzyme E2C | A_24_P297539 | -1.55 |
| BUB1B | BUB1 mitotic checkpoint serine/threonine kinase B | A_23_P163481 | -1.55 |
| C6orf89 | chromosome 6 open reading frame 89 | A_33_P3215028 | -1.55 |
| LDHC | lactate dehydrogenase C | A_23_P53039 | -1.54 |
| TOP1 | topoisomerase (DNA) I | A_33_P3371493 | -1.54 |
| KPNA6 | karyopherin alpha 6 (importin alpha 7) | A_33_P3214874 | -1.54 |
| MXD3 | MAX dimerization protein 3 | A_33_P3297255 | -1.54 |
| TPX2 | TPX2, microtubule-associated | A_23_P68610 | -1.54 |
| HIST1H4F | histone cluster 1, H4f | A_23_P359540 | -1.54 |
| HSPA2 | heat shock 70kDa protein 2 | A_23_P88303 | -1.54 |
| KIF4A | kinesin family member 4A | A_23_P148475 | -1.54 |
| RFC1 | replication factor C (activator 1) 1, 145kDa | A_23_P18465 | -1.54 |
| TPRG1L | tumor protein p63 regulated 1-like | A_23_P339773 | -1.54 |
| OPA1 | optic atrophy 1 (autosomal dominant) | A_23_P211797 | -1.54 |
| CDKN2D | cyclin-dependent kinase inhibitor 2D (p19, inhibits CDK4) | A_23_P89941 | -1.54 |
| PIKFYVE | phosphoinositide kinase, FYVE finger containing | A_23_P400984 | -1.54 |
| WDR73 | WD repeat domain 73 | A_23_P99917 | -1.53 |
| SEL1L | sel-1 suppressor of lin-12-like (C. elegans) | A_33_P3404701 | -1.53 |
| SMARCD3 | SWI/SNF related, matrix associated, actin dependent regulator of chromatin, subfamily d, member 3 | A_23_P122852 | -1.53 |
| TP53RK | TP53 regulating kinase | A_23_P109055 | -1.53 |
| IRS1 | insulin receptor substrate 1 | A_24_P802145 | -1.53 |
| MRPL10 | mitochondrial ribosomal protein L10 | A_23_P218423 | -1.53 |
| MPZL2 | myelin protein zero-like 2 | A_33_P3215948 | -1.53 |
| HSPB8 | heat shock 22kDa protein 8 | A_23_P162579 | -1.53 |
| WDR62 | WD repeat domain 62 | A_33_P3254606 | -1.53 |
| TARDBP | TAR DNA binding protein | A_23_P403955 | -1.53 |
| HPSE | heparanase | A_23_P256107 | -1.53 |
| ZNF280D | zinc finger protein 280D | A_23_P14708 | -1.53 |
| ADIPOR1 | adiponectin receptor 1 | A_23_P46627 | -1.53 |
| BFSP1 | beaded filament structural protein 1, filensin | A_23_P109171 | -1.53 |
| DNAJC3 | DnaJ (Hsp40) homolog, subfamily C, member 3 | A_33_P3462960 | -1.53 |
| TOP1 | topoisomerase (DNA) I | A_23_P305507 | -1.53 |
| VEZF1 | vascular endothelial zinc finger 1 | A_24_P924591 | -1.53 |
| MSL3 | male-specific lethal 3 homolog (Drosophila) | A_23_P217778 | -1.53 |
| CCDC149 | coiled-coil domain containing 149 | A_33_P3225843 | -1.53 |
| LSM14A | LSM14A, SCD6 homolog A (S. cerevisiae) | A_24_P365327 | -1.52 |
| TTLL7 | tubulin tyrosine ligase-like family member 7 | A_33_P3348164 | -1.52 |
| MIER1 | mesoderm induction early response 1, transcriptional regulator | A_24_P230916 | -1.52 |
| HIST1H2BN | histone cluster 1, H2bn | A_33_P3229067 | -1.52 |
| F3 | coagulation factor III (thromboplastin, tissue factor) | A_33_P3226832 | -1.52 |
| PDS5B | PDS5 cohesin associated factor B | A_23_P205098 | -1.52 |
| SACM1L | SAC1 suppressor of actin mutations 1-like (yeast) | A_23_P212383 | -1.52 |
| C17orf97 | chromosome 17 open reading frame 97 | A_33_P3356320 | -1.52 |
| ZRANB3 | zinc finger, RAN-binding domain containing 3 | A_24_P222997 | -1.52 |
| SSX2IP | synovial sarcoma, X breakpoint 2 interacting protein | A_23_P201376 | -1.52 |
| MRAS | muscle RAS oncogene homolog | A_24_P88850 | -1.52 |
| EFR3B | EFR3 homolog B (S. cerevisiae) | A_32_P83049 | -1.52 |
| MYL6B | myosin, light chain 6B, alkali, smooth muscle and non-muscle | A_23_P2223 | -1.51 |
| PBRM1 | polybromo 1 | A_33_P3330453 | -1.51 |
| HIST2H4B | histone cluster 2, H4b | A_23_P436281 | -1.51 |
| HIST1H4K | histone cluster 1, H4k | A_33_P3299865 | -1.51 |
| SNW1 | SNW domain containing 1 | A_23_P37347 | -1.51 |
| KIF22 | kinesin family member 22 | A_33_P3350634 | -1.51 |
| TROAP | trophinin associated protein | A_33_P3237874 | -1.51 |
| HNRNPH1 | heterogeneous nuclear ribonucleoprotein H1 (H) | A_24_P320254 | -1.51 |
| RNF5 | ring finger protein 5, E3 ubiquitin protein ligase | A_23_P8095 | -1.51 |
| AQP3 | aquaporin 3 (Gill blood group) | A_23_P112482 | -1.51 |
| RHBDD1 | rhomboid domain containing 1 | A_24_P134834 | -1.51 |
| CDK19 | cyclin-dependent kinase 19 | A_33_P3328026 | -1.51 |
| HINFP | histone H4 transcription factor | A_24_P121171 | -1.51 |
| N4BP2 | NEDD4 binding protein 2 | A_23_P309779 | -1.50 |
| SKA3 | spindle and kinetochore associated complex subunit 3 | A_33_P3216008 | -1.50 |
| NDNL2 | necdin-like 2 | A_24_P124973 | -1.50 |
| SAMD4A | sterile alpha motif domain containing 4A | A_24_P383523 | -1.50 |
| FUT4 | fucosyltransferase 4 (alpha (1,3) fucosyltransferase, myeloid-specific) | A_33_P3286536 | -1.50 |
| RUNDC1 | RUN domain containing 1 | A_24_P395621 | -1.50 |

**Supplementary Table S3. List of the predicted mRNAs for each miRNA**

has-miR-130b-3p

| **Gene Name** | **Fold change (/ control siRNA)** | **Interactions** |
| --- | --- | --- |
| DSEL | 2.25 | HITS-CLIP |
| FAM178A | 2.24 | Multiple |
| VWA9 | 2.21 | PAR-CLIP |
| ZBTB47 | 2.18 | HITS-CLIP |
| GPR63 | 2.07 | PAR-CLIP |
| INHBA | 2.04 | PAR-CLIP |
| ARID3A | 1.99 | HITS-CLIP |
| ZBTB7A | 1.98 | Multiple |
| NEO1 | 1.93 | HITS-CLIP |
| RAB14 | 1.88 | Multiple |
| EPHA4 | 1.87 | Multiple |
| SESN2 | 1.86 | HITS-CLIP |
| FJX1 | 1.81 | Multiple |
| MGAT4A | 1.80 | Multiple |
| SPOPL | 1.78 | Multiple |
| KLLN | 1.76 | PAR-CLIP |
| GGCT | 1.72 | HITS-CLIP |
| HMGA2 | 1.70 | HITS-CLIP |
| ENPP5 | 1.70 | Multiple |
| HIC2 | 1.69 | HITS-CLIP |
| PRKAA1 | 1.66 | Multiple |
| WDR81 | 1.62 | HITS-CLIP |
| PIK3R2 | 1.61 | PAR-CLIP |
| RNF145 | 1.60 | Other |
| CREB5 | 1.60 | Multiple |
| BMP6 | 1.58 | PAR-CLIP |
| CD2AP | 1.58 | Multiple |
| LTBP1 | 1.57 | HITS-CLIP |
| ADCY7 | 1.56 | PAR-CLIP |
| SLC46A1 | 1.56 | Multiple |
| SDC4 | 1.55 | HITS-CLIP |
| TUB | 1.54 | PAR-CLIP |
| DICER1 | 1.53 | Multiple |
| DDX6 | 1.53 | Multiple |
| GAREM | 1.52 | PAR-CLIP |
| MREG | 1.52 | Multiple |
| DNAJB9 | 1.52 | PAR-CLIP |
| EZH1 | 1.52 | Other |
| TSHZ1 | 1.51 | HITS-CLIP |

has-miR-21-5p

| **Gene Name** | **Fold change (/ control siRNA)** | **Interactions** |
| --- | --- | --- |
| SPRYD4 | 3.63 | Multiple |
| G3BP1 | 2.57 | Multiple |
| TIMP3 | 2.32 | Multiple |
| PDCD4 | 2.27 | Multiple |
| Pdcd4 | 2.27 | Other |
| VWA9 | 2.21 | HITS-CLIP |
| ZBTB47 | 2.18 | Multiple |
| RFFL | 2.00 | Multiple |
| FUBP1 | 1.98 | Multiple |
| ZNFX1 | 1.94 | HITS-CLIP |
| PM20D2 | 1.93 | Multiple |
| PLD1 | 1.88 | Multiple |
| EPHA4 | 1.87 | Multiple |
| KDM4B | 1.86 | PAR-CLIP |
| SAMD5 | 1.84 | Multiple |
| SYPL1 | 1.84 | Multiple |
| MGAT4A | 1.80 | Multiple |
| ST6GAL1 | 1.80 | Multiple |
| PNPLA3 | 1.80 | HITS-CLIP |
| PLAG1 | 1.75 | Multiple |
| SNN | 1.73 | Multiple |
| IGF1R | 1.73 | HITS-CLIP |
| PCSK6 | 1.70 | Luciferase Reporter Assay |
| HIC2 | 1.69 | PAR-CLIP |
| PPP1R37 | 1.65 | HITS-CLIP |
| BTG2 | 1.64 | Multiple |
| MXD1 | 1.64 | PAR-CLIP |
| VPS26A | 1.64 | Multiple |
| DOCK6 | 1.63 | Multiple |
| COL5A2 | 1.63 | Multiple |
| RAB22A | 1.62 | Multiple |
| CD2AP | 1.58 | HITS-CLIP |
| TMEM243 | 1.57 | HITS-CLIP |
| ARIH2 | 1.56 | Multiple |
| PTPN21 | 1.56 | HITS-CLIP |
| TPCN2 | 1.56 | PAR-CLIP |
| CTSC | 1.55 | SILAC |
| TMX4 | 1.55 | Other |
| DICER1 | 1.53 | Multiple |
| ETNK1 | 1.52 | Multiple |

has-miR-301a-3p

| **Gene Name** | **Fold change (/ control siRNA)** | **Interactions** |
| --- | --- | --- |
| G3BP1 | 2.57 | Multiple |
| DSEL | 2.25 | HITS-CLIP |
| FAM178A | 2.24 | Multiple |
| VWA9 | 2.21 | PAR-CLIP |
| ZBTB47 | 2.18 | HITS-CLIP |
| GPR63 | 2.07 | PAR-CLIP |
| INHBA | 2.04 | PAR-CLIP |
| ZBTB7A | 1.98 | Multiple |
| ZNFX1 | 1.94 | HITS-CLIP |
| NEO1 | 1.93 | HITS-CLIP |
| RAB14 | 1.88 | Multiple |
| EPHA4 | 1.87 | Multiple |
| SESN2 | 1.86 | HITS-CLIP |
| FJX1 | 1.81 | Multiple |
| MGAT4A | 1.80 | Multiple |
| SPOPL | 1.78 | PAR-CLIP |
| MAPKAP1 | 1.77 | HITS-CLIP |
| KLLN | 1.76 | PAR-CLIP |
| C15orf39 | 1.75 | HITS-CLIP |
| XYLT2 | 1.73 | HITS-CLIP |
| GGCT | 1.72 | HITS-CLIP |
| HMGA2 | 1.70 | HITS-CLIP |
| ENPP5 | 1.70 | Multiple |
| HIC2 | 1.69 | HITS-CLIP |
| PRKAA1 | 1.66 | Multiple |
| WDR81 | 1.62 | HITS-CLIP |
| RAB22A | 1.62 | HITS-CLIP |
| PIK3R2 | 1.61 | PAR-CLIP |
| CREB5 | 1.60 | Multiple |
| MAL2 | 1.59 | Multiple |
| BMP6 | 1.58 | PAR-CLIP |
| CD2AP | 1.58 | PAR-CLIP |
| LTBP1 | 1.57 | HITS-CLIP |
| ADCY7 | 1.56 | PAR-CLIP |
| SLC46A1 | 1.56 | Multiple |
| SDC4 | 1.55 | HITS-CLIP |
| DICER1 | 1.53 | Multiple |
| DDX6 | 1.53 | Multiple |
| GAREM | 1.52 | PAR-CLIP |
| MREG | 1.52 | PAR-CLIP |
| DNAJB9 | 1.52 | Multiple |
| STX3 | 1.52 | HITS-CLIP |
| TSHZ1 | 1.51 | Multiple |

**Supplementary Table S5. List of primer sets used in PCR and qPCR**

| **Gene name** |  | **Sequence** |
| --- | --- | --- |
| **qPCR** |  |  |
| *GAPDH* | Forward | 5´-AGCCACATCGCTCAGACAC-3´ |
|  | Reverse | 5´-GCCCAATACGACCAAATCC-3´ |
| *KHSRP* | Forward | 5´-GCCGCTTACTACGGACAGAC-3´ |
|  | Reverse | 5´-ACATTCATTCGATTCATTGAGC-3´ |
| *BMP6* | Forward | 5´-GCAGAAACATAACCGTGAAGC-3´ |
|  | Reverse | 5´-GCTAAAACTAATTTTGGTGGGTTTT-3´ |
| *BTG2* | Forward | 5´-GCGAGCAGAGGCTTAAGGT-3´ |
|  | Reverse | 5´-GGGAAACCAGTGGTGTTTGTA-3´ |
| *CD2AP* | Forward | 5´-CAACGAATAAGCACCTATGGACT-3´ |
|  | Reverse | 5´-TTTACACTGACGCTTCTTGGTC-3´ |
| *EPHA4* | Forward | 5´-AAGAGGACAGGGACGGAGAG-3´ |
|  | Reverse | 5´-AGTTATCCTTATACCGGTCCATTTT-3´ |
| *EZH1* | Forward | 5´-GAAGTGAAAGAAGAAGATGAGACTTTT-3´ |
|  | Reverse | 5´-ACGGATCCAGGGATCATCT-3´ |
| *LTBP1* | Forward | 5´-TGCTACGAGGGCTACAGGTT-3´ |
|  | Reverse | 5´-GCAGAGGTGTTGGACCTGA-3´ |
| *MXD1* | Forward | 5´-AACAAAAGCCAAATTGCACA-3´ |
|  | Reverse | 5´-ATCCGGATCCTCTCAATGC-3´ |
| *NEO1* | Forward | 5´-AGCTGGAGCCCAACTGATAA-3´ |
|  | Reverse | 5´-AGCTGGAGCCCAACTGATAA-3´ |
| *PDCD4* | Forward | 5´-GAGGTGGATGTGAAAGATCCTAA-3´ |
|  | Reverse | 5´-CCAAAGGCAAAACTACAGTTTCAT-3´ |
| *RFFL* | Forward | 5´-GTGACCCGGCTATACAAGGA-3´ |
|  | Reverse | 5´-TTGGTCTTCGGCACCACT-3´ |
| *SDC4* | Forward | 5´-CCTTGGGCTGTGTTGGAG-3´ |
|  | Reverse | 5´-AACAGACAACAGTGTGAGAGGTG-3´ |
| *TIMP3* | Forward | 5´-GCTGGAGGTCAACAAGTACCA-3´ |
|  | Reverse | 5´-CACAGCCCCGTGTACATCT-3´ |
| *ZEB1* | Forward | 5´-TCAGTGCAGTCTTCTGAACCA-3´ |
|  | Reverse | 5´-GAGGCTGATCATTGTTCTTGG-3´ |
|  |  |  |
| **TaqMan gene expression assay** | |  |
| primary hsa-miR-130b | FAM | Hs03303416_pri |
| primary hsa-miR-21 | FAM | Hs03302625_pri |
| primary hsa-miR-301a | FAM | Hs03303409_pri |
|  |  |  |
| **TaqMan MicroRNA assay** | |  |
| hsa-miR-130b-3p | FAM | #000456 |
| hsa-miR-21-5p | FAM | #000397 |
| hsa-miR-301a-3p | FAM | #000528 |
| hsa-miR-31-5p | FAM | #002279 |
| RNU44 | FAM | #001094 |
|  |  |  |
| **miScript Precursor assay** | |  |
| precursor hsa-miR-130b | SYBR | Hs_mir-130b_1_PR |
| precursor hsa-miR-21 | SYBR | Hs_mir-21_1_PR |
| precursor hsa-miR-301a | SYBR | Hs_mir-301a_1_PR |
|  |  |  |
| **miScript Primer assay** | |  |
| RNU6 | SYBR | Hs_RNU6-2_11 |
|  |  |  |
| **Construction of expression plasmids for KHSRP** | | |
| Full coding KHSRP for pMXs-Neo | Forward^a^ | 5´-AAAAGAATTCGCCACCATGTCGGACTACAGCACGGGAGGACG-3´ |
|  | Reverse^bc^ | 5´-AAAACTCGAG**TTAAGCGTAATCTGGAACATCGTATGGGTA**TT |
|  |  | GAGCCTGCTGCTGTCCCTGCT GC-3´ |
| ^a^*Eco*RI site is underlined |  |  |
| ^b^*Xho*I site is underlined |  |  |
| ^c^HA epitope sequence is bold | |  |
